# Understanding communication in community engagement for maternal and newborn health programmes in low- and middle-income countries: a realist review

**DOI:** 10.1093/heapol/czad078

**Published:** 2023-08-31

**Authors:** Sara Dada, Praveenkumar Aivalli, Aoife De Brún, Maria Barreix, Nachela Chelwa, Zaccheous Mutunga, Bellington Vwalika, Brynne Gilmore

**Affiliations:** UCD Centre for Interdisciplinary Research Education and Innovation in Health Systems (UCD IRIS Centre), University College Dublin, School of Nursing Midwifery and Health Systems , Belfield, Dublin 4, Ireland; School of Nursing Midwifery and Health Systems, University College Dublin, Belfield, Dublin 4, Ireland; UCD Centre for Interdisciplinary Research Education and Innovation in Health Systems (UCD IRIS Centre), University College Dublin, School of Nursing Midwifery and Health Systems , Belfield, Dublin 4, Ireland; School of Nursing Midwifery and Health Systems, University College Dublin, Belfield, Dublin 4, Ireland; UCD Centre for Interdisciplinary Research Education and Innovation in Health Systems (UCD IRIS Centre), University College Dublin, School of Nursing Midwifery and Health Systems , Belfield, Dublin 4, Ireland; School of Nursing Midwifery and Health Systems, University College Dublin, Belfield, Dublin 4, Ireland; UNDP/UNFPA/UNICEF/WHO/World Bank Special Programme of Research, Development and Research Training in Human Reproduction, Department of Sexual and Reproductive Health and Research, World Health Organization, Avenue Appia 20, 1211, Geneva 27, Switzerland; Population Council, 8 Nyerere Rd., Lusaka, Zambia; Concern Worldwide, Westlands Ave, Nairobi, Kenya; Department of Obstetrics and Gynaecology, University of Zambia School of Medicine, Lusaka, Zambia; UCD Centre for Interdisciplinary Research Education and Innovation in Health Systems (UCD IRIS Centre), University College Dublin, School of Nursing Midwifery and Health Systems , Belfield, Dublin 4, Ireland; School of Nursing Midwifery and Health Systems, University College Dublin, Belfield, Dublin 4, Ireland

**Keywords:** Community engagement, maternal health, newborn health, communication, LMIC, theory, realist synthesis

## Abstract

As community engagement (CE) is implemented for sustainable maternal and newborn health (MNH) programming, it is important to determine how these approaches work. Low- and middle-income countries (LMICs) have become a particular focus for MNH CE activities due to their high burden of maternal and neonatal deaths. MNH messaging and communication to engage communities are likely to differ by context, but how these approaches are actually developed and implemented within CE is not well understood. Understanding how communications in CE actually work is vital in the translation of learnings across programmes and to inform future projects. The purpose of this realist review is to describe how, why, to what extent and for whom communications in CE contribute to MNH programming in LMICs. After searching academic databases, grey literature and literature suggested by the expert advisory committee, documents were included if they described the CE communication processes/activities used for MNH programming in an LMIC. Relevant documents were assessed for richness (depth of insight) and rigor (trustworthiness and coherence of data/theories). Data were extracted as context–mechanism–outcome configurations (CMOCs) and synthesized into demi-regularities to contribute to theory refinement. After screening 416 records, 45 CMOCs were extracted from 11 documents. This informed five programme theories explaining that communications in CE for an MNH programme work when: communities are actively involved throughout the programme, the messaging and programme are acceptable, communication sources are trusted, the community has a reciprocal relationship with the programme and the community sees value in the programme. While these findings reflect what is often anecdotally known in CE or acknowledged in communications theory, they have implications for policy, practice and research by highlighting the importance of centring the community’s needs and priorities throughout the stages of developing and implementing communications for CE in MNH.

Key messages

- Specific maternal and newborn health (MNH) messages and how they are communicated to engage communities are likely to differ by context and community priorities.
- The findings from this review informed five theories explaining that communications in community engagement for MNH works when communities are actively involved throughout the programme, the messaging and programme are acceptable, the communication sources are trusted, the community has a reciprocal relationship with the programme and the community sees value or benefit in the programme.
- These theories highlight the importance of centring the community’s needs and priorities throughout the stages of developing and implementing communications.

## Background

In the last few decades, community engagement (CE) has become an increasingly important component of global health interventions and research due to its role in addressing systemic challenges and integrating care systems (Macqueen *et al.*, 2015; Reynolds and Sariola, 2018; Walsh and Johnson, 2018; Alerrt *et al.*, 2019; Haldane *et al.*, 2019; Dada *et al.*, 2021b). CE can be defined as collaborating with communities and stakeholders to work together on issues in order to improve health outcomes and well-being in the community (WHO, 2017a; Gilmore *et al.*, 2023). While the discourse around CE has often emphasized its relevance to ethical considerations in biomedical research, previous systematic reviews have also reported positive outcomes associated with involving communities in health interventions (e.g. mass anti-malarial administration) even with the high heterogeneity and largely qualitative nature of studies (O’Mara-Eves *et al.*, 2013; Adhikari *et al.*, 2016; Dada *et al.*, 2019; Haldane *et al.*, 2019). CE approaches incorporated across health programming include the establishment of community advisory boards for research studies (Tindana *et al.*, 2007; Adhikari *et al.*, 2019) or the involvement of local community groups in conducting and leading emergency outbreak response (e.g. group meetings and contact tracing) (Armstrong-Mensah and Ndiaye, 2018; Dada *et al.*, 2019; Gilmore *et al.*, 2020).

Despite the agreement on the positive implications of CE, the literature relating to which CE approaches are most effective and how to incorporate them across settings is limited (O’Mara-Eves *et al.*, 2013; Dada *et al.*, 2021b; 2022). Proposed frameworks and principles of CE suggest the importance of characteristics such as adaptability, reciprocity, relatability, relationships, respect, trust and transparency (Noorani and Gubb, 2007; Geissler *et al.*, 2008; Lavery *et al.*, 2010; Kolopack *et al.*, 2015; Dada *et al.*, 2019; Gilmore *et al.*, 2020; Schiavo, 2021). One important element of CE is the communication involved in engaging communities and, equally, the communication platforms that CE often provides (Barker *et al.*, 2020). Literature on CE and participation has highlighted the importance of dialogue between communities and implementers (Figueroa *et al.*, 2003; WHO, 2015; Marston *et al.*, 2016; Dada *et al.*, 2019). For example, Lavery *et al.*’s (2010) framework for CE includes clearly presenting the purpose and goals of research to a community as well as providing information overall, which requires communication. For the purpose of this review, ‘CE communication’ is operationalized as the communication between, or amongst, members of a community and health programmers/implementers within a CE approach. This could include not only the dissemination of information at the individual level, such as through sharing key messages to promote a specific behavior, but also the exchange of ideas and emotions at a group level, such as through conversing with stakeholders about programme priorities (Pratt and Boyden, 1985; Aruma, 2018).

In global health, communication plays an integral role in enhancing transparency and accountability, increasing knowledge, encouraging behavior change and building relationships (Rimal and Lapinski, 2009; Mannava *et al.*, 2015; Rahman *et al.*, 2016; Dada *et al.*, 2021a). Several frameworks provide guidelines for effective communication, including the World Health Organization’s (WHO) recommendation that effective communication is accessible, actionable, credible and trusted, relevant, timely and understandable (WHO, 2017b). CE communication should go beyond top-down information provision and may include communication for development (C4D), which emphasizes the community’s participation and ownership in communication delivery (Jenatsch *et al.*, 2016). Despite the availability of these frameworks for effective communication and the understanding that it is important in health interventions, there is little specific discussion on how communication is used, designed and implemented within CE strategies (Dada *et al.*, 2021b; 2022; 2023a). CE and its communications aspects are complex, involving multiple interacting components and spanning a range of levels of involvement (Gilmore *et al.*, 2023). There is no ‘one-size-fits-all’ approach to CE communications as programmes and experiences vary across settings (Lavery *et al.*, 2010; Adhikari *et al.*, 2019).

WHO guidelines, published indicators and standards as well as funding calls have recommended or even required CE in maternal and newborn health (MNH) programming (WHO, 2014; 2015; 2020a; 2020b; UNICEF, 2020; Dada *et al.*, 2022). A range of CE activities have been implemented for MNH, including mass media awareness campaigns, public community meetings and groups conducting participatory learning and action cycles (Dada *et al.*, 2022). While the concepts behind many of these CE approaches are not unique to MNH, there are a number of different actors and stakeholders involved in improving MNH outcomes (Byass, 2018; Adhikari *et al.*, 2020; WHO, 2020a). Additionally, factors such as gender, power and agency may have an influence on MNH, decision-making and care-seeking (Manda-Taylor *et al.*, 2017; Amosse *et al.*, 2023; Vizheh *et al.*, 2023). Low- and middle-income countries (LMICs) have become a setting of particular focus for these MNH CE activities largely due to the fact that this is where the majority of global maternal and neonatal deaths occur (Silver and Singer, 2014; Langer *et al.*, 2015; WHO, 2023). Consequently, a considerable body of literature reports on the effects of CE approaches in LMICs on outcomes such as maternal morbidity/mortality or service uptake (Kc *et al.*, 2011; Colbourn *et al.*, 2013; Rosato, 2015; WHO, 2015; 2016; Serbanescu *et al.*, 2019; Ekirapa-kiracho *et al.*, 2020; Slemming *et al.*, 2021). However, LMICs face different challenges, including higher prevalence of varying infectious diseases, different priorities and less-resourced health systems, leading to an additional level of complexity to consider in the implementation of CE in MNH interventions (Ritchie *et al.*, 2016).

The current knowledge base on CE for MNH is characterized by this complexity and a body of literature that focuses on outcome measures (Head, 2007; Dada *et al.*, 2023a). The literature on CE overall suggests potential factors that influence or inform communications for CE in various settings but provides little insight regarding the generative causation of ‘what’ makes these approaches ‘work’ and ‘how’. The realist review uses context–mechanism–outcome configurations (CMOCs) to describe generative causation of how a system works and which mechanisms in specific contexts lead to intended or unintended outcomes (Linsley *et al.*, 2015; Ashworth *et al.*, 2020). This is appropriate for exploring CE which relies on both individuals and social institutions/networks and the relationships between them. It is also important to consider their interaction with each other and how the order in which these mechanisms ‘fire’ influences outcomes (Adhikari *et al.*, 2019; Dada *et al.*, 2019). Additionally, while the importance of context in the CE literature is unequivocal, there is a lack of evidence on how different contexts may affect outcomes (through the mechanisms they trigger or inhibit) (Lavery *et al.*, 2010; Adhikari *et al.*, 2019). Finally, a realist review provides a useful lens to explore the literature since the purpose and goals of CE communications may vary based on the programme (Adhikari *et al.*, 2020).

The purpose of this realist review is to understand ‘how, why, to what extent, and for whom’ communications in CE contribute to intended and unintended outcomes in MNH programming in LMICs. Documenting how and why these processes and approaches work (or do not work) is important to translate key learnings and inform the development and implementation of future CE communications. This was done through the development and refinement of programme theories (PT) based on reviewing the literature, engaging with experts and drawing on the researcher’s own experience and knowledge (Booth *et al.*, 2018; Dada *et al.*, 2022). This realist review and the findings it presents aim to support future programme designers and implementers working on CE and CE communications.

## Methods

**Figure 1.**
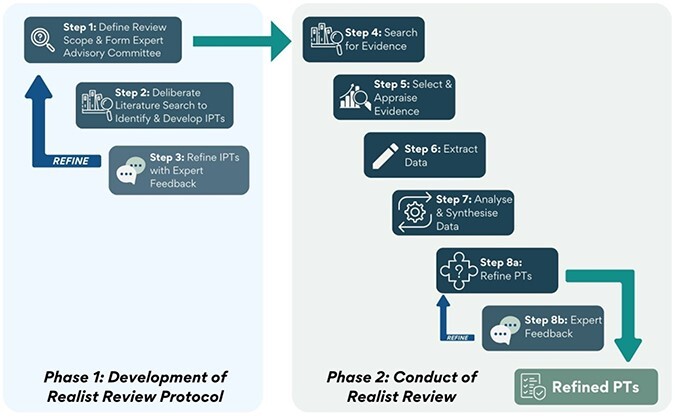
Steps in a realist review: the process followed to conduct this realist review (diagram adapted from Dada *et al.*, 2022). IPT: initial programme theory; PT: programme theory.

A realist review methodology was chosen because it is a theory-driven form of synthesis appropriate for explaining the complexity of programmes implemented in complex and layered systems (Pawson *et al.*, 2005). The Realist And MEta-narrative Evidence Syntheses: Evolving Standards (RAMESES) training materials were used to inform the study design of this review (Wong *et al.*, 2013b), and the RAMESES publication standards guided its conduct and reporting (Wong *et al.*, 2013a) (Supplementary File 1). The protocol was prospectively registered with the International Prospective Register of Systematic Reviews (CRD42022293564) and has been published (Dada *et al.*, 2022). Figure 1 demonstrates the steps involved throughout the realist review process, with Phase 1 completed during the development of the protocol. This article reports on Phase 2.

### Search for evidence

The initial search for evidence in this review was informed by a previous, larger scoping review on CE and programmes involving communities for reproductive, maternal, newborn and child health (Dada *et al.*, 2023a). Informed by published systematic reviews, this search strategy (Supplementary File 2) was last run across seven databases on 1 October 2021 (PubMed/MEDLINE, Embase, CINAHL, PsycINFO, Scopus, Web of Science and Global Health) for records published since 1975 (Prost *et al.*, 2013; De Weger *et al.*, 2018; Adhikari *et al.*, 2019). This time frame was chosen in order to capture examples of CE conducted since the Alma Ata Declaration in 1978. As a starting point, this realist review first screened the documents that were included in the ‘full-text review’ stage from the comprehensive scoping review. A Google Scholar and web search were also conducted for additional articles, reports or records that could contribute to the review using similar terms related to communications and CE. The reference lists of records included in the review were also searched.

### Selection and appraisal of evidence

Records were first screened by two reviewers (SD, PA) by title and abstract and then by full text for relevance (Table 1), with conflicts resolved by a third reviewer (BG). Full-text articles were reviewed in two stages. As reviewers screened the documents, publications that were considered to potentially provide further insight into processes or generative causation around communications for CE were tagged for further review. This is because in a realist review, documents are excluded if they cannot contribute to programme theory (PT) development or refinement (Wong *et al.*, 2013b; Power *et al.*, 2019). Documents that were tagged by either author (*n* = 32) were taken forward for further ‘richness’ assessment. This ‘richness’ assessment (Table 1) was used to determine if documents provided a sufficient level of insight that could be extracted and synthesized to contribute to this review. Article richness was classified as high, moderate, low or none by one reviewer (SD) according to the level and depth of insight regarding generative causation they provided. Any article first classified as ‘none’ was reviewed by a second reviewer (PA) for verification. In line with the RAMESES guidelines for conducting and reporting a realist review, all documents were also appraised for their ‘rigor’, or the trustworthiness and coherence of the data and theories (Wong *et al.*, 2013a; 2013b; Power *et al.*, 2019). No documents were excluded based on rigor; however, this appraisal process was conducted in order to contextualize the contributions of the evidence (Contandriopoulos *et al.*, 2021). This process of appraising evidence in a realist review is often subjective and has been described as transparently as possible in Table 1 (Dada *et al.*, 2023b).

**Table 1.** Relevance, richness and rigor

Relevance	Richness	Rigor
Include: Any study design/article type, except for reviews/meta-analyses Documents ‘about CE being used for MNH programmes, AND’ Describes programmes in an ‘LMIC, AND’ Describes ‘the CE communication activities, processes or tools’ that were used Exclude: Conference proceedings, studies that lack full-text, reviews/meta-analyses (secondary data), OR Documents that are not about MNH programmes or outcomes, OR Documents about programmes in high-income countries, OR Do not describe CE communication activities, processes or tools, or only describe CE that is conducted for the purpose of research	High: Makes several contributions towards theory development. This includes, but is not limited to, insights related to generative causation and/or components of context, mechanisms and outcomes. There is a rich description of processes and/or context so that regardless of quality (rigor), there is sufficient content to build, refine and refute PTs Moderate: Makes one or two contributions towards theory development. Regardless of quality (rigor), content can contribute to inferences relating to PTs Low: Makes little contribution towards theory development and/or results or evidence lack credibility. There is a limited description of potential contexts, mechanisms or outcomes that could contribute to CMOCs and/or theory refining None: While relevant to the review subject matter, papers make no contribution to theory development. It does not include any description of the processes or any content relevant to the initial PTs, or potential contexts, mechanisms or outcomes	The trustworthiness of the data source and coherence of the theory it informed was considered to contextualize the contributions of the evidence. While this did not involve the use of a formalized appraisal tool or checklist, this included the researcher reflecting on and considering the feedback from the expert advisory committee on questions such as: Is the information plausible? Are the CMOCs extracted justifiable? Are the findings put forward coherent? While no documents were excluded based on rigor, this assessment contributes to building credible arguments to test and refine the PTs

The processes of evidence appraisals applied in this review.

### Data extraction

Data extraction was first piloted in one publication (conducted by SD and discussed by BG, ADB, and PA). Document characteristics such as objectives, setting, study design, study participants, CE communications activities, general findings and underlying models/theoretical frameworks were extracted for each paper. The rest of the data extraction form focused on CMOCs by using the information and evidence provided in the publication to detail the contexts, mechanisms and outcomes described to influence or affect the CE communications activities. Contexts (C) are both the observable and relational or dynamic features that can shape, enable or hinder mechanisms (including but not limited to sociocultural, political and geographic factors (Greenhalgh and Manzano, 2022). Mechanisms were operationalized as the invisible interaction between resources that may be introduced by a programme (R1) and the participants’ responses (R2) to them (Dalkin *et al.*, 2015; Project, 2017). The outcomes (O) captured were the (intended or unintended) consequence, or the observable end result of the programme or activity (Hunter *et al.*, 2022). Supplementary File 3 provides the completed data extraction forms.

Data extraction was conducted in phases, following the iterative nature of a realist review. First, the five publications classified as ‘high’ richness were extracted by one reviewer (SD). Two of these publications were double-extracted, and all data extraction forms were reviewed for logic and clarity by a second reviewer (PA). The CMOCs extracted from this first set of papers were reviewed, organized and presented virtually to the external advisory committee for feedback using a list of guiding questions (Supplementary File 4). The ‘moderate’ richness papers were then extracted by one reviewer (SD) and checked independently by a second (PA). These extracted CMOCs were considered and organized alongside the ‘high’ richness CMOCs. ‘Low’ richness papers were then reviewed for any additional data or insight that could contribute to PT refinement (Supplementary File 5).

### Evidence synthesis

Synthesizing the data in this realist review involved searching for demi-regularities across the CMOCs. Demi-regularities are patterns or tendencies found in the data (Fletcher, 2017; Gilmore *et al.*, 2019). By organizing the CMOCs into themes or topic areas (Supplementary File 6), demi-regularities were elucidated through a process similar to coding in a thematic analysis (Fletcher, 2017; Gilmore *et al.*, 2019). These demi-regularities and data were synthesized to inform the refinement and development of overarching PTs. In realist reviews, theory refinement involves utilizing multiple techniques throughout the process of analysing and synthesizing data, including (1) bringing together different sources of evidence, (2) reconciling explanations of different programme outcomes, (3) considering the trustworthiness of included evidence, (4) consolidating evidence to build stronger causal explanations and (5) situating the explanations of different outcomes in different settings (Pearson *et al.*, 2012; Brennan *et al.*, 2014; Power *et al.*, 2019; Hunter *et al.*, 2022).

In practice, the process for this review involved the techniques above by looking at the existing initial programme theories (IPTs) developed in the protocol in Phase 1 (Supplementary File 7) alongside the demi-regularities (Supplementary File 6) and trying to understand the elements of generative causation that were highlighted by them. The reviewers considered where these IPTs and demi-regularities were already aligned and where further expansion or explanation of generative causation was needed. Theories were refined to reflect this information or newly developed if they were not already reflected in the IPTs. The snowballing reference process then served to provide any additional information for theory areas needing potential further explanation. In the process of synthesizing data, literature on substantive theories (largely from the fields of communication and information behavior) was used to further refine theories. This enabled the development of rigorous PTs by ensuring they were analogous and aligned to existing substantive theory.

### Expert advisory committee

No patients or the public were directly involved in this review; however, an expert advisory committee was first formed and engaged in the development of the protocol. This committee included six academics and practitioners based in six countries (India, Kenya, Nepal, Rwanda, Switzerland and Zambia) with experience in CE for MNH. During the protocol development, the expert advisory committee provided insight relating to their own experiences with CE as well as provided feedback on the relevance and resonance of the IPTs. Throughout the conduct of Phase 2 of the realist review, the expert advisory committee was consulted at various stages including for feedback on the CMOCs presented as ‘generative causation statements’ (Supplementary File 3) and on the PTs, shared via Google Sheets and Forms with specific guiding questions and open fields for general feedback (Supplementary File 8). Feedback from the expert advisory committee was used to clarify language and phrasing in the CMOCs and PTs, support CMOCs, inform potential rival CMOCs and check the relevance and applicability of the findings/PTs to lived experience. This input from the expert advisory committee was used to inform and guide the review and was not analysed as data in the review, thereby eliminating the need for ethical approvals.

### Deviations from protocol

Overall, this review was largely conducted as planned out by the protocol. The main difference is that more clarity was developed around the selection and appraisal criteria, namely richness and rigor, as described in Table 1. For example, these considerations of rigor were informed by feedback from the expert advisory committee, which was not originally explained in the protocol. Additionally, using richness criteria to narrow the scope of documents that were extracted for data was not explicit in the protocol. Finally, rather than using NVivo to analyse and synthesize data, the reviewers used a more manual approach to organizing and considering the data (Supplementary File 6).

## Results

**Figure 2.**
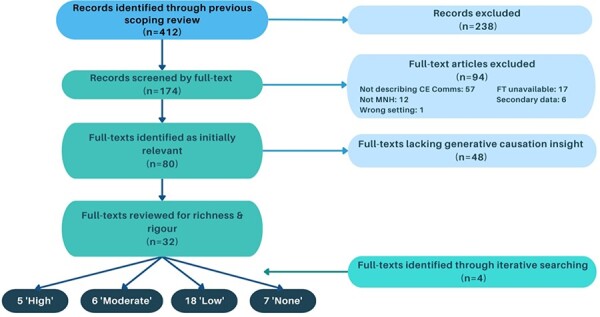
Document selection flow diagram: the selection of documents for inclusion in this realist review

After screening the initial 412 records by title and abstract, 174 records were screened by full text (Figure 2). Of these, 80 were identified as relevant to the subject matter, but 48 of these lacked substantial descriptions of the programme or activities that could be used to understand generative causation. As a result, 32 records were taken forward for richness assessments (Turan *et al.*, 2003; Morrison *et al.*, 2005; 2010; Dongre *et al.*, 2009; Hounton *et al.*, 2009; Azad *et al.*, 2010; Rath *et al.*, 2010; Kaosar, 2012; Prata *et al.*, 2012; Ediau *et al.*, 2013; Cofie *et al.*, 2014; Bayley *et al.*, 2015; Besada *et al.*, 2016; Brasington *et al.*, 2016; Marcil *et al.*, 2016; Woelk *et al.*, 2016; Ekirapa-kiracho *et al.*, 2017; 2020; Manandhar *et al.*, 2004; Dougherty *et al.*, 2018; George *et al.*, 2018; Mochache *et al.*, 2018; Gram *et al.*, 2019; Hamal *et al.*, 2019; Mamo *et al.*, 2019; Serbanescu *et al.*, 2019; Bellad *et al.*, 2020; Butler *et al.*, 2020; Demissie *et al.*, 2020; Edward *et al.*, 2020; Arkedis *et al.*, 2021; Ntoimo *et al.*, 2021). The rigor of the 11 records meeting ‘high’ (Hounton *et al.*, 2009; Rath *et al.*, 2010; Cofie *et al.*, 2014; Marcil *et al.*, 2016; Ntoimo *et al.*, 2021) or ‘moderate’ (Dongre *et al.*, 2009; Morrison *et al.*, 2010; Prata *et al.*, 2012; Besada *et al.*, 2016; Hamal *et al.*, 2019; Butler *et al.*, 2020) richness, and the subsequent 45 unique CMOCs (Table 2) extracted were considered. An additional four records were identified in the iterative searching process and screened for richness; however, they were not included in the data extraction as they did not provide significant levels of depth/insight (Moran *et al.*, 2006; Howard-grabman, 2007; Scott *et al.*, 2017; Okonofua *et al.*, 2022). The 11 included documents were academic peer-reviewed literature that described communications in CE for MNH across Bangladesh, Burkina Faso, Cote d’Ivoire, the Democratic Republic of the Congo, Ghana, India, Malawi, Nepal, Nigeria and Uganda. The CE communications and activities described in these programmes ranged from public gatherings and dramas to recurring women’s groups and community committees (Supplementary File 3 and Supplementary File 9). Outcomes of CE communications spanned a range that included encouraging community’s involvement, participation and/or ownership in a programme (or lack thereof), improving acceptance of a programme or specific messaging, increasing knowledge or awareness, increasing care-seeking or use of health services, developing appropriate programmes or messaging and enabling the sharing of feedback from the community.

**Table 2.** CMOCs

CMOCs	Relevant PTs	Sources
Demi-regularity: empowering and involving the community as actors		
CMOC1: In ‘active’ and motivated communities (C), when communities are consulted and own the programme throughout all stages of the design and implementation (R1), the programme is more acceptable and relatable to the community’s norms and practices (R2). This will make the programme appropriate and applicable to the community’s contexts and structures (O1) and encourage continued participation and ownership in the programme’s implementation (O2).	PT 1: Actively involved PT 2: Acceptable	(Hounton *et al.*, 2009) Additional support (Brasington *et al.*, 2016, Ekirapa-kiracho *et al.*, 2020, Mochache *et al.*, 2018, Okonofua *et al.*, 2022, Moran *et al.*, 2006)
CMOC2: In rural communities (C), community meetings using cyclical learning processes to discuss problems and strategies (R1) develop a sense of belonging and a ‘critical consciousness’ among the community (R2). This drives a wider range of community members to become involved and support the interventions and continue to engage in such discussions (O).	PT 1: Actively involved	(Morrison *et al.*, 2010, Suchitra *et al.*, 2010) Additional support (Ekirapa-kiracho *et al.*, 2020, George *et al.*, 2018, Mochache *et al.*, 2018)
CMOC3: In settings of political instability (C), not engaging with broader sociopolitical structures (R1) means power dynamics/structures are not incorporated in a sustainable way (R2), which inhibits the long-term nature of any potential changes (O).	PT 1: Actively involved	(Morrison *et al.*, 2010)
Demi-regularity: sharing experiences builds knowledge and confidence		
CMOC4: In communities with existing communication platforms where varying actors and decision-makers can interact (C), sharing personal stories and experiences (R1) motivates community actors and decision-makers to act (R2), leading to actions to address the identified health systems challenges (O).	PT 1: Actively involved	(Butler *et al.*, 2020)
CMOC5: In rural communities (C), participatory communication approaches including sharing personal experiences with peers (R1) allow participants to build problem-solving skills and confidence in a supportive environment (R2). This increased confidence and shared learning improve understanding and knowledge (O1) and participants’ ability to further disseminate knowledge (O2).	PT 1: Actively involved PT 3: Trusted PT 5: Value/benefit	(Morrison *et al.*, 2010, Suchitra *et al.*, 2010) Additional support (Brasington *et al.*, 2016, Gram *et al.*, 2019)
Demi-regularity: role of local leaders		
CMOC6: In communities with existing and trusted local leadership structures (C), when local leaders directly reach out to community members about a programme with appropriately tailored messaging (R1), they are more willing to listen to the information and participate in the programme (O) because they have existing trust and respect for local leaders (R2).	PT 3: Trusted	(Besada *et al.*, 2016, Cofie *et al.*, 2014)
CMOC7: In communities with existing hierarchical social and political structures (C), consulting leaders and involving them in reciprocal communication platforms (R1) allow community members to feel their voices are heard because they trust their leaders (R2). The resulting discussion enables community-wide agreement on project implementation (O).	PT 1: Actively involved PT 3: Trusted PT 4: Reciprocal relationship	(Prata *et al.*, 2012)
CMOC8: In communities with low literacy levels that rely on verbal communication (C), when local leaders serve as the messengers for the programme (R1), community members trust this source of information (R2) and their knowledge of the programme and its messages increase (O).	PT 1: Actively involved PT 3: Trusted	(Hounton *et al.*, 2009)
CMOC9: In communities with existing and trusted local leadership structures (C), engaging local leaders in the development of a programme and its messaging (R1) will influence community members to adhere to the recommendations and increase their demand for health services (O) because they feel a sense of social belonging when they listen to these traditional leaders (R2).	PT 1: Actively involved PT 3: Trusted	(Hounton *et al.*, 2009)
CMOC10: In communities with existing and trusted local leadership structures (C), engaging local leaders early in the development of a programme and involving them in messaging (R1) will improve the relevance of the messaging (O1) and the community members’ acceptance of the programme (O2) because leaders are knowledgeable of the community’s needs (R2) and the community respects and trusts their local leaders (R2).	PT 1: Actively involved PT 2: Acceptable PT 3: Trusted	(Cofie *et al.*, 2014, Hounton *et al.*, 2009, Ntoimo *et al.*, 2021) Additional support (Okonofua *et al.*, 2022)
CMOC11: Where there is limited information in a community (C), involving local trusted leaders to gather contextual information from community members (R1) will improve cooperation and appropriate information (O) as people are trusting of the local leadership (R2).	PT 1: Actively involved PT 3: Trusted	(Marcil *et al.*, 2016)
CMOC12: In communities with existing local leadership structures (C), involving local leaders in the identification of staff/facilitators for a CE programme (R1) encourages trust between the community members and CE staff because of their leaders’ endorsement (R2). This influences their participation in the programme (O).	PT 1: Actively involved PT 3: Trusted	(Ntoimo *et al.*, 2021, Suchitra *et al.*, 2010) Additional support (Okonofua *et al.*, 2022)
CMOC13: In communities with existing local leadership structures and trusted gatekeepers (C), involving trusted local leaders in the programme’s oversight (R1) provides guidance and legitimacy in the eyes of the community (R2), influencing the community’s willingness to engage with the programme (O) because of this perceived legitimacy.	PT 1: Actively involved PT 3: Trusted	(Marcil *et al.*, 2016)
CMOC14: In communities with existing local leadership structures (C), continuously following up with community leaders (R1) will keep the community leaders involved throughout all stages of the programme (O) because they feel a sense of belonging and ownership over the programme (R2).	PT 1: Actively involved	(Ntoimo *et al.*, 2021)
Demi-regularity: role of power and fear		
CMOC15: In communities where problems have been previously neglected (C), a forum with direct interaction between community and government actors (R1) may cause fear of retaliation/reprisal among health providers (R2). This inhibits participation in the communication forum (O).	PT 4: Reciprocal relationship	(Butler *et al.*, 2020)
CMOC16: In communities where health workers are perceived to be in positions of power (C), community members do not want to disrespect this position by providing potentially negative feedback (R2), resulting in a lack of reporting negative feedback on health services (O).	PT 4: Reciprocal relationship	(Hamal *et al.*, 2019) Additional support (Bayley *et al.*, 2015, Scott *et al.*, 2017)
CMOC17: In communities where there is limited health rights awareness (C), communities are not empowered to communicate directly with the health facility staff (R2). This leads to a lack of feedback and accountability from the health services (O).	PT 1: Actively involved	(Hamal *et al.*, 2019) Additional support (Bayley *et al.*, 2015)
CMOC18: In communities where health is managed at the district-level (C), health workers/volunteers serving as a primary contact point for community members (R1) provide an avenue/intermediary group for individuals to communicate concerns they would be unable to share otherwise (O) due to fear of angering their healthcare providers if they were to share these concerns/feedback directly (R2).	PT 4: Reciprocal relationship	(Hamal *et al.*, 2019)
CMOC19: In programmes with public avenues for accountability (i.e. media involvement), using these forums as avenues for actors to follow up on actions/promises (R1) will improve healthcare providers’ accountability to follow through (O) because of their fear of repercussions of public exposure of poor performance (R2).	PT 1: Actively involved	(Hamal *et al.*, 2019)
CMOC20: In communities with marginalized populations (C), social audits/forums for communication (R1) may not achieve significant participation (O) because these participants are unable/reluctant to share their perspectives due to the power imbalance between them and present authorities (R2).	PT 4: Reciprocal relationship	(Hamal *et al.*, 2019)
Demi-regularity: tradition and cultural acceptability		
CMOC21: In rural communities with strong social/cultural cohesion (C), incorporating locally adapted and participatory activities and/or tailored messaging/visual representations (R1) makes the messaging and content more relevant to the group’s needs and frames of reference because they see similarities to their own lives (R2). This will increase the understanding and knowledge of the audience (O).	PT 2: Acceptable	(Besada *et al.*, 2016, Dongre *et al.*, 2009, Prata *et al.*, 2012, Suchitra *et al.*, 2010) Additional support (George *et al.*, 2018, Manandhar *et al.*, 2004, Manandhar *et al.*, 2017, Howard-Grabman, 2007)
CMOC22: In communities where the societal norm/existing decision-making structure is for men to be the head of household/decision-makers (C), having male local champions convey messaging (R1) is more culturally acceptable and these champions are listened to (R2) which leads to community men accepting their messaging (O).	PT 2: Acceptable	(Besada *et al.*, 2016)
CMOC23: In communities with existing local leadership structures (C), observing local traditional practices while reaching out to community leaders (R1) will demonstrate respect to the community leaders (R2) and influence them to be open to the meeting and listen to the programme objectives (O).	PT 2: Acceptable	(Ntoimo *et al.*, 2021)
CMOC24: In communities with strong social/cultural cohesion (C), programmes that are practically organized to respect local practices (e.g. language, logistics and content) (R1) will foster trust between the community and the facilitators (R2) and enable a positive relationship with clear communication between the facilitator and the community (O).	PT 2: Acceptable PT 3: Trusted	(Suchitra *et al.*, 2010)
CMOC25: In communities with informal/traditional care practices (C), retraining these local traditional care providers (R1) will encourage women to utilize the health services (O) because women and their families are comfortable with their usual/existing care providers (such as traditional birth attendants) (R2).	PT 2: Acceptable PT 3: Trusted	(Marcil *et al.*, 2016)
CMOC26: In communities with strong social/cultural cohesion (C), programmes that are practically organized to respect local practices (e.g. language, logistics and content) (R1), positive returns from putting the programme into practice will increase community members’ buy-in (R2) and their trust in the programme (O).	PT 2: Acceptable PT 3: Trusted PT 5: Value/benefit	(Suchitra *et al.*, 2010)
Demi-regularity: building relationships or the role of existing relationships		
CMOC27: In communities with existing communication forums (example: durbars) (C), when community members and health workers interact in these communications forums (R1), they develop a positive relationship with each other where community members feel accepted (R2) and are then more willing to provide honest feedback in future interactions (O).	PT 3: Trusted PT 4: Reciprocal relationship	(Cofie *et al.*, 2014) Additional support (Bayley *et al.*, 2015, Moran *et al.*, 2006)
CMOC28: When there is low uptake in the community of an externally implemented intervention (C), conversations and open dialogue help to establish relationships between the families and the programme (R1), which allows the community members to feel like their feedback is being heard (R2) and increases their service uptake over time (O).	PT 4: Reciprocal relationship	(Marcil *et al.*, 2016)
CMOC29: In health settings with high staff turnover (C), community members do not build a relationship and trust with the healthcare providers (R1) which means they do not feel safe to share their feedback (R2) and there is a lack of open/honest communication between patients/community and providers (O).	PT 4: Reciprocal relationship	(Hamal *et al.*, 2019)
CMOC30: In communities where health is managed at the district level (C), intermediary groups serve as a link between community members and the health sector (R1) and share honest feedback/complaints with healthcare providers (O). This is because community members feel a sense of familiarity and comfort with these intermediary groups that allows them to feel comfortable sharing these concerns/feedback directly (R2).	PT 3: Trusted PT 4: Reciprocal relationship	(Hamal *et al.*, 2019)
CMOC31: When health workers have positive relationships with community members (C) and there is an avenue for community members to provide feedback (R1), community members provide feedback to the health workers (O). This is because they feel their health workers’ value their opinions and their familiarity allows them to feel comfortable to share feedback directly (R2).	PT 4: Reciprocal relationship	(Cofie *et al.*, 2014, Hamal *et al.*, 2019)
Demi-regularity: avenues and forums for communication		
CMOC32: In communities with existing communication structures and forums (C), repeatedly exposing community members to the programme and its logo/symbolism on these channels (R1) will make them familiar with the programme (R2). This recognition will make community members more receptive to the programme (O).	PT 3: Trusted	(Marcil *et al.*, 2016) Additional support (Brasington *et al.*, 2016, Dougherty *et al.*, 2018)
CMOC33: In communities with existing communication forums (C), working with the community to identify and use multiple communication avenues and activities (R1) will increase exposure to messaging and make it more accepted by the community because it is coming from familiar modes of communication (R2). By receiving these messages, community members’ knowledge will increase (O).	PT 3: Trusted	(Cofie *et al.*, 2014, Hounton *et al.*, 2009, Marcil *et al.*, 2016, Prata *et al.*, 2012) Additional support (Dougherty *et al.*, 2018)
CMOC34: In remote, difficult-to-reach communities (C), an interactive component to a radio/virtual messaging forum (R1) allows women to feel they can ask questions that are more relevant and specific to their own concerns (R2), which improves their knowledge about health services (O1) and then increases their care-seeking behaviours (O2).	PT 3: Trusted PT 4: Reciprocal relationship	(Cofie *et al.*, 2014)
CMOC35: In democratic societies with freedom of speech (C), a traditional communication forum with direct interaction and two-way communication between community actors and district officials (R1) provides a safe space where community members feel safe and secure to share their perspectives (R2). This sense of safety and security leads to both sides being willing to participate in the dialogue (O).	PT 3: Trusted PT 4: Reciprocal relationship	(Butler *et al.*, 2020)
CMOC36: In communities where problems have been previously neglected (C), a forum for direct and two-way communication between community actors and district officials (R1) can encourage community members who are enthusiastic to voice their concerns (R2), which will increase district officials’ knowledge on health system issues (O).	PT 4: Reciprocal relationship	(Butler *et al.*, 2020)
CMOC37: In communities with a perceived need for a health programme (C) when they are given a space to provide a programme with feedback and experience the changes based on their feedback (R1), this will establish a feedback loop and they will be more accepting of the programme (O) because they feel valued and heard (R2).	PT 4: Reciprocal relationship	(Ntoimo *et al.*, 2021)
Demi-regularity: peers as models or connections		
CMOC38: In communities where health services are underutilized and the community participates in a programme (C), having local champions or individuals influential in community networks share the messaging and experiences (R1) will encourage their peers/neighbours to participate because they trust these individuals (R2). This will enable sustainable increases in utilization beyond the project itself because the local influencers are serving as champions to encourage uptake (O).	PT 3: Trusted PT 5: Value/benefit	(Marcil *et al.*, 2016, Ntoimo *et al.*, 2021) Additional support (Dougherty *et al.*, 2018)
CMOC39: In communities where the societal norm is for men to be the head of household/decision-makers (C), having ‘model male’ local champions convey messaging (R1) increases community men’s acceptance of messaging (O) because these champions model ‘ideal’ behaviour that other men want to emulate (R2)	PT 2: Acceptable PT 3: Trusted	(Besada *et al.*, 2016)
CMOC40: In rural communities with often marginalized and vulnerable populations (C), organized community group members can advocate for various community members to attend meetings (R1). This inclusivity means more community members become familiar with and are willing to engage with the programme (R2), which increases the overall community’s awareness of health issues (O).	PT 3: Trusted	(Suchitra *et al.*, 2010)
Demi-regularity: programme or service provision		
CMOC41: In communities where problems have been previously neglected (C), community members sharing personal experiences and stories (R1) that have not been acted upon leads to frustration (R2). This inhibits community members from participating in or contributing to communication forums (O).	PT 1: Actively involved PT 5: Value/benefit	(Butler *et al.*, 2020)
CMOC42: In communities suspicious of externally implemented interventions due to previous experience with such programmes (C), providing services (R1) may help to build trust with the community by introducing them to the initial benefits from the programme (R2). As the community experiences these benefits and trusts the programme, they begin to participate and support it (O).	PT 3: Trusted PT 5: Value/benefit	(Marcil *et al.*, 2016)
CMOC43: In communities suspicious of externally implemented interventions due to previous experience with such programmes (C), providing services (R1) may make the community sceptical of the programme as they feel they are being imposed on (R2). This will make the community less likely to accept and participate in the programme (O).	PT 5: Value/benefit	(Marcil *et al.*, 2016) + expert advisory committee
CMOC44: In a community that lacks health services/access (C), when the community benefits from the programme and its messaging (R1), it will increase the community’s buy-in to the programme (R2) and the community will therefore accept the programme (O) as they see more positive returns and appreciate its value.	PT 5: Value/benefit	(Ntoimo *et al.*, 2021) Additional support (Howard-Grabman, 2007)
CMOC45: In communities where a social audit/CE programme has been implemented (C) and led to healthcare providers being perceived as increasingly concerned for women/their patients (R1), women/patients trust the health services due to this concern (R2), leading to the increased use of maternal health services (O).	PT 3: Trusted PT 5: Value/benefit	(Hamal *et al.*, 2019)

The main source provided in the table is the reference(s) of ‘high’ or ‘moderate’ richness that provided data to inform the development of the CMOC. The additional support sources are the ‘low’ richness papers that, while they did not provide enough data or information to configure an entire CMOC statement, did provide some evidence that could be inferred to support the CMOC. Feedback from the expert advisory committee fed into the CMOCs but is only explicitly noted when substantial revisions were made due to their involvement.

**Figure 3.**
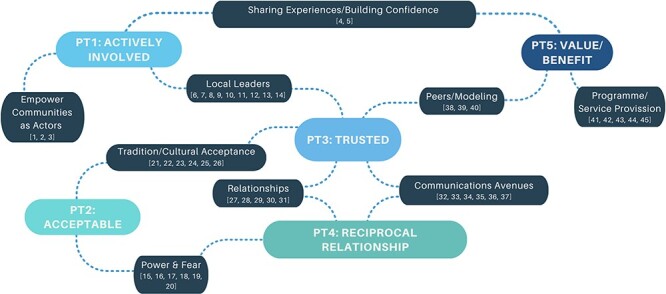
Demi-regularities informing PTs and how they map to three of the stages of the C4D framework: the nine demi-regularities with their corresponding CMOCs (in which CMOCs are denoted in parentheses) fed into the five PTs that occur across the C4D framework

**Figure 4.**
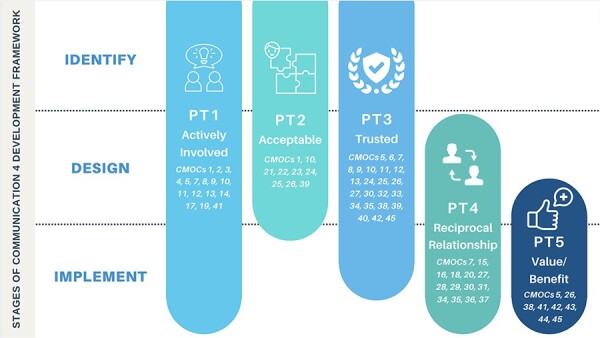
Programme theories (PT) and Communications 4 Development Framework stages

These 45 CMOCs were organized into nine demi-regularities informing the refinement and development of five PTs (Figure 3). Based on the data, five new data-led PTs were mapped to the stages of the C4D framework (Figures 3 and 4) identified by the IPTs in Phase 1 (Supplementary File 7), the realist review protocol. Box 1 exhibits the five refined PTs put forward by this review.

### PT 1: Community is actively involved (co-creation)

The first PT is supported by 16 CMOCs extracted from nine documents (Hounton *et al.*, 2009; Morrison *et al.*, 2010; Rath *et al.*, 2010; Prata *et al.*, 2012; Cofie *et al.*, 2014; Marcil *et al.*, 2016; Hamal *et al.*, 2019; Butler *et al.*, 2020; Ntoimo *et al.*, 2021) (see Table 2), with supporting evidence from eight additional documents (Bayley *et al.*, 2015; Brasington *et al.*, 2016; George *et al.*, 2018; Mochache *et al.*, 2018; Gram *et al.*, 2019; Demissie *et al.*, 2020; Ekirapa-kiracho *et al.*, 2020; Okonofua *et al.*, 2022). This PT explains the involvement of the community as co-creators throughout the process of developing and implementing communications strategies in CE for MNH.

This PT describes how consulting communities and having them own and conduct the communications in the CE can contribute to improving both communities’ knowledge and buy-in of a programme overall. This can look like involving local leadership structures in the recruitment of staff and building off of existing avenues for accountability and communication within communities. As described by Hounton *et al.* (2009), ‘Although there is no magic bullet, nor a one-size-fits-all ideal community mobilisation approach, closely working with communities by consulting them at all stages of design, planning and implementation of delivery care is critical for achieving reduction in maternal and perinatal mortality.’ This example from the literature also points to how this PT cuts across the stages of the C4D framework—emphasizing the involvement of the community throughout the process of identifying, designing and implementing communication.

### PT 2: Messaging and programme are acceptable

Nine CMOCs extracted from eight documents (Dongre *et al.*, 2009; Hounton *et al.*, 2009; Rath *et al.*, 2010; Prata *et al.*, 2012; Cofie *et al.*, 2014; Besada *et al.*, 2016; Marcil *et al.*, 2016; Ntoimo *et al.*, 2021) and further supported by eight documents provide evidence for PT 2 (Howard-grabman, 2007; Brasington *et al.*, 2016; Manandhar *et al.*, 2004; George *et al.*, 2018; Mochache *et al.*, 2018; Demissie *et al.*, 2020; Ekirapa-kiracho *et al.*, 2020; Okonofua *et al.*, 2022). This PT describes the importance of messaging (the information, content or material that is being verbalized, written, shared or discussed) that is adapted and tailored to the community context.

Similar to PT1, this PT may require the involvement of community members and their perspectives in order to identify the most appropriate and acceptable messaging content and mediums. As described in the literature, ‘the production and iterative adaptation of locally appropriate picture cards, stories, and participatory games increased acceptability and catalysed learning and planning within the groups’ (Rath *et al.*, 2010). Communications should be shared via ‘culturally acceptable activities’ (Hounton *et al.*, 2009) that are built on existing programmes and avenues while including ‘locally relevant cultural dynamics’ (Besada *et al.*, 2016) in the messaging that are relatable to the audience. This can be done by incorporating local norms and expectations in verbal messaging, as well as using local visual similes:

When community members learned that 500 ml of blood loss was the threshold for postpartum haemorrhage, they identified the moda, a local rubber cup used for fetching water from the water pot, which holds exactly 500 ml of water, as a useful volume reference. This provided clear visual representations for the community to understand when a woman had lost too much blood after delivery and was facing a life-threatening emergency. (Prata *et al.*, 2012)

These visual examples and more were identified or recommended by community members, providing insight that might not be feasible from an external perspective, further supporting the active role of communities identified in PT 1. Additionally, testing messaging content and approaches with the target audience to ‘test if the pictures and messages conveyed the appropriate meaning’ (Dongre *et al.*, 2009) can provide an opportunity to adjust communications as per the audience’s needs and contexts. As a result, this PT is applicable throughout the stages of the C4D framework, from the initial identification of messaging and priorities to the design and actual implementation of how communication is shared.

### PT 3: Communication sources are trusted

The third PT is supported by the 23 CMOCs extracted from 10 documents (Hounton *et al.*, 2009; Morrison *et al.*, 2010; Rath *et al.*, 2010; Prata *et al.*, 2012; Cofie *et al.*, 2014; Besada *et al.*, 2016; Marcil *et al.*, 2016; Hamal *et al.*, 2019; Butler *et al.*, 2020; Ntoimo *et al.*, 2021), with further evidence supporting these CMOCs found in six additional documents (Moran *et al.*, 2006; Bayley *et al.*, 2015; Brasington *et al.*, 2016; Dougherty *et al.*, 2018; Gram *et al.*, 2019; Okonofua *et al.*, 2022). This PT emphasizes the role of trust in communications for CE in MNH programming.

The strategies or forums through which communication is delivered to communities must be seen as credible and trustworthy. This may include messengers and avenues of communication that are familiar to community members, potentially building off of existing trust. Ntoimo *et al.* (2021) explained that having community leaders recruit the Ward Development Committee members ‘enabled the selection of the most trusted and reliable persons in the community to guide the process’. Furthermore, Rath *et al.* (2010) highlighted that facilitators built trust with the community ‘by being from the study area, respecting local practices, and knowing local languages’. Meanwhile, Marcil *et al.* (2016) reported that women were ‘more comfortable utilizing its services when [birth attendants] with whom they are already familiar provide care’. Hamal *et al.* (2019) described how ‘high staff turnover prevents women from building a relationship of trust with healthcare workers’, pointing to the importance of relationships in developing this trust. As highlighted by this PT, the role of trust is integral throughout the stages of the C4D framework. Building this trust may start as early as the initial identification stage and continues to be important throughout the roll-out and implementation of the communications.

### PT 4: Community has a reciprocal relationship with the programme

The fourth PT is supported by 14 CMOCs that were extracted from six documents (Prata *et al.*, 2012; Cofie *et al.*, 2014; Marcil *et al.*, 2016; Hamal *et al.*, 2019; Butler *et al.*, 2020; Ntoimo *et al.*, 2021) and were further supported by three additional documents (Moran *et al.*, 2006; Bayley *et al.*, 2015; Scott *et al.*, 2017). This PT explores how relationships between the community, or the specific audience of the communications approach, and actors involved with the programme can influence how the community interacts with the programme.

Box 1. Refined PTs from Phase 2

**PT 1: Community is actively involved (co-creation)**

When communities are actively involved throughout the identification, design and implementation of communication messaging and strategies for an MNH community engagement programme, the communication is more relevant, acceptable and trusted. Community members play an active role in co-creating the communication messaging by informing what information should be communicated (e.g. community’s priorities and needs) and how (e.g. communication methods and avenues), identifying challenges or misunderstandings, holding programme implementers accountable and raising overall awareness of the programme through tailored messaging. This increases their ownership over the MNH programme, enabling longer-term sustainability.

**PT 2: Messaging and programme are acceptable**

When MNH implementers acknowledge and consider local practices/norms and power structures in the communication messaging/processes, and the communication approaches and programme goals are tailored appropriately to the community’s needs, norms and expectations, then the MNH programme and the messaging and MNH programme are likely to be acceptable to and shared further by the community.

**PT 3: Communication sources are trusted**

When messaging is aligned with community members’ values and experiences and is delivered through familiar or agreed upon communication avenues/structures by respected and influential messengers and the programme is also perceived to have positively contribute to the community, then the communication sources for the community engagement programme are trusted.

**PT 4: Community has a reciprocal relationship with the programme**

When the actors involved in an MNH programme (including local leaders, stakeholders, implementers and health providers) develop a positive relationship with community members and directly act on feedback from the community to inform the programme and messaging, community members feel heard and valued as equals in a reciprocal relationship with the MNH programme.

**PT 5: Community sees value or benefit from the programme**

When a community experiences/perceives value or benefits from an MNH programme through the messaging shared, knowledge gained or services provided, then they are inclined to continue to support/participate in the programme and disseminate messaging further. This enables the longer-term sustainability or continued functioning of the programme, beyond an ‘intervention period’.

This PT highlights the reciprocal nature of a relationship—emphasizing that communications and dialogue are a two-way street rather than a one-sided or top-down experience. Examples from the literature highlighted the importance of a ‘cyclical process of feedback’ (Marcil *et al.*, 2016) and how conversations between community members and other groups, such as health workers, enabled the development of positive relationships between the two (Cofie *et al.*, 2014; Marcil *et al.*, 2016). Others described how the context of a community, including levels of free speech and a community’s awareness of health rights, can influence the development of these relationships and the openness of conversations community members feel they can have and their willingness to provide honest feedback (Hamal *et al.*, 2019; Butler *et al.*, 2020). While the role of relationships is relevant throughout the C4D stages, this PT can be most relevant in the design and implementation of communications approaches by incorporating processes to respond and act upon community feedback.

### PT 5: Community sees value or benefit in the programme

Eight CMOCs from six documents (Morrison *et al.*, 2010; Rath *et al.*, 2010; Marcil *et al.*, 2016; Hamal *et al.*, 2019; Butler *et al.*, 2020; Ntoimo *et al.*, 2021) and supported by an additional three documents provide evidence for the fifth PT (Howard-grabman, 2007; Brasington *et al.*, 2016; Gram *et al.*, 2019). This PT describes how communities may respond or continue to interact with a programme when they experience or perceive potential values or benefits based on the messaging or service provision they have received.

Examples from the literature (Marcil *et al.*, 2016; Ntoimo *et al.*, 2021) explained how communities’ previous experiences with pilot programmes affected their trust in an intervention, calling for the programme to demonstrate they were serious by rolling out service provision early: ‘once the community members experienced benefits from the program, they became more willing to engage’ (Marcil *et al.*, 2016). This also applied to the information community members received through communications messaging. As described by a facilitator in Rath *et al.*’s study: ‘Group members believe our words and the contents discussed during the meetings. They implement them and when they get the benefits their trust strengthens’ (Rath *et al.*, 2010). This direct experience of benefitting from the received communication can become an important component for the further sustainability of a program’s messaging by motivating community members ‘to disseminate information about maternal and neonatal health to other women’ (Morrison *et al.*, 2010). This PT can be most clearly applied when the messaging and subsequent programming are introduced to the community through the design and implementation phases of the C4D framework.

## Discussion

The findings of this realist review highlight the importance of centring the community’s needs and priorities throughout the stages of developing and implementing communications for CE through the active involvement of the community in a programme, the acceptability of messaging, the trustworthiness of how this messaging is delivered, the positive and reciprocal relationship between the community and the programme and the perception of value/benefits from the programme. This realist review used a narrowed scope to focus on CE communications for MNH. While the PTs presented were developed from examples of programming within the field of MNH, they may have wider applications. This might be because the PTs describe the processes or how communications in CE are functioning, which is perhaps less influenced by what health area specifically the activities aim to address. The realist review is theory-driven and data-led, and so the PTs presented in this realist review differ from the IPTs described in the protocol because substantial evidence provided a different direction for the theories. However, elements of the IPTs still exist across the newly developed theories and can still be traced across the C4D stages (as demonstrated in Figure 3). The C4D stages were used to provide an organizational framework that could demonstrate the applicability of the PTs by highlighting at which stages they could be incorporated or considered. This process also highlighted that the PTs may span across the C4D stages, rather than being confined to chronological stages. This is an important consideration that reflects the emphasis on centring the community in CE communication.

The PTs developed through this realist review mirror previous experiences from the field and substantive theories in the literature. For example, when considering the design of acceptable messaging and programming (*PT 2*), the Theory of Planned Behaviour (Ajzen, 1985; 2011) also supports the role of local collaboration in communication messages to influence behavioral beliefs (how someone perceives the outcome of a behavior) by improving the messaging’s acceptability because it is more contextually relevant and appropriate (Dada *et al.*, 2021b). Similarly, Fisher’s Narrative Theory describes how hearing stories that are relevant and contextualized in shared experiences makes individuals more likely to believe and accept given advice, influencing behavior change, because the examples provided ‘ring true’ and fit in an individual’s frame of reference (Fisher, 1987; Edgar and Volkman, 2012). Storytelling, in both individual and group settings, is an important practice in many parts of the world and has even been used as a tool for communication in previous MNH interventions in sub-Saharan Africa where there is a rich oral tradition in the region (Silver, 2001; Carter-Black, 2007; Cancel and Turin, 2013; Mushibwe *et al.*, 2021). This is demonstrated in the radio and theatre approaches taken by the programmes included in this review (Cofie *et al.*, 2014; Besada *et al.*, 2016). Pratt and Boyden (1985) take this a step further in explaining that not only should information shared be relevant but this exchange of similar experiences amongst groups can encourage action (Aruma, 2018).

The roles of trust and reciprocal relationships (‘PT 3 and PT 4’) have also been previously recognized as integral components to CE activities conducted for research, often epitomized in the design of a study’s objectives or by the informed consent process or seen in activities such as ‘community advisory boards’ for biomedical research (Marsh *et al.*, 2010; Dada *et al.*, 2019; Vincent *et al.*, 2022). Previous literature posits that trust in interpersonal communication involves perceiving the speaker as knowledgeable and competent (Pearce, 1974). Leaders may then be a common go-to messenger because they have this established relationship with the community and theories around effective leadership emphasize the role of trustworthiness in this dynamic (Zeffane, 2010). Similarly, substantive theory around message framing has described how emphasizing the benefits of a programme or behavior increases the likelihood of uptake of the desired behavior change, especially when this messaging comes from leaders (Hendrix *et al.*, 2014; Dada *et al.*, 2021a). Paoulo Freire’s work on participatory communication sets the stage for centring communication as a dialogue between various groups and stakeholders (Servaes and Malikhao, 2005). A reciprocal relationship in dialogue means both parties are engaged and involved in questioning, interpreting and shaping the messaging (Burbules and Bruce, 2001). This two-way communication provides an opportunity for collaborative approaches across equal parties (Ansell and Gash, 2008; Emerson *et al.*, 2012; Farid and Li, 2021). Acting upon the community’s comments through such feedback loops has been used to demonstrate that the programme implementers are listening to the community and that they are valued as co-creators in a programme (Jackson *et al.*, 2018; Dada *et al.*, 2019).

At the collective or community level, several of the included documents in the review describe the concept of a ‘critical consciousness’ and sense of belonging that are developed when the community is actively involved in co-creating the CE communications (‘PT 1’) to identify and respond to challenges and how this can further enable the sustainability of a programme (Morrison *et al.*, 2010; Rath *et al.*, 2010; George *et al.*, 2018; Mochache *et al.*, 2018; Ekirapa-kiracho *et al.*, 2020). First proposed by Freire (1974), critical consciousness refers to when a community (often a marginalized group) becomes aware of and takes action against systems that perpetuate, in this case, poor maternal health (Rath *et al.*, 2010; Diemer *et al.*, 2016). This critical consciousness could be both an outcome of a community’s early and systemic involvement in messaging development and a mechanism that propagates the sustainability of the programme. The discussions around critical consciousness and the implications of the review findings for CE communications at the group or community level also point to an idea that is not explicitly captured by the current PTs: CE communications as a unifying force. Building off of the work of others, Aruma (2018) describes communication as ‘a unifier of community development activities in various communities in the society’, which may be likened to CE. The ways in which CE communication unifies communities and MNH programmes may provide an additional direction for future research to consider.

**Figure 5.**
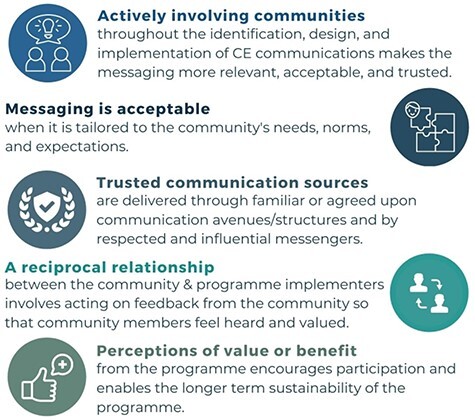
Translating PTs into practice: the five PTs presented as key takeaways and recommendations for practice

Realist reviews provide opportunities for transferable findings because of the insight they provide into the contexts and mechanisms that interact to produce certain outcomes (Boyko *et al.*, 2018). As a result, the findings of this review can have implications for policy, practice and research. Better insight and an understanding of what and how CE communications work, for whom and why will inform meaningful recommendations for policy and practice. CE is a complex intervention that operates within the complexity of health systems, requiring the design and implementation of policies to consider this complexity (DE Savigny and Adam, 2009). The findings of this review demonstrate the layered and complex nature of CE communications through the interconnected nature of the PTs. For example, co-creating with communities early described by ‘PT 1’ influences the acceptability of messaging (‘PT 2’) as well as the trust in communications (‘PT 3’) and the overall relationship with the programme (‘PT 4’). Figure 5 provides the PTs as pragmatic takeaways and recommendations for practice, demonstrating how they can inform the design of future programmes.

In terms of practice, the refined theories align with existing guidance on communication in CE. For example, it is known that CE should not be tokenistic and must meaningfully engage populations and communities (Johnston, 2010; Parker and Murray, 2012; Hahn *et al.*, 2017; Vincent *et al.*, 2022). Findings from this review describe how and why communication works to support meaningful engagement. This can be used to design more appropriate CE communication strategies for different MNH programmes. As demonstrated by ‘PT 5’, communities must perceive some value or benefit from the CE communications (through messaging content or programme processes) in order to sustain the program’s success. Examples from the literature describe how acting on community feedback elicited through CE activities to adapt interventions can exhibit this (Enria *et al.*, 2016; Dada *et al.*, 2019). While previous reviews have highlighted the need for additional and context-specific evidence relating to the implementation of CE processes (Dada *et al.*, 2021b; Vincent *et al.*, 2022), it is also useful to note that research from other fields can contribute to the development and adaptation of CE communications for MNH. For example, principles for effective communication in general can inform the construction of CE messaging that is accessible, actionable, credible and trusted, relevant, timely and understandable (WHO, 2017b).

While these PTs present what may enable effective CE communications for MNH programming, the wider fields of C4D and social behavior change communication have described a number of challenges that may also be applicable to MNH. For example, challenges include budget and resource constraints, the ability to account for the shifting nature of communities’ priorities and relationships, navigating local and global partnerships, developing scalable and sustainable programmes, developing trust and confidence between individuals and the capacity to monitor and evaluate communications campaigns (Button and Rossera, 1990; Waisbord, 2005; Servaes and Lie, 2015; Turk *et al.*, 2022). Some of these challenges have been addressed by prioritizing budget allocations to communications, pre-testing messages and using a range and number of communication channels where possible (Bayih *et al.*, 2021; Kaur, 2022). Relatedly, the timing of CE should not be overlooked and is emphasized throughout the PTs: incorporating and involving the community from the inception of a programme is vital (Marsh *et al.*, 2008), with this review highlighting the importance of their continuous and meaningful input in influencing the acceptability, trustworthiness and perceived value of the programme. Similarly, principles of co-design emphasize the involvement of stakeholders or end users in the design of sustainable and appropriate interventions (Till *et al.*, 2022). Co-design has been used for MNH programming to develop messaging content and avenues identified by the target audience and to improve service delivery (Kynoch *et al.*, 2022; Perkes *et al.*, 2022). Programme implementers designing CE could employ co-design through workshops and other participatory approaches in the development of more appropriate communications (Sanders and Stappers, 2008).

### Strengths and limitations

This realist review has several strengths and limitations. Conducted according to RAMESES guidelines (Wong *et al.*, 2013a), this realist review provides a transparent record of the processes undergone and decisions made. All information relevant to the review and decision-making processes is provided clearly in the text or in the supplementary materials. This level of detail adds value to the broader literature base of realist reviews by describing the specific steps taken in applying the methodology. Most notably as an example, few published realist reviews provide concrete explanations on how ‘richness’ and sometimes ‘rigor’ are determined or assessed, whereas this realist review provides these guidelines more explicitly. Finally, consulting experts or stakeholders is a commonly recommended component of realist reviews (Wong *et al.*, 2013a; 2013b). This realist review meaningfully engaged a group of experts with a diverse range of backgrounds and expertise and incorporated their feedback in multiple steps throughout the realist review. Details relating to how and to what extent the expert advisory committee was engaged have also been included in this article.

One of the limitations of this realist review is the nature of what literature is available and accessible. While there is a great deal of work being done relating to CE for MNH, it may not always be recorded or published publicly. This lack of descriptions detailing what CE communications were conducted or the processes involved made it challenging to answer the ‘for whom’ CE communications work, as elements of context were particularly difficult to extract. Future research, such as a realist evaluation, can work to further unpack this. Another limitation of this review may have been the broad search strategy that was used by starting with the records from a previously conducted scoping review. While the scoping review was broad, this meant the initial search strategy for this realist review was not specific to ‘communications’. However, the iterative searches that are an important component of realist reviews allowed for reviewers to go back and search for additional data and evidence from a range of sources where necessary. Finally, a limitation of this review is in the application of the rigor assessment. Due to the range of guidelines pertaining to assessing rigor in a realist review, this was conducted in a number of ways—first through standardized assessment tools and then through a more subjective assessment of the evidence based on the plausibility and coherence of the CMOCs it informed. However, we have accounted for this by providing a list of some of the guiding questions that were considered and how the outcomes of these rigor assessments influenced the review.

## Conclusion

Engaging the community is important in enabling cost-effective and sustained behavior change that improves health outcomes through informing the development of contextually appropriate interventions and empowering populations (Farnsworth *et al.*, 2014; Haldane *et al.*, 2019). This realist review explores how, why, for whom and to what extent communications for CE work in MNH programming through the presentation of five PTs (Head, 2007; Adhikari *et al.*, 2020). The significant contribution of peer-reviewed literature to this review highlights the importance of transparent and detailed reporting of what and how CE communications are developed and implemented.

Most notably, the purpose of research such as this that aims to provide a better understanding of what works for CE communications is to be able to use the findings to support local organizations and countries to develop and implement CE in practice. This is a broad realist review and may need further refinement based on the outcome or specific types of CE activities and their communication components in order to be more specific. Additionally, future research may consider exploring and categorizing the types of outcomes produced. As a next step, these theories will be further tested and refined through a realist evaluation case study. Future primary research that tests these theories across settings (including both geographical settings but also within different health and social programmes) and over time can provide a more in-depth understanding of generative causation, which can be used to support the design and implementation of future CE communications across contexts.

## Supplementary Material

czad078_Supp

## Data Availability

All data analysed in this study are included in the publication’s supplementary material.

## Reflexivity statement

The authors include five women and three men spanning multiple levels of seniority and from different sectors. The authors represent six different nationalities, with three based in institutions in LMICs. Two of the authors are realist methodology experts. Four authors specialize in a range of global health programming and research across India, Kenya and Zambia, while the other two authors also have experience conducting qualitative fieldwork in sub-Saharan Africa.
