# Supplementary material for: Understanding communication in community engagement for maternal and newborn health programmes in low- and middle-income countries: a realist review
Source: Health Policy Plan. 2023 Aug 31;38(9):1079–98. doi: 10.1093/heapol/czad078 (PMC10566325; doi:10.1093/heapol/czad078)
Supplement: czad078_Supp [file czad078_supp.zip › suppl_data/RR Supp Files_rev1.docx]

Supplementary Files

Table of Contents

[Supplementary File 1: RAMESES Publication Standards for Realist Syntheses – Adapted Checklist 2](#_Toc140051939)

[Supplementary File 2: Search strategy 5](#_Toc140051940)

[Supplementary File 3: Data extraction 6](#_Toc140051941)

[Supplementary File 4: Expert advisory committee guided feedback form for CMOCs 59](#_Toc140051942)

[Supplementary File 5: Low paper data extraction/confirmation 60](#_Toc140051943)

[Supplementary File 6: CMOC organisation process 63](#_Toc140051944)

[Supplementary File 7: Initial programme theories identified in Phase 1 of the review (realist review protocol) 64](#_Toc140051945)

[Supplementary File 8: Expert advisory committee feedback form for PTs 65](#_Toc140051946)

[Supplementary File 9: Description of CE communications in the 11 extracted studies 72](#_Toc140051947)

# Supplementary File 1: RAMESES Publication Standards for Realist Syntheses – Adapted Checklist

The following table/checklist provides a replication of **Table 1** from Wong, G., Greenhalgh, T., Westhorp, G. *et al.* RAMESES publication standards: realist syntheses. *BMC Med* **11**, 21 (2013). <https://doi.org/10.1186/1741-7015-11-21> along with an additional column identifying where the requirement is being met in the manuscript.

| **SECTION** | | | **Page # in Manuscript** |
| --- | --- | --- | --- |
| **TITLE** | | | |
| 1 | In the title, identify the document as a realist synthesis or review | | *Title page* |
| **ABSTRACT** | | |  |
| 2 | While acknowledging publication requirements and house style, abstracts should ideally contain brief details of: the study's background, review question or objectives; search strategy; methods of selection, appraisal, analysis and synthesis of sources; main results; and implications for practice. | | *page 1* |
| **INTRODUCTION** | | |  |
| 3 | Rationale for review | Explain why the review is needed and what it is likely to contribute to existing understanding of the topic area. | page 3 - Background |
| 4 | Objectives and focus of review | State the objective(s) of the review and/or the review question(s). Define and provide a rationale for the focus of the review. | page 3 - Background |
| **METHODS** | | |  |
| 5 | Changes in the review process | Any changes made to the review process that was initially planned should be briefly described and justified. | N/A |
| 6 | Rationale for using realist synthesis | Explain why realist synthesis was considered the most appropriate method to use. | page 3 - Background |
| 7 | Scoping the literature | Describe and justify the initial process of exploratory scoping of the literature. | page 4 – Methods + described in protocol |
| 8 | Searching processes | While considering specific requirements of the journal or other publication outlet, state and provide a rationale for how the iterative searching was done. Provide details on all the sources accessed for information in the review. Where searching in electronic databases has taken place, the details should include, for example, name of database, search terms, dates of coverage and date last searched. If individuals familiar with the relevant literature and/or topic area were contacted, indicate how they were identified and selected. | page 4 – Search for evidence |
| 9 | Selection and appraisal of documents | Explain how judgements were made about including and excluding data from documents, and justify these. | page 4 – selection & appraisal of evidence |
| 10 | Data extraction | Describe and explain which data or information were extracted from the included documents and justify this selection. | page 4 – data extraction |
| 11 | Analysis and synthesis processes | Describe the analysis and synthesis processes in detail. This section should include information on the constructs analyzed and describe the analytic process. | page 5 – evidence synthesis |
| RESULTS | | |  |
| 12 | Document flow diagram | Provide details on the number of documents assessed for eligibility and included in the review with reasons for exclusion at each stage as well as an indication of their source of origin (for example, from searching databases, reference lists and so on). You may consider using the example templates (which are likely to need modification to suit the data) that are provided. | figure 2 |
| 13 | Document characteristics | Provide information on the characteristics of the documents included in the review. | page 6 – results |
| 14 | Main findings | Present the key findings with a specific focus on theory building and testing. | pages 6-9 – results |
| DISCUSSION | | |  |
| 15 | Summary of findings | Summarize the main findings, taking into account the review's objective(s), research question(s), focus and intended audience(s). | pages 9-11 – discussion |
| 16 | Strengths, limitations and future research directions | Discuss both the strengths of the review and its limitations. These should include (but need not be restricted to) (a) consideration of all the steps in the review process and (b) comment on the overall strength of evidence supporting the explanatory insights which emerged.  The limitations identified may point to areas where further work is needed. | page 11 - strengths & limitations |
| 17 | Comparison with existing literature | Where applicable, compare and contrast the review's findings with the existing literature (for example, other reviews) on the same topic. | pages 9-11 – discussion |
| 18 | Conclusion and recommendations | List the main implications of the findings and place these in the context of other relevant literature. If appropriate, offer recommendations for policy and practice. | page 12 – conclusion |
| 19 | Funding | Provide details of funding source (if any) for the review, the role played by the funder (if any) and any conflicts of interests of the reviewers. | Title page |

# Supplementary File 2: Search strategy

| 1 | RMNCH (title/abstract) | antenatal OR prenatal OR pregnan* OR matern* OR “child health” OR “newborn health” OR postpartum OR postnatal OR perinatal OR reproductive OR birth OR “family plan*” OR neonat* OR ANC OR PNC OR MNCH OR RMNCH |
| --- | --- | --- |
| 2 | ‘Community Blank’ terms (all fields) | “citizen participation” OR “citizen engagement” OR “collaborative partnership” OR “community action” OR “community advisory” OR “community consultation” OR “community collaboration” OR “community engagement” OR “community involvement” OR “community mobilization” OR “community mobilisation” OR “community liaison” OR “community network*” OR “community participation” OR “grassroots participation” OR “grassroots network*” OR “public engagement” OR “public participation” OR “public representation” OR “participatory action” OR “participatory learning” OR “stakeholder engagement” OR “social engagement” OR “social accountability” |

# Supplementary File 3: Data extraction

| **Author** | **Year** | **Publication Type** | | **Aims/Objectives** | **Setting** |
| --- | --- | --- | --- | --- | --- |
| [Cofie et al.](https://www.ncbi.nlm.nih.gov/pmc/articles/PMC4310571/) | 2015 | Peer-Review Article | | Identifying community outreach approaches, describing how quality improvement (QI) teams strengthened community-based initiatives, and identifying challenges to engagement with communities | Ghana |
| **Study Participants** | **Study Design** | **Findings** | | **Description of CE Communications Activities & Who they target** | **Models/Theoretical Frameworks** |
| QI team members (nurses, midwives, health facility observations) | Qualitative case study | Two outreach approaches used (community-level health promo and education + one-to-one activities). Specific barriers to each type of outreach and in different settings. Highlights importance of context-specific activities. | | community radio broadcasts, community engagement and education at public gatherings (durbars), home visits to pregnant women and mothers | N/A |
| **What is interesting about this paper?** | | **Richness Rating** | **Rigour Rating** | **Reference List/Snowballing** | |
| Paper describes various aspects of the community outreach and how it was conducted in the community (most notably the involvement of local leaders, relationships with community members, and some of the challenges/barriers faced by the community outreach activities) | | High | Medium | Björkman M, Svensson J. Power to the people: evidence from a randomized field experiment on community-based monitoring in Uganda. *TheQuarterly Journal of Economics.*2009;124(2):735–769  Singh K, Speizer I, Handa S, Boadu RO, Atinbire S, Barker PM, Twum-Danso NA. Impact evaluation of a quality improvement intervention on maternal and child health outcomes in Northern Ghana: early assessment of a national scale-up project. *International journal for quality in health care.*2013;25(5):477–487.  Blackwell AG, Colmenar R. Community-building: from local wisdom to public policy. *Public Health Reports.*2000;115(2–3):161–166. | |

| **CMOC1** | **Context** | **Mechanism** | **Outcome** | **Additional Information & Excerpts from Text** |
| --- | --- | --- | --- | --- |
|  | Existing local leadership structures and gatekeepers who are influential and trusted in the community | ***Resource***: Programme engaged leaders early to identify relevant priorities and messaging  ***Reasoning***: Programme perceived as relevant and credible because of leader buy-in | Programme priorities (messages at gatherings) are relevant to community’s needs. | QI teams began the process of community-level outreach by first seeking consent, advice, and logistical assistance from community gatekeepers, including chiefs, elders, and religious leaders: *We called our chiefs, we sat down with them and then we asked them to choose some particular community that was not coming early to register.* |
|  | *When a programme engages local leaders early to identify community priorities for messaging (R1) in communities with existing and trusted local leadership structures (C), the programme messaging is credible and improved due to the leader buy-in (R2), which influences the credibility and relevance of the programme messaging content to the community needs (O).* | | | |
| **Relevant IPT(s)** | ***Notes**** | | | |
| IPT1 | *Early involvement of community leaders identified appropriate* ***avenues/forums + priority areas for messaging/program****, making the program relevant to community.* | | | |
| **CMOC2** | **Context** | **Mechanism** | **Outcome** | **Additional Information & Excerpts from Text** |
|  | Existing local leadership structures and gatekeepers who are influential and trusted in the community | ***Resource:*** Leaders invite community members to gatherings  ***Reasoning:*** Community members have existing trust and respect for local leaders which influences their decision to accept invitation | Community attends community gatherings. | Community leaders were actively engaged in identifying appropriate venues for gatherings and inviting community members. |
|  | *When a local leaders reach out directly to community members to invite them to participate (R1) in communities with existing and trusted local leadership structures (C), community members are more likely to accept their invitation and participate in the programme (O) because they have existing trust and respect for local leaders (R2).* | | | |
| **Relevant IPT(s)** | ***Notes**** | | | |
| IPT 3 |  | | | |
| **CMOC3** | **Context** | **Mechanism** | **Outcome** | **Additional Information & Excerpts from Text** |
|  | Community has existing ‘town hall’ avenues (e.g - durbas) where they regularly gather for various assemblies and activities. | ***Resource***: Public interactions between community members and health workers  ***Reasoning***:  Positive relationship/ rapport between community members and health workers where community members feel accepted/valued | Community provides honest feedback on their perspectives at durbars | As noted above, *durbars* became venues for community members to provide feedback and to offer their perspectives on the efforts to promote maternal and child health. **Public interactions between community members and health workers also created rapport** between the two groups. Participants indicated that community members felt their opinions were valued because they were **allowed to express their views,** which encouraged **active participation and dialogue**. |
|  | *In communities with existing communications structures (C), when community members and health workers interact (R1) they develop a positive relationship between each other where community members feel accepted (R2) and are then more willing to provide honest feedback in future interactions (O).* | | | |
| **Relevant IPT(s)** | ***Notes**** | | | |
| IPT2/IPT3 | *Public interactions enable relationship/ rapport between the community members and health workers. Community members feel their opinions are valued* | | | |
| **CMOC4** | **Context** | **Mechanism** | **Outcome** | **Additional Information & Excerpts from Text** |
|  | Established relationship between community members and health workers | ***Resource***: Opportunities to ask/look for community feedback at durbars  ***Reasoning***:  Community members feel their opinions are valued | Community participates in reciprocal dialogue at durbars | As noted above, *durbars* became venues for community members to provide feedback and to offer their perspectives on the efforts to promote maternal and child health. **Public interactions between community members and health workers also created rapport** between the two groups. Participants indicated that community members felt their opinions were valued because they were **allowed to express their views,** which encouraged **active participation and dialogue**. |
|  | *When health workers have existing relationships with community members (C) and they ask for their [community] feedback (R1), community members feel their opinions are valued because they have a relationship with the health worker (R2) and they engage in a reciprocal dialogue/open conversation with the health worker/programme representative.* | | | |
| **Relevant IPT(s)** | ***Notes**** | | | |
| IPT2/ IPT3 |  | | | |
| **CMOC5** | **Context** | **Mechanism** | **Outcome** | **Additional Information & Excerpts from Text** |
|  | Remote, difficult-to-reach communities | ***Resource:*** Interactive, reciprocal call-in component to radio show  ***Reasoning:*** Women feel they are able to ask questions that are more relevant and specific to their own concerns | Women/community learn about health services  🡪 Women seek care at hospital | Hospital-based QI teams (CS 10, 11, and 12) used radio to deliver health education to a large audience, especially in more **remote** communities that were difficult to reach by health workers. A CS11 biostatics technical officer explained that women who sought care at the hospital mentioned that these messages were influential in motivating women to avoid delaying care. Moreover, community members **could call in with questions during radio broadcasts**. Like the *durbars* that allowed for dialogue with communities, radio outreach also became an **interactive forum** that helped **address public concerns and questions about accessing health services, and enabled women to make informed decisions** about seeking care. |
|  | *In remote, difficult-to-reach communities (C), an interactive component to a radio/virtual messaging forum (R1) allows women feel they can ask questions that are more relevant and specific to their own concerns (R2) which improves their knowledge about health services (O1) which then increases their care-seeking behaviours (O2).* | | | |
| **Relevant IPT(s)** | ***Notes**** | | | |
| IPT2 | *Radio served as avenue to reach remote populations. Interactive broadcasts allowed for opportunities for reciprocal dialogue.* | | | |
| **CMOC6** | **Context** | **Mechanism** | **Outcome** | **Additional Information & Excerpts from Text** |
|  | Existing communications structures in the community (durbars, radio) | ***Resource:*** Messaging disseminated through existing communications structures in the community  ***Reasoning:*** Community members are familiar and accepting of programme messaging because it comes from a previously used source | Community uses and participates in the programme (attends meetings, calls into radio broadcasts). | Hospital-based QI teams (CS 10, 11, and 12) used radio to deliver health education to a large audience, especially in more **remote** communities that were difficult to reach by health workers. A CS11 biostatics technical officer explained that women who sought care at the hospital mentioned that these messages were influential in motivating women to avoid delaying care. Moreover, community members **could call in with questions during radio broadcasts**. Like the *durbars* that allowed for dialogue with communities, radio outreach also became an **interactive forum** that helped **address public concerns and questions about accessing health services, and enabled women to make informed decisions** about seeking care. |
|  | *In communities with existing communications structures (C), when that structure is used for dissemination (R1), the community is more likely to participate in that programme (O) because they are more familiar and accepting of the structure (R2).* | | | |
| **Relevant IPT(s)** | ***Notes**** | | | |
| IPT2 |  | | | |

**Paper #2**

| **Author** | **Year** | **Publication Type** | | **Aims/Objectives** | **Setting** |
| --- | --- | --- | --- | --- | --- |
| [Hounton et al.](https://www.ncbi.nlm.nih.gov/pmc/articles/PMC2779943/) | 2009 | Peer-Review Article | | To demonstrate the impact of a community mobilisation programme on maternal/perinatal mortality and describe implementation lessons for future scale up. | Burkina Faso |
| **Study Participants** | **Study Design** | **Findings** | | **Description of CE Communications Activities & Who they target** | **Models/Theoretical Frameworks** |
| Women experiencing pregnancy | Quasi-Experimental | Increase in institutional births and reduction in perinatal mortality in intervention districts | | Identify key influential leaders, engage stakeholders, implement BCC activities such as workshops, concerts, theatre, and monitor action plans and activities | Conceptual framework (Fig 1) of community mobilisation included four pillars: advocacy/awareness, social marketing, behavioural change communication (informed by health belief model), & capacity strengthening |
| **What is interesting about this paper?** | | **Richness Rating** | **Rigour Rating** | **Reference List/Snowballing** | |
| This paper includes specific detail around the activities/actions and the steps taken, as well as highlights the importance of context. | | High | Medium | Moran AC, Sangli G, Dineen R, Rawlins B, Yameogo M, Banza B. Birth preparedness for maternal health: findings from Koupela district, Burkina Faso. *J Health Popul Nutr.*2006;24:489–97.  Ouoba D, Congo Z, Diop NJ, Melching M, Banza B. Washington, DC: Population Council, Frontiers in Reproductive Health; 2004. Experience from a community based education program in Burkina Faso, the Tostan Program; p. 62. | |

| **CMOC7** | **Context** | **Mechanism** | **Outcome** | **Additional Information & Excerpts from Text** |
| --- | --- | --- | --- | --- |
|  | Community with low literacy levels and existing verbal communications structures | ***Resource***: Local leaders serve as messengers  ***Reasoning***: Community members respond to local leaders because of existing trust as a source of information | Increased knowledge amongst community members | In a context of low literacy, and where there is a value for community self-identification and where communities are responsive to recommendations of religious and traditional leaders, it is critical that any intervention in a community meets the needs of local stakeholders and wins their support. Communities are not ‘empty vessels’ and understanding and **building upon cultural beliefs of care and means of communication** is more likely to be effective. |
|  | *In communities with low literacy levels that rely on verbal communication structures (C), when local leaders serve as the messengers for the programme (R1), community members respond well because this is an existing and trusted source of information (R2) and their knowledge of the programme and its message priorities increases (O).* | | | |
| **Relevant IPT(s)** | ***Notes**** | | | |
| IPT2 | *Communicating in communities with low-literacy levels means emphasis/reliance on verbal forms of communication rather than written (pamphlets, etc). Verbal avenues such as spokespeople may be more typical modes of communication and so these existing/commonly-used sources should be built off of rather than introducing new avenues.* | | | |
| **CMOC8** | **Context** | **Mechanism** | **Outcome** | **Additional Information & Excerpts from Text** |
|  | Existing local leadership structures and gatekeepers who are influential and trusted in the community | ***Resource***: Local leaders are engaged and involved early in the programme  ***Reasoning***: Community members have existing trust and respect for local leaders | Community members accept the programme/messaging | In a context of low literacy, and where there is a value for community self-identification and where **communities are responsive to recommendations of religious and traditional leaders, it is critical that any intervention in a community meets the needs of local stakeholders and wins their support.** Communities are not ‘empty vessels’ and understanding and building upon cultural beliefs of care and means of communication is more likely to be effective. |
|  | *When local leaders are engaged early in the development of a programme and its messaging (R1) in a community that trusts these local leadership structures (C), community members accept the programme and its messaging (O) because they have an existing trust and respect for these local leaders (R2).* | | | |
| **Relevant IPT(s)** | ***Notes**** | | | |
| IPT1 | *Communities already trust and rely on local leaders and that respect and trust will translate to encourage them to accept programme messaging that comes from these same sources.* | | | |
| **CMOC9** | **Context** | **Mechanism** | **Outcome** | **Additional Information & Excerpts from Text** |
|  | Existing local leadership structures and gatekeepers who are influential and trusted in the community | ***Resource***: Local traditional leaders involved in the development and implementation of the community-based intervention process  ***Reasoning***: Community members feel a sense of social belonging when they obey traditional leaders | Increased demand for services as community members adhere to leaders’ recommendations | By obeying these traditional leaders people had **a sense of social belonging to the community**. Recognising the influence of these traditional leaders and their potential roles for engaging their communities in behavioural change, the SCI community mobilisation strategy selected the most influential ones and developed a **participatory approach with them**. . . structured meetings with prominent traditional leaders who, after an identification of main barriers to utilisation of maternal health services and possible solutions, invited their peers and other stakeholders (health professionals, religious leaders, administrative officials, associations) and engaged with them in the process ([Box 1](https://www.ncbi.nlm.nih.gov/pmc/articles/PMC2779943/#BX0001)). |
|  | *When local leaders are engaged early in the development of a programme and its messaging (R1) in a community that trusts these local leadership structures (C), community members adhere to the recommendations and increase their demand for health services (O) because they feel a sense of social belonging when they obey these traditional leaders (R2).* | | | |
| **Relevant IPT(s)** | ***Notes**** | | | |
| IPT1 | *Sense of social belonging is a motivating factor for community members to obey traditional leaders (mechanism explaining why?).* | | | |
| **CMOC10** | **Context** | **Mechanism** | **Outcome** | **Additional Information & Excerpts from Text** |
|  | Action-oriented community embracing change | ***Resource***: Closely working with/consulting communities in the design, planning, and implementation of programme  ***Reasoning***: Communities accept programme because they find it acceptable/relatable to (fits within) social norms and practices | Programme is sensitive/appropriate/ applicable to local contexts and structures 🡪 enabling continued participation and ownership | This investment in communities through an **understanding of their social structure** and health seeking behaviours, **through identification and partnership with credible community leaders**, and through identification **of culturally sensitive and locally acceptable approaches** to address transport and referrals was the single significant difference between the two districts. Although there is no magic bullet, nor a one-size-fits-all ideal community mobilisation approach, **closely working with communities by consulting them at all stages of design, planning and implementation of delivery care is critical** for achieving reduction in maternal and perinatal mortality. These demand-driven interventions require an **understanding the social structure of local contexts, the political and cultural logic of power, participation and ownership of the process** (hence communities not empty vessels) for a successful behavioural change strategy towards skilled delivery. |
|  | *In ‘active communities’ (C), consulting communities throughout all stages of the design and implementation of a programme (R1) will make the programme more acceptable and relatable to the community’s norms and practices (R2). This will make the programme appropriate and applicable to the community’s contexts and structures (O1) and encourage continued participation and ownership in the programme’s implementation (O2).* | | | |
| **Relevant IPT(s)** | ***Notes**** | | | |
| IPT3 | *Need better way to explain context 🡪 trying to describe community that is politically or social active and willing to incorporate changes and policies (as opposed to a purely hierarchical society where there is little impetus to incorporate changes). Trying to express that in communities that “care” about the challenge, involvement in all stages of development/implementation of programme will shape it in a way that is most appropriate (relevant & relatable) to them + encourage acceptance/buy-in because they understand it & it is compatible with norms/needs. Encourages continued participation and ownership which will create a continued positive cycle (sustainable).* | | | |
| **CMOC11** | **Context** | **Mechanism** | **Outcome** | **Additional Information & Excerpts from Text** |
|  | Existing communications structures in the community | ***Resource***: Programme works with community leaders to identify appropriate activities and communication avenues  ***Reasoning***: Modes of communication are familiar and accepted by the community | Increased knowledge 🡪 increased demand for services 🡪 increased service utilization | In partnership with community leaders, a number of **culturally acceptable activities were developed building on existing societal mechanisms**: community workshops, recruitment and posting of community relay agents in all villages of the intervention areas ([Box 2](https://www.ncbi.nlm.nih.gov/pmc/articles/PMC2779943/#BX0002)), public concerts with traditional singers, theatres and financial schemes to reduce out-of-pocket expenses during emergencies ([Box 3](https://www.ncbi.nlm.nih.gov/pmc/articles/PMC2779943/#BX0003)). |
|  | *In communities with existing communication structures (C), working with programme leaders to identify these communication avenues and activities (R1) will make messaging more accepted by the community because it is coming from familiar and previously used modes of communication (R2). By receiving these messages, community members’ knowledge will increase (O1) eventually leading to an increase in their demand and use of health services (O2).* | | | |
| **Relevant IPT(s)** | ***Notes**** | | | |
| IPT2 | *Similar to CMOC6 🡪 using familiar/existing structures means community will receive messages from typical source 🡪 likely to accept based on familiarity/trust of avenue* | | | |

**Paper #3**

| **Author** | **Year** | **Publication Type** | | **Aims/Objectives** | **Setting** |
| --- | --- | --- | --- | --- | --- |
| [Marcil et al.](https://www.ncbi.nlm.nih.gov/pmc/articles/PMC4794453/) | 2016 | Peer-Review Article | | To describe the community engagement, social mapping, and census taking of the BRAC Manoshi Project to strengthen health system in urban slums. | Bangladesh |
| **Study Participants** | **Study Design** | **Findings** | | **Description of CE Communications Activities & Who they target** | **Models/Theoretical Frameworks** |
| BRAC employees | Qualitative (Semi-Structured Interviews) | Using community engagement, social mapping, and census taking with strong ownership + sustainability were effective methods to improve community-based programming through better communication & knowledge of setting (logistics and information). Challenges come from transiency/migration | | Meeting with local leaders and representatives, cyclical feedback, mass marketing, social mapping and census taking | N/A |
| **What is interesting about this paper?** | | **Richness Rating** | **Rigour Rating** | **Reference List/Snowballing** | |
| This paper emphasizes trust as well as local ownership and knowledge of context/setting that largely inform even the functioning of a program and shows the value of CE by combining its impact for these seemingly different outcomes together. | | High | Low | Quayyum Z, Khan MN, Quayyum T, Nasreen HE, Chowdhury M, Ensor T. “Can community level interventions have an impact on equity and utilization of maternal health care”—evidence from rural Bangladesh. *Int J Equity Health.*2013;12:22.  Farmer PE, Nutt CT, Wagner CM, et al. Reduced premature mortality in Rwanda: lessons from success. *BMJ.*2013;346:f65.  Mugeni C, Levine AC, Munyaneza RM, et al. Nationwide implementation of integrated community case management of childhood illness in Rwanda. *Global Health, Sci Pract.*2014;2(3):328–41.  Bang AT, Bang RA. Background of the field trial of home-based neonatal care in Gadchiroli, India. *J Perinatol.*2005;25(Suppl 1):S3–10.  Aronson RE, Wallis AB, O’Campo PJ, Schafer P. Neighborhood mapping and evaluation: a methodology for participatory community health initiatives. *Matern Child Health J.*2007;11(4):373–83.  Roy T, Marcil L, Chowdhury R, Afsana K, Perry H. The BRAC Manoshi Approach to Initiating a Maternal, Neonatal and Child Health Project in Urban Slums with Social Mapping, Census Taking, and Community Engagement2014. Available at <http://www.brac.net/sites/default/files/portals/Manoshi-book-v3-1.pdf>. | |

| **CMOC12** | **Context** | **Mechanism** | **Outcome** | **Additional Information & Excerpts from Text** |
| --- | --- | --- | --- | --- |
|  | Suspicion/ mistrust of externally-implemented intervention programmes (because of previous experience) | ***Resource***: Programme roles out service provision early and quickly  ***Reasoning***: Community ‘trusts’ the programme because they have experienced benefits from its services | Community participates in/supports the programme | To build trust, BRAC initiated the service provision quickly, with the plan to adjust program focus later as needed. Local slum dwellers noted they had encountered numerous **pilot programs that suddenly stopped service provision**. This pattern had **created suspicion** among community members. As one BRAC staff member noted, *“They wanted to understand why we are here.* ***Everybody comes, talks, but does nothing.****”* Thus, to **gain trust**, BRAC found it important to first prove to the community that BRAC was serious about scaling their pilot program. Once the community members experienced benefits from the program, they became more willing to engage. |
|  | *In communities suspicious of externally-implemented interventions due to previous experience with such programmes (C), rolling out service provision early and quickly (R1) helps to build trust with the community by introducing them to the initial benefits from the programme (R2). As the community experiences these benefits and trusts the programme, they begin to participate and support it (O).* | | | |
| **Relevant IPT(s)** | ***Notes**** | | | |
| Not Applicable | *Previous programmes or interventions starting up and not being sustainable have disillusioned communities and caused them to be skeptical or mistrust similar programmes. To combat this, BRAC started rolling out services first so that communities would experience it and benefit from the programme. This early and initial provision of services was used to build trust and back up that the programme was sincere in its commitment. Once communities have already benefitted from the programme, they ‘believe’ in it when it comes up in the future.* | | | |
| **CMOC13** | **Context** | **Mechanism** | **Outcome** | **Additional Information & Excerpts from Text** |
|  | Existing local leadership structures and gatekeepers who are influential and trusted in the community | ***Resource:*** MNCH committees formed of local leaders from different sectors – local leaders involved in the programme  ***Reasoning:*** Committee’s knowledge provided guidance and legitimacy in the eyes of the community | Community members were willing to engage with the surveyors because they were individuals recommended by the local leaders | After service initiation, BRAC focused on creating Maternal, Neonatal, and Child Health (MNCH) Committees for defined geographic areas of approximately 10,000 people. Each committee consists of 9–11 local leaders from multiple sectors such as government, education, business, and religion. These committees provided indispensable **guidance and legitimacy** for the project from the perspective of the community. One BRAC staff member noted*, “A good quality MNCH committee [is essential] because this committee helped us select surveyors, some [of whom] actually became Shasta Kormis.”* These Committees **knew which individuals would be acceptable to the community** as surveyors for mapping and census taking. The Committees met bimonthly, with BRAC staff in attendance. During these meetings, BRAC updated the community about activities and progress, and the Committee gave feedback about problems. |
|  | *In communities with existing local leadership structures and trusted gatekeepers (C), involving local leaders in the programme’s oversight (R1) provided guidance and legitimacy in the eyes of the community (R2), influencing the community’s willingness to engage with the programme (O) because of this perceived legitimacy.* | | | |
| **Relevant IPT(s)** | ***Notes**** | | | |
| IPT1 | *This was also a bit of a tricky one…the MNCH committees involve local leaders from multiple different sectors which provides important contextual information, guidance, legitimacy, and demonstrates their ‘buy-in’ to the rest of the community. They then identify potential ‘surveyors’ who will be accepted by the community (based on their knowledge as well as their endorsement) and the community members are willing to engage with these surveyors because of their leaders’ endorsements.* | | | |
| **CMOC14** | **Context** | **Mechanism** | **Outcome** | **Additional Information & Excerpts from Text** |
|  | Existing communications structures in the community | ***Resource***: Playing advertisements with the BRAC logo on mass media channels (local popular method was to watch shopfront’s TVs)  ***Reasoning***: Community trusts regularly watched screens that they are familiar with as source of information | Community members were receptive to the programme early on | Prior to project implementation, BRAC provided information about the project through **mass media channels accessible** to slum dwellers. In Bangladesh, **most market shops have small TVs, which are popular to watch**. BRAC ran paid project advertisements, which **familiarized slum dwellers with both the BRAC logo and message.** Consequently, people were more receptive to the program at its inception. |
|  | *In communities with existing communications structures and forums (C), using these forums to share recognizable information about the programme such as the logo (R1) will expose community members to it in their customary practices that they are already familiar with (R2). This recognition will make community members more receptive to the programme (O).* | | | |
| **Relevant IPT(s)** | ***Notes**** | | | |
| IPT2/IPT3 | *Similar to CMOC6. There are two key components of this CMOC and maybe that means it should be broken into two or there are two mechanisms? One is using the standard communication channel that is commonly used by the community – in this case the TVs in the storefronts – this is their standard communication method and so it is well-known, often used, trusted, etc. The other component is the repeated exposure to a symbol eventual recognition. I’ve put this in one CMOC because I think it is these two components combined that are important 1) it’s a regularly used/trusted source and 2) there’s the repeat exposure 🡪 these together create a sort of trusted recognition. Am not sure if this comes out as explicitly in this draft of the CMOC.* | | | |
| **CMOC15** | **Context** | **Mechanism** | **Outcome** | **Additional Information & Excerpts from Text** |
|  | Existing communications structures in the community | ***Resource:*** Repeated exposure to the BRAC logo on usual mass media channels  ***Reasoning:*** Community becomes familiar and recognizes BRAC logo | Community members were receptive to the programme early on | Prior to project implementation, BRAC provided information about the project through **mass media channels accessible** to slum dwellers. In Bangladesh, **most market shops have small TVs, which are popular to watch**. BRAC ran paid project advertisements, which **familiarized slum dwellers with both the BRAC logo and message.** Consequently, people were more receptive to the program at its inception. |
|  | *In communities with existing communications structures and forums (C), repeatedly exposing community members to the programme and its logo/symbolism on these channels (R1) will make them familiar with the programme (R2). This recognition will make community members more receptive to the programme (O).* | | | |
| **Relevant IPT(s)** | ***Notes**** | | | |
| IPT2+ | *Not similar to CMOC6 and now potentially more distinct from CMOC14. Broke down what was identified as the two potential mechanisms in CMOC14 🡪 now the Reasoning in CMOC14 is specifically about the source and the fact that they trust it. CMOC15 now highlights repeated exposure as the resource and so the reasoning is a familiarity with and recognition of this logo/the programme.* | | | |
| **CMOC16** | **Context** | **Mechanism** | **Outcome** | **Additional Information & Excerpts from Text** |
|  | Community observes informal/ traditional care practices during pregnancy | ***Resource***: Incorporating traditional practices by retraining existing local practitioners/providers  ***Reasoning***: Women are comfortable with existing care providers (such as TBAs) | Women utilize the health services | BRAC found that **incorporating traditional practices** into project services further increased community engagement. For instance, TBAs are women lacking formal training but with much experience attending deliveries. They are an integral part of birthing in Bangladesh. BRAC decided to incorporate them into the project as trained UBAs instead of trying to remove them, which might have caused community resistance. BRAC has found that **women are more comfortable utilizing its services when UBAs with whom they are already familiar** provide care. |
|  | *In communities with informal/traditional care practices (C), incorporating these practices by retraining the local providers (R1) will encourage women to utilize the health services (O) because they are comfortable with their usual/existing care providers, such as TBAs (R2).* | | | |
| **Relevant IPT(s)** | ***Notes**** | | | |
| IPT3+ | *Women already trust or have relationships with existing providers, + maybe cultural sensitivity around program by incorporating them rather than working around them?* | | | |
| **CMOC17** | **Context** | **Mechanism** | **Outcome** | **Additional Information & Excerpts from Text** |
|  | Transient communities | ***Resource***: Social mapping and census conducted to elicit relevant/contextual information about community, with leaders present  ***Reasoning***: People cooperate because they trust the community leader | People provide relevant and honest information 🡪 Project is relevant and appropriate to the community | Both social mapping and census taking are a means of community engagement, valuable in its own right but also a means to obtain important programming information. Through these processes, the communities **became familiar with the project and experienced BRAC’s commitment** **to understanding their needs**. BRAC found that having **key community leaders intermittently present alongside** the surveyors further increased acceptance of the social mapping and census-taking activities. |
|  | *In transient communities where there is limited information on the structure and available resources (C), conducting censuses and social mapping can help elicit relevant, contextual information about the community (activity). If a community leader is present when these activities are carried out (R1), then people are more likely to cooperate because they know and trust the community leader (R2) and provide relevant and honest information (O1) that can inform the project to be most relevant and appropriate to the community (O2).* | | | |
| **Relevant IPT(s)** | ***Notes**** | | | |
| IPT1/IPT3 | *This CMOC may be too specific in terms of the Mechanisms?*  *But also somewhat similar to CMOC13 in terms of the legitimacy/trust that the local leaders provide (so same reasoning but with different resources?)*  *Struggling with this one…in communities that are transient, there may not be as much knowledge/awareness of who lives where and what resources are available. Social mapping and census taking helps to address these challenges. By then being able to use this information, the project can adapt and incorporate components that make it most relevant and appropriate to the community as well as logistically possible (also for example resource mobilisation). For this social mapping and census taking to work, the people they speak with must be honest and willing to contribute. By involving community leaders at various times in the process, people felt they could trust and work with the surveyors. So then is the outcome the people’s cooperation because (reasoning) they trusted/accepted the surveyors? Or is their cooperation because of trust the reasoning?* | | | |
| **CMOC18** | **Context** | **Mechanism** | **Outcome** | **Additional Information & Excerpts from Text** |
|  | Low uptake of NGO-implemented intervention | ***Resource***: Conversations/ dialogue helped establish relationships between families and programme (NGO)  ***Reasoning***: Community members feel their feedback is being heard and responded to | Increased service uptake | Despite all these efforts, BRAC found that **uptake of services was low at the outset**. Instead of being discouraged, BRAC used a **cyclical process of feedback** to encourage community engagement. Through **continued conversations** with the community and **establishing relationships** with the families, slum dwellers observed that the program was **committed to responding to their feedback** and to providing quality services. Ultimately, **service uptake increased substantially over time**. |
|  | *When there is low uptake in the community of an NGO-implemented intervention (C), conversations and open dialogue helped to establish relationships between the families and the programme (R1) which allowed the community members to feel like their feedback was being heard and incorporated (R2) and increase their service uptake over time (O).* | | | |
| **Relevant IPT(s)** | ***Notes**** | | | |
| IPT2/IPT3 | *Some similarities to CMOC4 in terms of open/reciprocal dialogues.*  *In this CMOC, it was useful to point out and specify that part of the context of this programme is that it is externally-implemented or led by an NGO. There is limitted uptake at the beginning of the intervention’s implementation (this could be for a number of reasons that aren’t specified, including other contextual reasons?)* | | | |
| **CMOC19** | **Context** | **Mechanism** | **Outcome** | **Additional Information & Excerpts from Text** |
|  | Women participate in the NGO-implemented programme | ***Resource***: Women endorse the programme and become more active in the programming itself  ***Reasoning***: Other women/community members listen to the advice of their peers because they know and trust them | Sustainable programming | Although the effort was led by BRAC, over time, **more initiative has come from the community.** For example, women actively referred other women to use BRAC/Manoshi services, members of the MNCH Committees became more active (including monitoring of program activities and carrying out social autopsies on all maternal deaths and selected neonatal deaths), and as women’s groups developed their own funds to help pay for the expenses of women who required hospitalization for delivery complications. |
|  | *When local women and community members participate in the NGO programme (C) and endorse/become more active in the programme or champion the programme (R1), the programme and improvements it targets become more sustainable (O). This is because other women/community members listen to the advice of their peers because they know and trust them (R2).* | | | |
| **Relevant IPT(s)** | ***Notes**** | | | |
| Not Applicable | *Not sure if this is a reasonable context but it could be the type of situation where the outcome of some of the previous CMOCs (aka involvement/participation in the programme) has become the context for this CMOC – the fact that the programme is functioning and people are involved.* | | | |

**Paper #4**

| **Author** | **Year** | **Publication Type** | | **Aims/Objectives** | **Setting** |
| --- | --- | --- | --- | --- | --- |
| [Ntoimo et al.](https://www.ajol.info/index.php/ajrh/article/view/216027) | 2021 | Peer-Review Article | | To describe how a community-based participatory approach was used to involve community leadership in the design and implementation of a project to increase access to maternal and child health and the implications/lessons for future settings | Nigeria |
| **Study Participants** | **Study Design** | **Findings** | | **Description of CE Communications Activities & Who they target** | **Models/Theoretical Frameworks** |
| Wards (unclear) + key informants from the community | Qualitative (key informant interviews)  [overall project was quasi-experimental (pretest-posttest)] | Community/women reported positive perceptions of the community engagement approach. Interventions (designed based on formative stage of research) were effective in increasing ANC, delivery, PNC by four-times and childhood vaccinations by three-times. | | Advocacy activities and engagement with key stakeholders, community conversations, ward development committees, community sensitization workshops | N/A |
| **What is interesting about this paper?** | | **Richness Rating** | **Rigour Rating** | **Reference List/Snowballing** | |
| The paper describes how the community was engaged throughout the intervention. Most importantly, it emphasizes involving the community from the beginning ***throughout*** the project life cycle and bottom-up decision-making process that is “informed by knowledge and cultural preference of the local communities” as well as transparency + accountability. | | High | Medium | Okonofua FE, Ntoimo LFC, Ogungbangbe J, Anjorin S, Imongan W and Yaya S. *Predictors of Women’s Utilization of Primary Health Care for Skilled Pregnancy Care in Rural Nigeria*. BMC Pregnancy and Childbirth. 2018;18:106.  Fantaye AW, Okonofua F, Ntoimo L and Yaya S. *A qualitative study of community elders’ perceptions about the underutilization of formal maternal care and maternal death in rural Nigeria.* Reproductive health. 2019;16(1):1-17.  Ntoimo LFC, Okonofua FE, Igboin B, Ekwo C, Imongan W and Yaya S. *Why rural women do not use primary health centres for pregnancy care: evidence from a qualitative study in Nigeria.* BMC Pregnancy and Childbirth. 2019;19(1):1-13.  Yaya S, Okonofua F, Ntoimo L, Udenigwe O and Bishwajit G*. Men’s perception of barriers to women’s use and access of skilled pregnancy care in rural Nigeria: a qualitative study.* Reproductive health. 2019;16(1):86.  Okonofua FE, Ntoimo LF, Yaya S,Igboin B, Solanke O, Ekwo C, Johnson E, Sombie I and Imongan W.. *Effect of a Multifaceted Intervention on Utilization of Primary Health Care for Maternal and Child Health Care in Rural Nigeria: A quasi-experimental study.* BMJ Open (in review).  Yaya S, Okonofua F, Ntoimo L, Udenige O and Bishwajit G. *Gender inequity as a barrier to women’s access to skilled pregnancy care in rural Nigeria: a qualitative study.* International Health. 2019;11(6):551-560. | |

| **CMOC20** | **Context** | **Mechanism** | **Outcome** | **Additional Information & Excerpts from Text** |
| --- | --- | --- | --- | --- |
|  | Existing local leadership structures and gatekeepers who are influential and trusted in the community | ***Resource***: Involving community leaders early  ***Reasoning***: Community respects leaders and their buy-in/endorsement of a programme | Programme is accepted by the community | Pg 46:  **Traditional rulers, their council of chiefs, and elders command high respect** and influence in the project communities. We leveraged this to achieve community buy-in, support, and involvement. The advocacy team identified the influential traditional rulers and through the gatekeepers, **meetings** were scheduled with them.  Noteworthy is that the traditional rulers were **closely followed up on their promises to ensure they did not renege on their support.** This was achieved by giving them a **continuous sense of belonging and ownership by asking for their input, advice, and action at the different stages** of the project.  Pg 50:  *Our community leader His Royal Highness and Chiefs were also key players in the program.* |
|  | *In communities with existing local leadership structures (C), involving these community leaders early (R1) will improve the community’s acceptance of the programme (O) because they respect their local leaders and their endorsements (R2).* | | | |
| **Relevant IPT(s)** | ***Notes**** | | | |
| IPT1 | *Very similar to CMOC8 and maybe CMOC13 a little bit?* | | | |
| **CMOC21** | **Context** | **Mechanism** | **Outcome** | **Additional Information & Excerpts from Text** |
|  | Existing local leadership structures and gatekeepers who are influential and trusted in the community | ***Resource***: Continuous and follow-up engagement with community leaders  ***Reasoning***: Community leaders felt a sense of belonging and ownership over the programme | Community leaders stayed involved throughout the stages of the programme | Pg 46:  **Traditional rulers, their council of chiefs, and elders command high respect** and influence in the project communities. We leveraged this to achieve community buy-in, support, and involvement. The advocacy team identified the influential traditional rulers and through the gatekeepers, **meetings** were scheduled with them.  Noteworthy is that the traditional rulers were **closely followed up on their promises to ensure they did not renege on their support.** This was achieved by giving them a **continuous sense of belonging and ownership by asking for their input, advice, and action at the different stages** of the project.  Pg 50:  *Our community leader His Royal Highness and Chiefs were also key players in the program*. |
|  | *In communities with existing local leadership structures (C), continuously following up with community leaders (R1) will keep the community leaders involved throughout all stages of the programme (O) because they feel a sense of belonging and ownership over the programme (R2).* | | | |
| **Relevant IPT(s)** | ***Notes**** | | | |
| IPT1/IPT3 | *Important part of this CMOC is probably the follow-up component. Additionally, the belonging and ownership are reasonings/mechanisms that have come up in other CMOCs but not in this way. Here, the local leaders are the ones who have a sense of belonging and ownership and perhaps this influences their likelihood to endorse the programme because they feel this connection/ownership of it?* | | | |
| **CMOC22** | **Context** | **Mechanism** | **Outcome** | **Additional Information & Excerpts from Text** |
|  | Existing local leadership structures and gatekeepers who are influential and trusted in the community | ***Resource:*** Programme observes traditional practices in reaching out to community leaders  ***Reasoning:*** Community leaders feel respected and appropriately addressed | Community leaders are open to listening to the programme objectives | Pg 46:  **Observing the traditional rites as regards** gifts of wine, kola nut, etc., the research team introduced the project by presenting the challenge of maternal and child health to the traditional rulers, and then the project goals. |
|  | *In communities with existing local leadership structures (C), observing local traditional practices while reaching out to community leaders (R1) will demonstrate respect to the community leaders (R2) and influence them to be open to the meeting and listening to the programme objectives (O).* | | | |
| **Relevant IPT(s)** | ***Notes**** | | | |
| IPT1/IPT3 | *Following traditional rites/practices to demonstrate respect. This is an important enabling factor in the development of relationship between leader and programme.* | | | |
| **CMOC23** | **Context** | **Mechanism** | **Outcome** | **Additional Information & Excerpts from Text** |
|  | Externally-implemented intervention  +  Community lacks health services/access (perceived need) | ***Resource***: Programme provides needed services to the community  ***Reasoning***: Community members believe in the programme/intervention | Community members are happy with the benefits from the programme and accept it | Pg 48:  *So, when I look at it they came with some Professors and Doctors that explained it better. So now I know* ***that they came here for a serious thing and they want to take care of my community****. What they did is* ***that all that we complained about they took care of them.*** *So, after a few days, they ask me to provide the Chairman [WDC] and I did. They came back and told me they went to the clinic and saw the nasty bed they were using. They went back home and provided all the drugs and beds for the maternity, then* ***I now discovered that they were not playing that they are very serious****, … You know when you come to the rural area and talk about money, the people will shift back but* ***when they saw the good things coming from it they will all accept it. So far so good I am happy*** *with the project that WHARC has done for the community (Traditional Ruler 1).*  *Well, in our community here we are* ***very far from the hospital*** *[secondary hospital], the nearest hospital to this place is about 40km if not more than so we have suffered lack of proper medical service for a long time. It happened that at the time WHARC came to us a woman had a quadruplet birth in our community, the woman almost lost her life and none of the children survived. So,* ***at the time WHARC program came, it was just at the right time*** *and* ***when His Royal Highness [Traditional***  ***Ruler] heard of the program, he supported it.*** *So when they came they used a lot of money in revamping the health facility, providing essential drugs for pregnant women, and even created a health insurance scheme where women contribute very little but enjoy the benefits of all service before and after they deliver.* ***Since WHARC came, we have noticed a great change in women's attitude in attending the health centre*** *(WDC Chairman 2).* |
|  | *When an intervention is implemented by an external group (e.g. NGO) in a community that lacks health services/access (C), providing needed services that benefit the community (R1) will establish the community’s belief/trust in the programme (R2) and the community will therefore accept the programme because they have received its benefits (O).* | | | |
| **Relevant IPT(s)** | ***Notes**** | | | |
| Not Applicable | *This informing passage/excerpt is basically about the programme ‘putting their money where their mouth is’ and demonstrating that they are serious and will do the work they commit to. Similar to CMOC12 they are providing some service/doing some work that the community or individuals then perceive a benefit from which makes them realize they are committed (a trust/belief in the programme?)*  *Another useful component of this one could be the fact that they were specifically acting on feedback?* | | | |
| **CMOC24** | **Context** | **Mechanism** | **Outcome** | **Additional Information & Excerpts from Text** |
|  | Externally-implemented intervention | ***Resource***: Programme responds to complaints/feedback from community  ***Reasoning***: Community members feel their complaints are heard by the programme | Community members are happy with the benefits from the programme and accept it | Pg 48:  *So, when I look at it they came with some Professors and Doctors that explained it better. So now I know* ***that they came here for a serious thing and they want to take care of my community****. What they did is* ***that all that we complained about they took care of them.*** *So, after a few days, they ask me to provide the Chairman [WDC] and I did. They came back and told me they went to the clinic and saw the nasty bed they were using. They went back home and provided all the drugs and beds for the maternity, then* ***I now discovered that they were not playing that they are very serious****, … You know when you come to the rural area and talk about money, the people will shift back but* ***when they saw the good things coming from it they will all accept it. So far so good I am happy*** *with the project that WHARC has done for the community (Traditional Ruler 1)* |
|  | *When an intervention is implemented by an external group (e.g. NGO) (C), responding to the community’s complaints and feedback directly (R1) will make community members feel heard by the programme (R2) and the community will therefore accept the programme because they have received its benefits (O).* | | | |
| **Relevant IPT(s)** | ***Notes**** | | | |
| IPT2 | *Is this too similar to CMOC23?* | | | |
| **CMOC25** | **Context** | **Mechanism** | **Outcome** | **Additional Information & Excerpts from Text** |
|  | Underutilized health services | ***Resource***: Local women serve as champions and describe the benefits/successes of the intervention  ***Reasoning***: Because they have experienced benefits from the intervention and their peers listen to them | Sustainable increase in utilization beyond the project | Pg 49:  *The rate of use has increased. Initially, when I came into this place, pregnant women were not coming to the clinic, until WHARC came,* ***everybody was like free this, free discharge so it really helped us****. All the women in this place used to go to nearby village birth attendants. With the help of WHARC* ***women have been patronizing the health center very well*** *(PHC Nurse 1)*  Pg 50:  *The PHCs in the project communities were highly underutilized before the IMCHA project. According to the participants,* ***the intervention increased utilization because women who benefited from the intervention became campaign agents for the project in their communities.*** *Then the women were now campaigning in each of the villages because we have about 20 villages. (Traditional Ruler 1).* |
|  | *In communities where health services are underutilized (C), having local women serve as champions by describing the benefits of the intervention (R1) will encourage their peers/neighbors to participate because they listen to/trust the women’s firsthand experience (R2). This will enable sustainable increases in utilization beyond the project itself (O).* | | | |
| **Relevant IPT(s)** | ***Notes**** | | | |
| Not Applicable | *Reasoning is not very obvious for this one – local women serve as champions to villages because they have benefitted from the programme. This means that they are then serving as champions beyond the project which enables a continued increase in service utilization.*  *Makes a little more sense now! Raving about experiences at the health center will encourage their peers to attend because they believe/trust their experiences. This can affect utilization beyond the study/programme period because women will continue to share their experiences, with or without the external intervention* | | | |
| **CMOC26** | **Context** | **Mechanism** | **Outcome** | **Additional Information & Excerpts from Text** |
|  | Existing local leadership structures and gatekeepers who are influential and trusted in the community | ***Resource***: Community leaders identify individuals to conduct/lead work  ***Reasoning***: Community members trust these individuals because of the community leaders’ endorsement | Community members and stakeholders actively participate in the programme | Pg 52:  The **identification of the WDCs by community leaders** enabled the **selection of the most trusted and reliable persons** in the community to guide the process. **Trust, integrity, and accountability** then became the most important elements that underpinned the project delivery and that ensured the active participation of all stakeholders in the community. Even after the study ended, these **elements of the project ensured the continuation and sustainable delivery** of all activities related to the project. |
|  | *In communities with existing local leadership structures and trusted gatekeepers (C), involving local leaders in the identification of staff/workers (R1) meant the community members trusted these individuals because of their leaders’ endorsement (R2), encouraging their active participation in the programme (O).* | | | |
| **Relevant IPT(s)** | ***Notes**** | | | |
| IPT1/IPT3 | *Similar to CMOC13.* | | | |

**Paper #5**

| **Author** | **Year** | **Publication Type** | | **Aims/Objectives** | **Setting** |
| --- | --- | --- | --- | --- | --- |
| [Rath et. al](https://bmcinthealthhumrights.biomedcentral.com/track/pdf/10.1186/1472-698X-10-25.pdf) | 2010 | Peer-Reviewed Article | | Describe the intervention in theory and practice, describe social context in which the intervention was delivered, describe the impact of intervention in group and non-group members | India |
| **Study Participants** | **Study Design** | **Findings** | | **Description of CE Communications Activities & Who they target** | **Models/Theoretical Frameworks** |
| Group facilitators, women’s group members, community members, stakeholders | Qualitative (discussions, meeting observations, review of documents) | Factors influencing the impact of the intervention included: acceptability, participatory approach, community involvement, focusing on marginalized communities, active recruitment of pregnant women, high population coverage. Resulted in increase in safe delivery and care practices and decreased neonatal mortality. | | Women’s group (PLA cycle) with pregnant/recently pregnant women | Warmi Project & Makwanpur Women’s Group cycles  Participatory Learning Action Cycle |
| **What is interesting about this paper?** | | **Richness Rating** | **Rigour Rating** | **Reference List/Snowballing** | |
| This paper goes into a lot of detail on the *what* was done in terms of the processes and implementation of the women’s groups and what the PLA cycle looked like as well as how meetings were scheduled, facilitators recruited, etc. Interesting that they conceptualize the word ‘mechanisms’ more as project components/*maybe* resources. However, the majority of these ‘mechanisms’ are trying to explain changes in mortality outcomes and so not always relevant (for example, the one that doesn’t come through in this form is related to how they targeted vulnerable, poor, least healthy population; this is not then connected to communications or the community mobilisation itself). Would have liked to see a more in terms of explanatory power behind the ‘mechanisms’ they draw out and what it is about these things that they hypothesize influences their outcomes. | | High | Medium | Howard Grabman L: Planning together: developing community plans to address priority maternal and neonatal health problems in rural Bolivia. In Participatory research in health issues and experiences. Edited by: De Koning K, Martin M. London, Zed Books; 1996:153-163 | |

| **CMOC27** | **Context** | **Mechanism** | **Outcome** | **Additional Information & Excerpts from Text** |
| --- | --- | --- | --- | --- |
|  | Rural, tribal communities with distinct identities | ***Resource***: Facilitators were recruited locally  ***Reasoning***: Community trusts facilitators | Positive interaction or relationship between the facilitator and the community | Pg 4:  The intervention areas were rural, largely tribal, and covered a population of 114 141, including 193 villages and 254 hamlets. Several tribal or adivasi (indigenous) groups inhabit these areas, including Ho, Santhal, Juang, Bhuiyan, Oraon and Munda communities. In both Jharkhand and Orissa, adivasi groups have distinct identities and strive to safeguard their social institutions and ancestral territories.  Fig 4:  *Implementation Methods:* Local facilitators recruited with help from the community  Pg 6:  Three main factors enhanced the intervention’s acceptability**: the recruitment and training of local facilitators,** the use of locally appropriate discussion materials in meetings, and flexibility in the timing and content of meetings.  Pg 7:  Facilitators earned the **community’s trust by being from the study area**, **respecting local practices**, and **knowing local languages**.  *As I am from the same community it is easier for me to interact with the group and understand their health situation. Knowing the local language makes communication easier. (Facilitator, Keonjhar, Phase 3 FGD)*  *She is from our community, she is a friend, she helps us in solving our problems and makes us aware of the problems we suffer from by using picture cards and games, we* ***consider her as a part of us and trust her****. (Group member, West Singhbhum, Phase 3 FGD).* |
|  | *In rural, tribal communities that protect their distinct identities (C), recruiting facilitators locally (R1) will foster trust between the community and the facilitators (R2) and enable a positive relationship between the facilitator and the community (O).* | | | |
| **Relevant IPT(s)** | ***Notes**** | | | |
| IPT1 | *Locally recruited staff is known, trusted, speaks same language 🡪 enables positive relationship (maybe existing relationships??) with participants/community members* | | | |
| **CMOC28** | **Context** | **Mechanism** | **Outcome** | **Additional Information & Excerpts from Text** |
|  | Rural, tribal communities with distinct identities | ***Resource***: Facilitators speak the local languages  ***Reasoning***: Community trusts facilitators | Open/clear communication between the facilitator and the group | Fig 4:  *Implementation Methods:* Local facilitators recruited with help from the community  Pg 7:  Facilitators earned the **community’s trust by being from the study area**, **respecting local practices**, and **knowing local languages**.  *As I am from the same community it is* ***easier for me to interact with the group and understand their health situation. Knowing the local language makes communication easier.*** *(Facilitator, Keonjhar, Phase 3 FGD)*  *She is from our community, she is a friend, she helps us in solving our problems and makes us aware of the problems we suffer from by using picture cards and games, we consider her as a part of us and trust her. (Group member, West Singhbhum, Phase 3 FGD).* |
|  | *In rural, tribal communities that protect their distinct identities (C), recruiting facilitators who speak the local languages (R1) will foster trust between the community and the facilitators (R2) and enable clear and open communication between the facilitator and the community (O).* | | | |
| **Relevant IPT(s)** | ***Notes**** | | | |
| IPT1 | *Speaking same language means they can communicate appropriately and normally in a way that community can understand* | | | |
| **CMOC29** | **Context** | **Mechanism** | **Outcome** | **Additional Information & Excerpts from Text** |
|  | Rural, tribal communities with distinct identities | ***Resource***: Facilitators respect local practices  ***Reasoning***: Community trusts facilitators | Positive interaction or relationship between the facilitator and the community | Fig 4:  *Implementation Methods:* Local facilitators recruited with help from the community  Pg 7:  Facilitators earned the **community’s trust by being from the study area**, **respecting local practices**, and **knowing local languages**.  *As I am from the same community it is* ***easier for me to interact with the group and understand their health situation. Knowing the local language makes communication easier.*** *(Facilitator, Keonjhar, Phase 3 FGD)* |
|  | *In rural, tribal communities that protect their distinct identities (C), facilitators who respect local practices (R1) will foster trust between the community and the facilitators (R2) and enable a positive relationship between the facilitator and the community (O).* | | | |
| **Relevant IPT(s)** | ***Notes**** | | | |
| IPT3 | *This CMOC may not be as necessary? Not much detail. Instead this data may be able to support another CMOC from a different paper* | | | |
| **CMOC30** | **Context** | **Mechanism** | **Outcome** | **Additional Information & Excerpts from Text** |
|  | Existing local leadership structures and gatekeepers who are influential and trusted in the community | ***Resource:*** Local leaders and community are involved in the selection of facilitators  ***Reasoning:*** Women’s group participants trust the facilitators because of endorsement ***OR*** because they feel sense of connection/similarity/ belonging coming from the same community? | Community members are accepting of the facilitators | Fig 4:  *Implementation Methods:* Local facilitators recruited with help from the community  Pg 7:  In order to select facilitators, **focus group discussions were held with elders, opinion leaders, headmen and women** in three randomly chosen intervention clusters to identify selection criteria. Preference was given to local, literate married women, preferably daughters-inlaw from the selected villages who had supportive families and could travel independently to conduct meetings.  Facilitators earned the community’s trust by being from the study area, respecting local practices, and knowing local languages. |
|  | *In communities with existing local leadership structures and trusted gatekeepers (C), involving local leaders in the selection of facilitators (R1) encourages trust between the women’s group participants and the facilitators (R2), influencing the participants willingness to accept and engage with the facilitators (O) because of this endorsement from the local leaders.* | | | |
| **Relevant IPT(s)** | ***Notes**** | | | |
| IPT1 | *Similar to the majority of the other leadership-recruitment related CMOCs, however this one involves general community members in recruitment as well so not only is there this transitive property of trust but they also trust their OWN judgment and feel the facilitator is ‘one of them’ because they are coming from the same place. Potentially revisit this CMO to make sure it is clear.* | | | |
| **CMOC31** | **Context** | **Mechanism** | **Outcome** | **Additional Information & Excerpts from Text** |
|  | Tribal communities with distinct identity and traditions, including nature worship and belief in supernatural causes of health problems | ***Resource:*** Appropriate and relevant content discussed at meeting is put into practice  ***Reasoning:*** Community members are open to the messaging and experience benefits from the implemented solutions which validates meetings’ value | Increased/improved trust | Pg 4:  Most adivasi communities in the study area were nature worshippers and interacted ritually with supernatural beings believed to reside in the home and natural environment. Health problems and illnesses were thus often attributed to supernatural causes and local diviners or private providers were commonly used to deal with problems in pregnancy and newborn illnesses.  Fig 4:  *Implementation Methods:* Appealing and flexible meeting content  Pg 7:  *Group members believe our words and the contents discussed during the meetings. They implement them and* ***when they get the benefits their trust strengthens.*** *(Facilitator, Saraikela Kharsawan, Phase 4 FGD)*  *Through story telling we could know some harmful practices and realised that because of some of the ageold practices many mothers and newborns might have lost their lives. (West Singhbhum, meeting 6)* |
|  | *In rural, tribal communities with distinct identities, traditions, and beliefs (C), when appropriate and relevant content from meetings is put into practice (R1), benefitting from this advice will validate the meetings’ value in the eyes of the community members (R2) and increase their trust of the program (O) because they have benefitted from it.* | | | |
| **Relevant IPT(s)** | ***Notes**** | | | |
| IPT2 | *Fairly straightforward – in the community of interest, their identify is important and beliefs are intertwined with experiences of health problems. By incorporating this into the content that the women’s group participants develop themselves, the community members are open to the messaging and may implement it – the benefits they then receive would validate the messaging. This first part about openness/acceptance of messaging may not currently come through as clearly in CMOC?* | | | |
| **CMOC32** | **Context** | **Mechanism** | **Outcome** | **Additional Information & Excerpts from Text** |
|  | Tribal communities with distinct identity and traditions, including nature worship and belief in supernatural causes of health problems | ***Resource***: Locally-adapted and developed activities (picture cards, stories, games)  ***Reasoning***: Messaging and content are culturally appropriate which makes them acceptable and relevant | Increased understanding and knowledge | Fig 4:  *Implementation Methods:* Discussion of local practices related to pregnancy and childbirth  Pg 7:  Facilitators felt that the **production and iterative adaptation of locally appropriate** picture cards, stories, and participatory games increased **acceptability** and catalysed learning and planning within the groups. During the pilot phase, innovative facilitation methods were tried out and suitable techniques selected so that each women’s group meeting had new activities, was participatory, and took less than two hours.  *Through story telling we could know some harmful practices and realised that because of some of the ageold practices many mothers and newborns might have lost their lives. (West Singhbhum, meeting 6)* |
|  | *In rural, tribal communities with distinct identities, traditions, and beliefs (C), developing locally-adapted and participatory activities in the group (R1) will make the messaging and content culturally appropriate and therefore more acceptable and relevant to the group’s needs (R2). This will then increase understanding and knowledge (O).* | | | |
| **Relevant IPT(s)** | ***Notes**** | | | |
| IPT2 | *A bit similar to CMOC31, but with more of the focus on what makes them willing to implement (and then benefit from) the messaging is the fact that it is culturally appropriate, acceptable, and relevant* | | | |
| **CMOC33** | **Context** | **Mechanism** | **Outcome** | **Additional Information & Excerpts from Text** |
|  | Rural, tribal communities with poor health care delivery | ***Resource***: Participatory approach to meetings  ***Reasoning***: Sharing experiences/problems in the group helped participants problem-solve and build confidence | Increased understanding and knowledge | Fig 4:  *Implementation Methods:* Participatory approach to learning and problem solving  Pg 7:  The implementation team and group members suggested that the structured, phase-wise content of the meeting cycle and its emphasis on **collective problem solving contributed to learning and confidence building**. This appears to have been a key determinant of the intervention’s efficacy and acceptability. The following quotes from group members illustrate this:  *We* ***could not do much as individuals but as a group we could find a way to solve each other’s problems.*** *(Keonjhar, meeting 3)*  *Through story telling* ***we could know some harmful practices and realised that because of some of the ageold practices many mothers and newborns might have lost their lives****. (West Singhbhum, meeting 6)*  *It was easy to understand the causes and effects of maternal and newborn problems* ***through picture card stories****. (Saraikela Kharsawan, meeting 6)*  *By* ***sharing experiences with members of other groups we can learn from each other*** *about the strategies that have benefited them. (Phase 4 FGD)*  *Review of the implemented strategies in each meeting helped us in performing our responsibilities properly. (Phase 4 FGD)* |
|  | *In rural, tribal communities with poor health care delivery (C), participatory approach to women’s group meetings (R1) allows participants to share their experiences in the group and build problem-solving skills and confidence (R2). This increased confidence and shared-learning with improve understanding and knowledge (O).* | | | |
| **Relevant IPT(s)** | ***Notes**** | | | |
| IPT2 | *Not totally sure about the reasoning here. Important components are that the community context may be that they are rural/spread out and don’t always have access to the service/health delivery. By being participatory in nature, participants share their experiences that they may not have realized others have experience with and this brings them together as a collective. They improve their problem-solving abilities by learning from each other and also increase their confidence by being able to address and tackle these challenges together. These together mean their knowledge base is improved as well as their confidence to carry out these interventions/suggestions.* | | | |
| **CMOC34** | **Context** | **Mechanism** | **Outcome** | **Additional Information & Excerpts from Text** |
|  | Rural, tribal communities with poor health care delivery | ***Resource***: Wider community attends meetings  ***Reasoning***: The community develops ‘critical consciousness,’ awareness of cause and effect linkages for health problems, and how they can influence these linkages | Community supports the interventions and continues to engage in discussions | Fig 4:  *Implementation Methods:* Wider community attend meetings and discuss problems and strategies  Pg 9:  Group members disseminated stories about pregnancy and delivery during community meetings on at least four occasions during the cycle. During the 3-year study period, facilitators and members narrated an estimated 976 new stories. This enabled women and the wider community to **discuss cause and effect linkages**, but also some of the more distal causes of health problems. This is important since one of **the theoretical premises of the intervention is that behaviour change will occur if communities are able to analyse the cause and effect linkages** of health problems, and then define **ways in which they can influence these linkages**. In the final stages of this process, communities would ideally understand both upstream and downstream determinants of health, identify the political and economic roots of ill health, and challenge actors responsible for perpetuating these. Writers such as Freire described this as the development of ‘**critical consciousness’**, or the process through which individuals and groups **become conscious of the oppressive systems** and actors that maintain some in poverty and ill health [19] We suggest that community mobilisation may have begun to catalyze critical consciousness among group members and the wider community, as **evidenced in group members’ support to local village health committees and their involvement of community health workers in discussions** about entitlements to health services. |
|  | *In rural, tribal communities with poor health care delivery (C), wider community meetings to discuss problems and strategies/ (R1) works to develop a ‘critical consciousness’ among the community by building awareness of cause and effect linkages for health problems and how they can influence these linkages (R2). This developed ‘critical consciousness’ drives the community to support the interventions and continue to engage in such discussions (O).* | | | |
| **Relevant IPT(s)** | ***Notes**** | | | |
| Not Applicable | *This sounds a bit like an empowerment theory. By attending the meetings, learning some information, and engaging in these conversations, the wider community basically gains additional awareness/understanding which spurs this ‘critical consciousness’ and recognition of the systems which then spurs further involvement/engagement?* | | | |
| **CMOC35** | **Context** | **Mechanism** | **Outcome** | **Additional Information & Excerpts from Text** |
|  | Rural communities with often marginalized and vulnerable populations | ***Resource***: Group serves as advocates in community and encourage varying community members to attend  ***Reasoning***: Group’s inclusivity means more community members are familiar with and willing to engage with programme | Increased awareness in the community overall | Fig 4:  *Implementation Methods:* Wider community attend meetings and discuss problems and strategies  Pg 9:  The groups’ inclusiveness meant that different community members and decision-makers present during deliveries were likely to have attended meetings and therefore have **increased awareness of maternal and newborn health issues.**  Pg 10:  Group members themselves became active health advocates in the community. |
|  | *In rural communities with often marginalized and vulnerable populations (C), women’s group members can as advocates in the community attend various groups of members to attend meetings (R1). This inclusivity means more community members become familiar with and are willing to engage with the programme (R2) which increases the overall community’s awareness of the health issues (O).* | | | |
| **Relevant IPT(s)** | ***Notes**** | | | |
| Not Applicable | *Not totally sure about this one either. I think ‘inclusivity’ is the important component here but not totally sure if that is the resource or the reasoning. The act of being inclusive means that other community groups (maybe those who have been marginalized, etc.) are being pulled into and engaging in spaces they wouldn’t have been before. So this is increasing their awareness and familiarity with the subject matter discussed at the meetings they are now participating in, which increases the overall community’s awareness. The reasoning and outcome here seem to have a bit of overlap/cyclical effect* | | | |
| **CMOC36** | **Context** | **Mechanism** | **Outcome** | **Additional Information & Excerpts from Text** |
|  | Rural communities with often marginalized and vulnerable populations | ***Resource***: Groups actively involve wider community in discussions around problems/strategies  ***Reasoning***: Community develops a sense of belonging from sharing and discussing these issues | A wide range of community members, including men, become involved and support the programming – such as women’s groups’ solutions for implementation | Fig 4:  *Implementation Methods:* Wider community attend meetings and discuss problems and strategies  Pg 9:  . . . involvement of the wider community, including local community health workers, and the active targeting of marginalised groups and pregnant women. Group members garnered support for maternal and newborn health issues beyond the groups by **actively involving the wider community in discussing their problems and strategies**.  . . . with participatory cycle, groups became open to all community members and men, relatives of pregnant women and frontline government workers were free to attend. Second, members shared their problems and strategies with the wider community during village and cluster-level meetings. Third, community members, including men, offered support in the implementation of the groups’ strategies  Pg 10:  *We used to live on our own, only concerned about our family wellbeing, and others also used to only see their own interest. But now, as we are sharing and discussing our issues, we have developed a sense of bonding with each other and are helping each other in times of need. (Group member, Phase 4 FGD)* |
|  | *In rural communities with often marginalized and vulnerable populations (C), involving the wider community in discussions around problems/strategies (R1) helps to develop a sense of belonging in the community when they share and discuss these issues (R2). This sense of belonging and ‘shared purpose’ means that a wider range of community members, including men, become involved in and support the programming such as implementing the solutions that are discussed (O).* | | | |
| **Relevant IPT(s)** | ***Notes**** | | | |
| Not Applicable | *Sense of belonging (and maybe even ownership?) means that wider group feels ‘spurred’ to action and being involved in the support programmes* | | | |
| **CMOC37** | **Context** | **Mechanism** | **Outcome** | **Additional Information & Excerpts from Text** |
|  | Externally-implemented intervention in an area with previous experience with NGOs and government health interventions  +  Does not requiring structural barriers to be addressed | ***Resource***: Cyclical learning process where community members are not only ‘recipients’ but also ‘designers’/ ‘implementers’  ***Reasoning***: Community members meaningfully contribute to the programme which builds their trust in the programme | Community participates in intervention 🡪 mortality decreases | Pg 11:  . . . mobilisation through groups is not **a discrete intervention where impact is delivered linearly** from implementers to recipients. Instead, implementers, facilitators, group members and community members are all in turn ‘designers’, ‘implementers’ and ‘recipients’ of learning and change. The **recognition that all these participants can and must contribute is critical to trust, and thereby to behavior change**. This **cyclical learning process** is different to methods used in traditional health education or even ‘behaviour change communication’ models, but must be understood and respected by implementing organizations in order to support community mobilisation in the face of multiple local and external challenges.  Pg 12:  This impact may be more difficult to achieve in settings where further mortality reduction is largely dependent on improvements in health service access and quality, in particular emergency obstetric care, but where structural factors hinder communities’ ability to act on these. |
|  | *When an intervention is implemented by an external group in a region with previous experience with such interventions as well as structural barriers to improving health service access and quality (C), a cyclical learning process (R1) where community members are not just ‘recipients’ and can contribute meaningfully to the programme/intervention builds their trust in such programme (R2). As a result, the community participates in this intervention as ‘designers’ and ‘implementers’ as well as ‘recipients,’ and programme aims such as decreased mortality are achieved (O).* | | | |
| **Relevant IPT(s)** | ***Notes**** | | | |
| IPT1/2/3 | *This one is fairly messy…important component in terms of the mechanism here is that it’s a cyclical process and the community is engaged as more than just a passive recipient. In terms of the context, it’s important to note that such interventions can only go ‘so far’ and that if there are more structural challenges that are just not within the power or resources of the community, then engaging or mobilizing them in this way won’t be enough to decrease neonatal mortality.* | | | |

**Paper #6**

| **Author** | **Year** | **Publication Type** | | **Aims/Objectives** | **Setting** |
| --- | --- | --- | --- | --- | --- |
| [Besada et. al](https://www.ncbi.nlm.nih.gov/pmc/articles/PMC5102106/pdf/GHA-9-33507.pdf) | 2016 | Peer-Review Article | | Aim of article is to present strategies to increase male partner involvement in PMTCT | Uganda, DRC, Malawi, Cote d’Ivoire |
| **Study Participants** | **Study design** | **Findings** | | **Description of CE Communications Activities & Who they target** | **Models/Theoretical Frameworks** |
| MoH, implementation partners, district management team, health workers, community members | Qualitative (interviews + FGDs) | Variety of approaches used that were tailored to context. Strategies included community mobilization and sensitization, involving CHWs, and creating male peer cadres. Both positive impacts and unintended negative consequences | | 1) Collaborating with leaders, 2) Use existing CHW cadres, 3) peer support groups, 4) Model clients/couples, 5) Dedicated male/family support groups, 6) Radio messages/Theatre, 7) Male champions/action groups.  Activities focused on male involvement in PMTCT. | N/A |
| **What is interesting about this paper?** | | **Richness Rating** | **Rigour Rating** | **Reference List/Snowballing** | |
| This paper draws on examples from multiple different countries. Describes a number of different activities and how they were conducted – with data coming from interviews – but not as much on the impact | | Moderate |  | None in reference list however could consider reaching out to authors for the original documents used in the document review? May be too long ago and not sure of any confidentiality/IP over the documents | |

| **CMOC38** | **Context** | **Mechanism** | **Outcome** | **Additional Information & Excerpts from Text** |
| --- | --- | --- | --- | --- |
|  | Nomadic communities  and/or  Communities with trusted local leadership structures | ***Resource***: collaboration & training/building capacity of community leaders to convey information to community  ***Reasoning***: community members trust information coming from leaders | Change in population’s attitude regarding content of shared information | Pg 3-5  In Uganda, the **collaboration of village elders** **and community leaders** in the elimination of mother-to-child transmission (eMTCT) of HIV was described as instrumental in **initiating a shift in the population’s attitude** toward the role of men in ANC and eMTCT, and **in gaining trusted access to communities**. In nomadic communities in North-East Uganda, District Health Teams with support from IPs work to engage village leaders, as one IP staff member explained: *We* ***have involved the elders, because we believe they are influential in the communities and they are the ones able to talk to these men and urge them to come for services****. We keep talking to them. (IP, Uganda)*  Similar approaches were described in the DRC and Coˆte d’Ivoire: *The first strategy is* ***lobbying community leaders, including religious leaders****. We* ***strengthened the capacity of those giving messages*** *in the community through HIV training. (Programme National pour la Lutte contre le SIDA (PNLS), DRC) We try to* ***involve the community leaders/chiefs to help them understand what it (PMTCT) is about and what is expected from them****. They* ***take part in the activities, they participate in meetings and convey information*** *to the community. We have a committee for elimination of mother to child transmission. We put this in place in all health areas linked to the project (OHTA) the ASCs (community health workers****), lay counsellors, head of youth, women’s associations, and village chiefs meet each month and plan mass sensitization activities on the importance of ANC****, family practices and male involvement. (Nurse, Coˆte d’Ivoire)* |
|  | *In nomadic communities (C1) with trusted local leadership structures (C2), training community leaders to then convey information to community members (R1) means community members trust the information they are receiving (R2), which leads to changes in attitudes relating to the information (O).* | | | |
| **Relevant IPT(s)** | ***Notes**** | | | |
|  | Kind of about endorsement from leaders? If leader is trained and conveys information for example to promote the involvement of men and they then promote men’s involvement, people’s attitudes change in that direction. Is this conveying that? | | | |
| **CMOC39** | **Context** | **Mechanism** | **Outcome** | **Additional Information & Excerpts from Text** |
|  | Nomadic communities | ***Resource***: Community elders & leaders to convey information to community  ***Reasoning***: Leaders have influential ***access*** to community members which makes them more willing to listen | Change in population’s attitude regarding content of shared information | Pg 3-5  In Uganda, the **collaboration of village elders** **and community leaders** in the elimination of mother-to-child transmission (eMTCT) of HIV was described as instrumental in **initiating a shift in the population’s attitude** toward the role of men in ANC and eMTCT, and **in gaining trusted access to communities**. In nomadic communities in North-East Uganda, District Health Teams with support from IPs work to engage village leaders, as one IP staff member explained: *We* ***have involved the elders, because we believe they are influential in the communities and they are the ones able to talk to these men and urge them to come for services****. We keep talking to them. (IP, Uganda)* |
|  | *In nomadic communities (C1), when community leaders & elders convey information to community members (R1), they have direct access to community members which makes them more willing to listen (R2) and leads to changes in attitudes relating to the information they receive (O).* | | | |
| **Relevant IPT(s)** | ***Notes**** | | | |
|  | Is access an acceptable mechanism? It’s kind of like ‘they have the ear of the people’ – does this come through? | | | |
| **CMOC40** | **Context** | **Mechanism** | **Outcome** | **Additional Information & Excerpts from Text** |
|  | Existing communication structures | ***Resource:*** Conveying locally relevant cultural dynamics in the messaging  ***Reasoning:*** Community members **relate** to the messaging because they see the similarities to their own lives/perceptions | Change in attitude/knowledge relating to messaging | Pg 5  Furthermore in Coˆte d’Ivoire, informants described the **use of radio and local theatre** **to model scenarios of locally relevant cultural dynamics that influence attitudes** to male partner involvement as one district manager described: *We have used local radio sessions to encourage men to come get tested with their wives. For example, there was one program (skit) where a woman tells her husband to get tested and he gets angry in beginning, but then she talks to him and explains the benefits and he changes his attitude in the end. (District Manager, Coˆte d’Ivoire)* |
|  | *In communities with existing communication structures (C), incorporating relevant cultural dynamics in the messaging that’s conveyed (R1) means community members relate to the messaging because they see similarities to their own lives/perceptions/experiences (R2). This enables changes in attitude/knowledge relating to the message (O).* | | | |
| **Relevant IPT(s)** | ***Notes**** | | | |
|  | Relatability of the messaging/programming means community members see themselves in examples/skits 🡪 the concept is in frame of reference/understanding. | | | |
| **CMOC41** | **Context** | **Mechanism** | **Outcome** | **Additional Information & Excerpts from Text** |
|  | Men are head of household/ decision-making as societal norm  Existing decision-making structures in households & communities | ***Resource***: Male local champions convey messaging  ***Reasoning***: Culturally acceptable champions are influential/listened to | Men are accepting of messaging | Pg 5  Alongside collaboration with influential leaders**, community health workers (CHWs), peer supporters, and other community cadres** were trained to sensitize communities, families, and individuals on the importance of male partner involvement as one PMTCT coordinator from Malawi described: ***Culturally male is the head, so if we involve them success would come****. Also local leaders they talk about these issues during their meetings, even for stigma women would be able to take their treatment better. (PMTCT/ART Coordinator, Malawi)* |
|  | *In communities where the societal norm/existing decision-making structure is for men to be the head of household/decision-makers (C), having male local champions convey messaging (R1) is more culturally acceptable and these champions are listened to (R2) which leads to community men accepting their messaging (O).* | | | |
| **Relevant IPT(s)** | ***Notes**** | | | |
|  | Essentially saying men listen to men. | | | |
| **CMOC42** | **Context** | **Mechanism** | **Outcome** | **Additional Information & Excerpts from Text** |
|  | Men are head of household/ decision-making as societal norm | ***Resource***: Male local champions convey messaging  ***Reasoning***: Modelling/desire to be like the ‘ideal’ | Men are accepting of messaging/willing to be more involved | Uganda has a similar program of **peer educators known as male champions** who receive 4 days of training on community sensitization including how to conduct large community dialogue meetings as one international IP respondent explained: *We ask committees to identify male champions. So we’ve tried that out where you know the committees come and say okay we think this is* ***a model man,*** *he looks after his family well, seems to support his wife when she is pregnant and all this kind of things, and we have trained them, taught them how to provide health promotion, how that specific to* ***encouraging fellow men,*** *you know, to be more engaged in sexual reproductive health services. (IP, Uganda)*  In addition to male champions, some men living with HIV serve as expert clients to provide peer support and lead by example in their communities. As one IP explained: *These men are chosen in a way that they have been able to support their wives right from the pregnancy to the HIV-free baby so we can trust them for follow up and education. (IP, Uganda)* |
|  | *In communities where the societal norm is for men to be the head of household/decision-makers (C), having ‘model male’ local champions convey messaging (R1) increases community men’s acceptance of messaging (O) because these champions model ‘ideal’ behaviour that other men want to emulate (R2).* | | | |
| **Relevant IPT(s)** | ***Notes**** | | | |
|  | Think this is a commonly described mechanisms of wanting to model/be like someone else. Read up on additional literature to see how this is phrased. Consider alternatively, same or different from lead by example? | | | |

**Paper #7**

| **Author** | **Year** | **Publication Type** | | **Aims/Objectives** | **Setting** |
| --- | --- | --- | --- | --- | --- |
| [Butler et al.](https://bmchealthservres.biomedcentral.com/articles/10.1186/s12913-020-05394-0) | 2020 | Peer-Review Article | | To describe lessons learned through social accountability project in Malawi (‘Social Accountability for Every Woman Every Child’ to increase accountability around RMNCH targets) | Malawi |
| **Study Participants** | **Study design** | **Findings** | | **Description of CE Communications Activities & Who they target** | **Models/Theoretical Frameworks** |
| Civil society organisations collected data (study participants not clearly stated as the publication team states they did not collect, handle, store, or analyse any of the primary data) | Qualitative (political economy analysis – interviews and group discussions) | “Five key findings:  1) Conducive legal framework for social accountability;  2) Reproductive health policies and other guidelines emphasise accountability and citizen participation;  3) RMNCAH-related national strategies identify social accountability as an important element;  4) Incomplete decentralisation process, with local government systems and structures not fully developed to ensure meaningful citizen participation or oversight of service delivery, and  5) Existing social accountability initiatives are mainly driven by CSOs focusing on community-level mobilisation, with many of them operating independently, duplicating efforts, and with minimal engagement with decision-making structures and actors above health facility level.” | | not very clear…  Community and district bwalos (involving community members, political and administrative actors, and health actors/authorities) | Definition of social accountability: “range of actions and initiatives focused around a communicative relationship between citizens, civil society organisations, service providers and the government, whereby citizens organise both to hold their governments and service providers to account on pre-established norms and to influence the process of formulating new norms and service delivery outputs, usually with the support of other actors such as donors, NGOs, INGOs, the private sector, the media, and government itself.”  ‘tactical’ approaches are bounded, local, micro-level interventions/ tools that are largely limited to ‘society side’ efforts to project voice  ‘strategic’ approaches take a macro view of the process and ‘deploy multiple tactics, encourage enabling environments for collective action for accountability and coordinate citizen voice initiatives with governmental reforms that bolster public sector responsiveness’ |
| **What is interesting about this paper?** | | **Richness Rating** | **Rigour Rating** | **Reference List/Snowballing** | |
| Took a realist approach. Describes a number of intervention strategies, their features, and purposes. Some more logistic ‘steps’/tools/components to consider at local, district, national level. | | Moderate |  | Blake C, Annorbah-Sarpei NA, Bailey C, Ismaila Y, Deganus S, Bosomprah S, Galli F, Clark S. Scorecards and social accountability for improved maternal and newborn health services: a pilot in the Ashanti and Volta regions of Ghana. Int J Gynecol Obstet. 2016;135(3):372–9.  Ganju S, Khanna R, Taparia M and Hardikar N. Promoting Accountability for Maternal Health through Report Card. SAHAJ and ANANDI. 2014. [https://copasah.files.wordpress.com/2014/12/promoting-accountability-for-maternal-health-through-report-card.pdf Accessed 11 Jan 2019](https://copasah.files.wordpress.com/2014/12/promoting-accountability-for-maternal-health-through-report-card.pdf Accessed%2011%20Jan%202019).  Schaaf M, Topp SM, Ngulube M. From favours to entitlements: community voice and action and health service quality in Zambia. Health Policy Plan. 2017;32:847–59.  Papp SA, Gogoi A, Campbell C. Improving maternal health through social accountability: a case study from Orissa, India. Global Public Health. 2013;8(4):449–64. | |

| **CMOC43** | **Context** | **Mechanism** | **Outcome** | **Additional Information & Excerpts from Text** |
| --- | --- | --- | --- | --- |
|  | Existing communication platform is a safe space to communicate between community-level actors + decision-makers (bwalo forums) | ***Resource:*** sharing personal stories and experiences  ***Reasoning:*** Motivation to act | Community/political response to challenges | pg 8:  Communities across the five project districts embraced the bwalo forums, proving highly articulate when provided with a safe space through which to voice their concerns. Having a **personal story to tell about an RMNCAH issue that had affected them directly was found to be the greatest facilitator to community participation in social accountability interventions across the project**, including the bwalos. As an example, in a community bwalo in Dowa District, a close friend of a woman who died in childbirth spoke at a gathering of over 50 people, including local politicians and the media, to attest to how the health system had failed to care for her friend. **The woman’s narrative led to participants offering several, sometimes competing, explanations of why the situation had arisen, and debating the mutual responsibility of health providers and communities to ensure healthy pregnancies and safe deliveries**. The bwalo concluded with the Chair seeking suggestions about how they could ensure mothers arrive at a health facility on time, including the creation of by-laws that both community members and health officials must abide by.  pg 9:  Enablers for positive engagement included motivation to act through sharing personal experiences (community members); |
|  | *In communities with existing communication platforms where varying actors and decision-makers can interact (C), sharing personal stories and experiences (R1) motivates community actors and decision-makers to act (R2) leading to addressing the identified health systems challenges (O).* | | | |
| **Relevant IPT(s)** | ***Notes**** | | | |
|  | Very explicit about motivation as the reasoning here but it’s actually not particularly clear who becomes motivated. | | | |
| **CMOC44** | **Context** | **Mechanism** | **Outcome** | **Additional Information & Excerpts from Text** |
|  | Previous neglect by decision-makers | ***Resource***: Forum for direct interaction (two-way communication) between community and district officials  ***Reasoning***: Community member enthusiasm to voice concerns | Increased knowledge on health system issues among decision-makers and community members | pg 7:  The structure of the bwalo forums **was specific to the context** and, particularly with regards to the emphasis on **the involvement of community-level actors and councilors** (who are key decision-makers in the district local government bodies), informed by the political economy analysis (see Table 2)  pg 10:  It is pertinent to note that the bwalo forums work on all three levels, serving to inform, engage and influence both citizens and state actors, at the same time as facilitating a platform **for two-way communication** between both groups. Other studies have similarly found interface meetings that facilitate bi-directional information sharing to provide an important space for community members to **voice their concerns and ask questions of duty bearers, and for duty bearers to provide information to citizens and respond to citizen’s concerns** in real time [18, 19, 21, 23, 24, 39].  pg 8:  Bwalo participants generally perceived the bwalo, and in particular the district bwalo, to be an **effective method to engage directly with higher-level district officials on issues they felt had been neglected** by politicians in the past. As one community bwalo member concluded, ‘it is difficult for communities to interact with duty bearers on our issues … through the bwalo, the **issues get presented faster’**  pg 9:  **participants of community-level bwalos were enthusiastic about their enhanced knowledge** of RMNCAH issues and the health system more broadly, and were **galvanised by their direct interactions with duty bearers whom they could engage on health-related matters.** |
|  | *In communities where problems have been previously neglected (C), a forum for direct and two-way communication between community actors and district officials (R1) can encourage community members who are enthusiastic to voice their concerns (R2) will increase district officials’ knowledge on health system issues (O).* | | | |
| **Relevant IPT(s)** | ***Notes**** | | | |
|  | Not sure if the reasoning here is encouragement or enthusiasm?? How different are these or can they be combined as one reason/response? | | | |
| **CMOC45** | **Context** | **Mechanism** | **Outcome** | **Additional Information & Excerpts from Text** |
|  | Previous neglect by decision-makers | ***Resource***: Sharing personal experiences  ***Reasoning***: Frustration from lack of action/change | Community members not willing to continue to participate in the forum | pg 9:  Barriers included **frustration associated with sharing personal experiences and time without seeing real change** (community members); |
|  | *In communities where problems have been previously neglected (C), community members sharing personal experiences and stories (R1) that have not been acted upon leads to frustration (R2). This inhibits community members from participating in or contributing to communication forums (O).* | | | |
| **Relevant IPT(s)** | ***Confirm/Refute/ Refine**** | ***Suggested Revisions**** | ***Linkages/Knock-on Effects/Rival Theories**** | ***Notes**** |
|  |  |  |  | Think this one works as is. Neglect + vulnerability in sharing experiences 🡪 lack of action 🡪 frustration 🡪 vicious cycle of then not participating further |
| **CMOC46** | **Context** | **Mechanism** | **Outcome** | **Additional Information & Excerpts from Text** |
|  | Democratic government where freedom of speech is not threatened | ***Resource***: Traditional forum for direct interaction (two-way communication) between community and district officials  ***Reasoning***: Feeling safe and secure to share perspectives in forum setting | Willingness to participate (from both sides) | pg 11:  However, we have been able to identify some of the contextual factors that contributed to enabling the mechanisms described above (bwalo participant responses) to be activated through the experience of the bwalo forums:  1) Cultural contextual elements lent themselves to the effective operationalisation of the bwalo forum, which was based **on a traditional method of dialogue, invoking the concept of an informal local system of discussing and dealing with community issues.**  2) The existing social environment was such that both community members and duty bearers **were willing to come together to participate in this forum.**  3) PACHI is **a trusted and well-connected organisation** in the districts in which bwalos were created.  It is worth noting that sociopolitical contextual factors**, such as type of government and level of free speech, will strongly affect the success of interventions such as the bwalo forum. Malawi’s status as a multiparty democracy and its relative level of free speech and human rights observance in recent years makes it a more favourable environment than some other countries for this type of intervention.** |
|  | *In democratic societies with freedom of speech (C), a traditional communication forum with direct interaction and two-way communication between community actors and district officials (R1) provides a safe space where community members feel safe and secure to share their perspectives (R2). This sense of safety and security leads to both sides being willing to participate in the dialogue (O).* | | | |
| **Relevant IPT(s)** | ***Notes**** | | | |
|  | Interesting component of socio-political/freedom of speech here. I think that is highly connected with safety/security. Additionally the traditional/familiarity component of the intervention (bwalo) would contribute to that safety/security. | | | |
| **CMOC47** | **Context** | **Mechanism** | **Outcome** | **Additional Information & Excerpts from Text** |
|  | Previous neglect by decision-makers | ***Resource***: Forum for direct interaction/ communication between various community members + government actors  ***Reasoning***: Fear of retaliation/reprisal | Health providers not willing to participate in the forum | pg 9:  Barriers included . . . fear of reprisal when speaking out against the government (health providers). |
|  | *In communities where problems have been previously neglected (C), a forum with direct interaction between community and government actors (R1) may cause fear of retaliation/reprisal among health providers (R2). This inhibits participation in the communication forum (O).* | | | |
| **Relevant IPT(s)** | ***Notes**** | | | |
|  | Not positive about the resource here but trying to express that this forum which is a resource in others and serves as a positive opportunity to share opinions can evoke fear of retaliation in a different population. Is it important that this population is health providers specifically? This feels too specific so thought it could be abstracted up. | | | |

**Paper #8**

| **Author** | **Year** | **Publication Type** | | **Aims/Objectives** | **Setting** |
| --- | --- | --- | --- | --- | --- |
| [Dongre et al.](https://link.springer.com/article/10.1007/s12098-009-0028-y) | 2009 | Peer-Review Article | | To elucidate effect of community mobilisation & health education programme on care-seeking. | India |
| **Study Participants** | **Study design** | **Findings** | | **Description of CE Communications Activities & Who they target** | **Models/Theoretical Frameworks** |
| Mothers with children 0-11 months old | Mixed Methods:  Cross-Sectional (Survey) + Qualitative (interviews & FGDs) | Improvement in mothers’ awareness. Care-seeking to government health care facilities significantly declined, however significant improvement in care-seeking at private health care facilities for sick newborns. | | Women’s self-help groups, adolescent girl forums, farmers’ clubs, and village coordination committees (included community’s action experience learning cycle) | PATH (Program for Appropriate Technology for Health) guidelines adopted for development of health education material [11] |
| **What is interesting about this paper?** | | **Richness Rating** | **Rigour Rating** | **Reference List/Snowballing** | |
| discussion around the process of developing the handbook. | | Moderate |  | Howard-Grabman L, Storti C. Demystifying Community Mobilization: An Effective Strategy to Improve Maternal and Newborn Health. U.S. Agency for International development. | |

| **CMOC48** | **Context** | **Mechanism** | **Outcome** | **Additional Information & Excerpts from Text** |
| --- | --- | --- | --- | --- |
|  | Rural community | ***Resource***: Language and pictures adapted and relevant to local context  ***Reasoning***: Messaging was understandable by audience because it was relevant to their experiences, language and frames of reference | Messaging/ information received – increased knowledge | pg 46:  Program staff **developed simple health messages including local words for newborn danger signs and pictures appropriate to local rural context**. The rough draft of health education material containing **hand drawn pictures and handwritten texts messages was presented to a local group of target audience i.e., mothers to test if the pictures and messages conveyed the appropriate meaning. The required modifications in pictures and health messages were made** before finally preparing the handwritten flipbook. The pictures in the final flipbook **were socially and culturally relevant and the messages were written in local language ‘Marathi’ incorporating the local terms**. Subsequently, the photocopies of this flipbook were used by field workers for health message dissemination.  pg 46:  The VCCs were **endorsed by village Gram-panchayat** (local self-government) for implementation and monitoring health care services at village level. VCCs raised health fund from villagers for village level health activities. The capacity of the VCC members was strengthened during their monthly village based meetings to **develop their gender sensitive village health plan with culturally** appropriate solutions to local problems and develop emergency transport plan. The trained social worker facilitated **Community’s Action Experience Learning Cycle (CAELC) through VCCs to explore and collectively act upon their priority maternal and child health issues**. |
|  | *In rural communities (C), using appropriate/simple language and imagery that is adapted to local context (R1) makes the communication more understandable to the audience because it is relevant to their experiences/frames of reference (R2). This allows for the information to be received and increase the knowledge of the audience (O).* | | | |
| **Relevant IPT(s)** | ***Notes**** | | | |
|  | Very similar to high extraction CMOCs on language/appropriate messaging. | | | |

**Paper #9**

| **Author** | **Year** | **Publication Type** | | **Aims/Objectives** | **Setting** |
| --- | --- | --- | --- | --- | --- |
| [Hamal et al.](https://www.ncbi.nlm.nih.gov/pmc/articles/PMC6571494/) | 2019 | Peer-Review Article | | To identify existing social accountability structures and activities in Far-Western Nepal, their functions, activities, and challenges | Nepal |
| **Study Participants** | **Study design** | **Findings** | | **Description of CE Communications Activities & Who they target** | **Models/Theoretical Frameworks** |
| Policy advisors, healthcare officials, healthcare providers, non-governmental staff, women | Qualitative (interviews + FGDs with women) | Mothers groups and community health volunteers focused on information provision, but were considered trusted sources. Challenges centered around lack of mandates and limited capacity | | Range of social accountability structures including mothers groups (women of reproductive age, conduct monthly meetings facilitated by a local FCHV who shares health-related information which the mothers group members then share with community members), female community health volunteers (self-motivated women in the community who are trained on health education and promotion), health facility management committees (largely made up of a range of community representatives and leaders + reps from health facility) as well as activities such as social audits and community score boards. | Conceptual framework: information, dialogue, and negotiation |
| **What is interesting about this paper?** | | **Richness Rating** | **Rigour Rating** | **Reference List/Snowballing** | |
| Fairly interesting paper about social accountability and really focused on the empowerment/capacity/advocacy as well as relationship between patients/women and ‘decision-makers’/those with power. Interesting comments around power, relationships, and even fear. Very little (virtually no) information about context and even intervention/resources that can be extrapolated. | | Moderate |  | Gullo S, Galavotti C, Altman L. A review of CARE’s community score card experience and evidence. *Health Policy Plan.*2016;31(10):1467–1478. doi: 10.1093/heapol/czw064.  Scott K, George AS, Harvey SA. et al. Beyond form and functioning: Understanding how contextual factors influence village health committees in northern India. *PLoS One.*2017;12(8):e0182982. doi: 10.1371/journal.pone.0182982. | |

| **CMOC49** | **Context** | **Mechanism** | **Outcome** | **Additional Information & Excerpts from Text** |
| --- | --- | --- | --- | --- |
|  | health workers in position of power | ***Resource***: Forum to provide feedback on health services  ***Reasoning***: Respect/regard for health workers | No open/honest communication | pg 283:  Some reasons why women did not complain about health services were**: lack of awareness about their health rights, their shyness, and communities’ perception of healthcare providers as respected and important people:** *“Generally, in the rural areas community people are* ***not empowered to complain directly*** *to the health facility* ***because they think those people working in health facilities are busy, very important persons, and they are not supposed to complain****. That is the perception they have in rural areas because they are not aware of their rights”* [Policy Advisor, non-government]. |
|  | *In communities where health workers are perceived to be in positions of power (C), community members do not want to disrespect this position by providing potentially negative feedback (R2), resulting in lack of reporting negative feedback on health services (O).* | | | |
| **Relevant IPT(s)** | ***Notes**** | | | |
|  | Not sure how to express the resource here…not a specific intervention or action happening, question being asked is specific to the research being conducted. Is it okay to not have a resource? | | | |
| **CMOC50** | **Context** | **Mechanism** | **Outcome** | **Additional Information & Excerpts from Text** |
|  | lack of awareness on health rights | ***Resource***: Forum to provide feedback on health services  ***Reasoning***: not empowered | No open/honest communication | pg 283:  Some reasons why women did not complain about health services were**: lack of awareness about their health rights, their shyness, and communities’ perception of healthcare providers as respected and important people:** *“Generally, in the rural areas community people are* ***not empowered to complain directly*** *to the health facility* ***because they think those people working in health facilities are busy, very important persons, and they are not supposed to complain****. That is the perception they have in rural areas because they are not aware of their rights”* [Policy Advisor, non-government]. |
|  | *In communities there is limited health rights awareness (C), communities are not empowered to communicate directly with the health facility (R2). This leads to a lack of feedback and accountability from the health services (O).* | | | |
| **Relevant IPT(s)** | ***Notes**** | | | |
|  | Not sure how to express the resource here…not a specific intervention or action happening, question being asked is specific to the research being conducted. Is it okay to not have a resource? | | | |
| **CMOC51** | **Context** | **Mechanism** | **Outcome** | **Additional Information & Excerpts from Text** |
|  | District-level planning for health management | ***Resource:*** Health post volunteers serve as one of the first contacts for community members  ***Reasoning:*** Fear of reprisal/angering the healthcare provider if they told them directly | Communicating concerns to healthcare providers through these intermediary groups | pg 282:  Within a district, health posts are the first point of contact at the village level (Village Development Committee, VDC), followed by primary healthcare centres and health centres at the sub-district level, and district hospitals. Health posts extend community-based health activities through female community health volunteers (FCHVs) at the *ward* level (9 wards make 1 village) and outreach clinics  pg 283:  Women in our study areas often mentioned **communicating their maternal health concerns directly to the relevant healthcare providers. In most cases such communications were related to maternal health problems rather than complaints/concerns about maternal health services**. Women mainly feared of reprisals if they complained directly to the healthcare providers. “People **fear to complain because they think we might get angry when we hear about our complaints**. They tell everything to FCHV and request them not to disclose their names” [Healthcare provider, Kailali].  pg 283-284:  As an alternative, **women used intermediaries such as mothers’ groups (MGs),** FCHVs, Health Facility Operation and Management Committee (HFOMC) members to voice their concerns to the health sector, and they participated in activities such as social audits or CHSB. Box 1 provides an overview of these intermediaries, and Figure 2 visualizes the village/ward-level organization of MGs, FCHVs, and the HFOMCs. |
|  | *In communities where health is managed at the district-level (C), health volunteers serving as a primary contact point for community members (R1) provide an avenue/intermediary group for individuals to communicate concerns they would be unable to share otherwise (O) due to fear of angering their healthcare providers if they were to share these concerns/feedback directly (R2).* | | | |
| **Relevant IPT(s)** | ***Notes**** | | | |
|  | Context is a bit challenging but I think the aspect that decisions and organisation happen at the district level is how these health volunteers/subsidiaries function as a connection between community and provider. | | | |
| **CMOC52** | **Context** | **Mechanism** | **Outcome** | **Additional Information & Excerpts from Text** |
|  | District-level planning for health management | ***Resource***: Community members have a relationship with the healthcare provider  ***Reasoning***: This familiarity and closeness allows them to feel comfortable with the provider | Complain directly to healthcare providers | pg 283:  In contrast, a healthcare official (Kailali) explained that **the existence of local healthcare providers and the community’s familiarity and closeness to them enabled people to make direct complaints to the healthcare providers**.  pg 289:  These activities helped to resolve problems with the health services locally, often with little or no external support. They also helped to build trust and strengthen the relationship between the community and service providers and generate responsiveness from the service providers. [16,18,40,41] |
|  | *In communities where health is managed at the district-level(C), the relationship between community members and healthcare providers (R1) encourages community members to provide feedback/complain directly to healthcare providers (O) because they feel a familiarity and closeness to the healthcare provider that allows them to feel comfortable to share feedback directly (R2).* | | | |
| **Relevant IPT(s)** | ***Notes**** | | | |
|  | Context similar to previous CMOC because in some ways this is a rival theory maybe? Or maybe not a rival theory but the opposite facts of what previous CMOC is saying. | | | |
| **CMOC53** | **Context** | **Mechanism** | **Outcome** | **Additional Information & Excerpts from Text** |
|  | High staff turnover | ***Resource***: Lack of relationship/trust built between healthcare provider and patients/community  ***Reasoning***: means they don’t feel safe | No open communication | pg 283:  Another healthcare provider (Kailali) explained that **the high staff turnover prevents women from building a relationship of trust with healthcare workers, which meant that they often do not feel safe when complaining to the healthcare provider**. |
|  | *In health settings with high staff turnover (C), community members do not build a relationship and trust with the healthcare providers (R1) which means they do not feel safe to share their feedback (R2) and there is a lack of open/honest communication between patients/community and providers (O).* | | | |
| **Relevant IPT(s)** | ***Notes**** | | | |
|  | High staff turnover could be context or resource???? | | | |
| **CMOC54** | **Context** | **Mechanism** | **Outcome** | **Additional Information & Excerpts from Text** |
|  | District-level planning for health management | ***Resource***: Intermediary groups serve as link between community and health sector  ***Reasoning***: familiarity/comfort with these intermediary groups allows for sharing problems | Complaints about health-related matters reach healthcare providers | pg 284:  FCHVs and MGs communicated the concerns of women to the health sector. Women **discussed and shared their maternal health problems in the MG meetings**, including their health service experiences and concerns. In these meetings, FCHVs provided information regarding maternal and child health and health services. **FCHVs play a major role in linking communities with the health sector** and in convincing them to use the health services. FCHVs were described as **mediators** between the communities and the health facilities, who also communicated information on women’s concerns about the health services to the health sector**. Almost all healthcare providers and women mentioned FCHVs as their first point of contact to communicate their concerns and complaints about health-related matters,** such as inappropriate behaviours of healthcare professionals. Two healthcare officials (Kailali) explained that FCHVs, being female, **were convenient persons for women to share their problems with. *“It is easier for ladies to share their problems with ladies than to gents. And then FCHVs tell that (problems) to us.”*** |
|  | *In communities where health is managed at the district-level (C), intermediary groups serve as a link between community members and the health sector (R1) and share honest feedback/complaints with healthcare providers (O). This is because community members feel a sense of familiarity and comfort with these intermediary groups that allows them to feel comfortable sharing these concerns/feedback directly (R2).* | | | |
| **Relevant IPT(s)** | ***Notes**** | | | |
|  | Maybe get rid of this one…not very clear? Quite similar to CMOC50 | | | |
| **CMOC55** | **Context** | **Mechanism** | **Outcome** | **Additional Information & Excerpts from Text** |
|  | District-level planning for health management | ***Resource***: Social audit process  ***Reasoning***: Increased communities’ awareness of services + healthcare providers understanding of community needs | Improved quality of service | pg 285:  Perceived changes – Women from Kailali district felt that their complaints, mainly related to healthcare providers’ behaviour, were addressed when they were communicated. *“Now with those coming to get the services, they are not talking rudely, that has improved”* [Women, Non-hill, Kailali]. Healthcare officials and providers from both terai and hills of Kailali district also mentioned that the complaints were acted upon.  pg 286:  Perceived changes – Respondents explained that the social audits **increased the communities’ awareness and interest in local health services.** The communities’ **concerns and interests helped healthcare providers to understand the needs of the community, thereby improving the quality of the services**. *“It has been helping a lot. All complaints they express during social audit (..) it helps us to stay aware. The behaviours we do unconsciously when we hear from public, we also get chance to improve that (behaviour). (..) In some cases I also apologised because of the complaints (..)”* [Healthcare Provider, Kailali].  Two healthcare providers (Kailali) appreciated the effectiveness of the social audit in terms of **learning and improving and ensuring accountability of the health sector actors. Women in 2 sites (Doti) felt that when they shared their problems during social audits, some of them were addressed. These changes only concerned local-level actions.** Other healthcare providers, women or respondents never mentioned about effectiveness of social audits. |
|  | *In communities where health is managed at the district-level (C), the social audit process (R1) increases the awareness of services and understanding of needs (R2) leading to improved service quality (O).* | | | |
| **Relevant IPT(s)** | ***Notes**** | | | |
|  | This may not be a CMOC? Can increased awareness and understanding be reasoning? | | | |
| **CMOC56** | **Context** | **Mechanism** | **Outcome** | **Additional Information & Excerpts from Text** |
|  | Marginalised populations | ***Resource***: Social audits  ***Reasoning***: shy/cannot speak up because of power dynamics between women and health sector | Lack of participation/ incorporation of these perspectives | pg 287:  Secondly, local women in some study sites did not know about the audits. Thirdly, a woman (Doti) and a policy advisor (non-government) mentioned that **participation was limited in the audits, especially by women from marginalized castes, either in terms of attendance or in terms of expressing themselves. “*Women here cannot speak up, they are shy, (..) women here cannot speak up in front of all”*** [Women, Doti].  The NGO facilitators were reported to ensure that everybody engaged in the entire process of the dialogue and negotiation. “*We enable them to speak. We have to facilitate, otherwise they do not speak. Those who are from the marginalized, socially secluded groups, they hesitate to speak in such meetings”* [NGO staff].  pg 289:  The role of an external facilitator was particularly crucial in mediating power relations between the women (less powerful) and the health sector (the more powerful). |
|  | *In communities with marginalised populations (C), social audits/forums for communication (R1) may not achieve significant participation (O) because these participants are unable/become shy to share their perspectives due to the power imbalance between them and present authorities (R2).* | | | |
| **Relevant IPT(s)** | ***Notes**** | | | |
|  | Potential rival here in terms of the facilitators feeling this isn’t a challenge?? | | | |
| **CMOC57** | **Context** | **Mechanism** | **Outcome** | **Additional Information & Excerpts from Text** |
|  | Social audit programme implemented (health score boards) | ***Resource:*** Healthcare providers demonstrate increased concern for patients  ***Reasoning:*** Women trust healthcare providers | Increased use in maternal health services | pg 287:  NGO staff explained that the **health worker’s increased concern about women and their children’s health and the feeling that the health facilities are there for them improved women’s acceptance of and trust in the health services**. They claimed that this trust also contributed to an increase in the use of maternal health services, such as institutional deliveries and antenatal and postnatal check-ups, as confirmed by the score boards. |
|  | *In communities where a social audit/community engagement programme has been implemented (C) and led to healthcare providers being perceived as increasingly concerned for women/their patients (R1), women/patients trust the health services due to this concern (R2) leading to increased use of maternal health services (O).* | | | |
| **Relevant IPT(s)** | ***Notes**** | | | |
|  | This one is interesting and could potentially be nested because it sounds like the programme being implemented made a change in the healthcare providers, which was this increase in concern for patients, which in turn increased the patients trust in the providers. | | | |
| **CMOC58** | **Context** | **Mechanism** | **Outcome** | **Additional Information & Excerpts from Text** |
|  | Public avenues of accountability | ***Resource:*** holding actors accountable to follow up on action/promises of better performance  ***Reasoning:*** Fear of repercussions of public exposure of poor performance | Healthcare providers feel accountability/ responsibility to follow through | pg 288-289:  Both structures and activities generated enforceability through **informal sanctions, such as fear of repercussions among health-sector actors,** improved **monitoring and follow-up of women’s issues communicated to the health sector by healthcare officials and HFOMC members**, albeit to a limited extent. This is in line with other studies, for instance, Lodenstein et al and Brinkerhoff explained **that negative publicity or public exposure of poor performance in public forums creates incentives among public sector actors to respond positively because of a fear of repercussions**. [13,36] Involvement of the media in such events is likely to increase publicity about the problem, amplifying demands and spurring attention and follow-up for the public sector response. [10,17,37] |
|  | *In programmes with public avenues for accountability (i.e. media involvement), using these forums as avenues for actors to follow up on actions/promises (R1) will improve healthcare providers accountability to follow through (O) because of their fear of repercussions of public exposure of poor performance (R2).* | | | |
| **Relevant IPT(s)** | ***Notes**** | | | |
|  | Another strange one that not quire how to express and maybe doesn’t belong here? The context is more about the programme/intervention rather than the community | | | |

**Paper #10**

| **Author** | **Year** | **Publication Type** | | **Aims/Objectives** | **Setting** |
| --- | --- | --- | --- | --- | --- |
| [Morrison et al.](https://www.ncbi.nlm.nih.gov/pmc/articles/PMC5104268/) | 2010 | Peer-Review Article | | To describe how women’s groups using participatory learning and action can improve maternal and newborn survival and the factors influencing their effectiveness | Nepal |
| **Study Participants** | **Study design** | **Findings** | | **Description of CE Communications Activities & Who they target** | **Models/Theoretical Frameworks** |
| Women’s group members, facilitators, health volunteers, community leaders, local men, and women (who do not attend women’s groups) | Qualitative (interviews, FGDs with photo-elicitation) | Women’s groups impacted health outcomes through: learning about health, developing confidence, disseminating information to the wider communities, and building community capacity for action | | Women’s groups (PLA method) | Same Makwanpur study that may come up in other papers [based on Bolivia Warmi project] |
| **What is interesting about this paper?** | | **Richness Rating** | **Rigour Rating** | **Reference List/Snowballing** | |
| Describes four mechanisms to explain women’s group effect on health outcomes (however these do not translate into realist mechanisms). Table 4 provides detail on contextual info. Number of references to other literature from a theoretical perspective that may be useful – some similarities/parallelism to other included articles referring to critical consciousness. Very little contextual information, but they do acknowledge contextual challenge of political unrest.  Additional components that were not elaborated on but could support other CMOCs: “the trial team were committed to taking a participatory approach, gained the confidence of communities, disseminated knowledge about maternal and neonatal health, and were perceived as knowledgeable, trustworthy, respected individuals by communities. Communities in intervention areas were also positive and responsive to NGOs, which may have affected the receipt of and response to the intervention. The intervention was feasible and acceptable as maternal and neonatal health was perceived to be the domain of women, and community groups were an accepted way to gain knowledge, personally develop, and help the community progress.” | | Moderate |  | Morrison J, Tamang S, Mesko N, Osrin D, Shrestha B, Manandhar M, et al. Women’s health groups to improve perinatal care in rural Nepal. *BMC Pregnancy Childbirth.*2005;5:6  Howard Grabman L. Planning together: developing community plans to address priority maternal and neonatal health problems in rural Bolivia. In: De Koning K, Martin M, editors. *Participatory research in health issues and experiences.* London: Zed Books; 1996. | |

| **CMOC59** | **Context** | **Mechanism** | **Outcome** | **Additional Information & Excerpts from Text** |
| --- | --- | --- | --- | --- |
|  | Rural communities, far from road/health facility | ***Resource***: Sharing information and experiences [at women’s groups]  ***Reasoning***: Support | Cross learning & Increased knowledge/health awareness | pg 5-6:  3.2 Mechanisms: Learning about health—  Participants explained that the intervention had **enabled learning** by women’s group members: *‘This group is very good,* ***I can hear new things, I can meet with friends… we have learned lots of things after joining the group’*** (women’s group member, FGD).  The women’s groups were felt to be **a source of support, and a place for learning and sharing knowledge**: *‘I wish to learn, that is why I come (to the group). Otherwise, we didn’t know before that we could get medicine from the health post’* (women’s group member, FGD).  **Group facilitators also felt they had learned and personally developed**: ‘*We have got the chance to learn, as do group members. Before, I only knew that I should be clean, but I didn’t know that the baby should also be clean. At that time I was unaware. Anyway, for this third baby, because of the training provided (by MIRA), I did whatever I have learnt. I think I have also changed’* (facilitator, interview).  Many respondents believed that persons who travelled beyond the immediate village were more likely to have new and different knowledge, and this knowledge should be shared with others*: ‘(Women) should come out of the house and learn things. Whatever we know, we should tell others. We need to share with each other.’* (community leader, interview). |
|  | *In rural communities far from health facilities (C), peers sharing information/experiences (R1) in an environment that allows individuals to feel supported (R2) leads to cross-learning & increased knowledge/health awareness (O).* | | | |
| **Relevant IPT(s)** | ***Notes**** | | | |
|  | Check previous Rath CMOCs to better explain the reasoning? | | | |
| **CMOC60** | **Context** | **Mechanism** | **Outcome** | **Additional Information & Excerpts from Text** |
|  | Rural communities | ***Resource:*** Sharing experiences and discussions with peers  ***Reasoning:*** Women develop confidence | Decrease in embarrassment allowing for more open conversations and discussion | pg 6:  3.2 Mechanisms: Confidence—  The **confidence of group members developed through their involvement with the intervention, and reticence decreased**: *‘I liked their interaction very much… one or two women were speaking openly… while going to the meeting, I have seen an improvement in women’s consciousness’* (community leader, interview).  The **lessening of laaj, a sense of embarrassment or shame**, was mentioned repeatedly: *‘They were eager to know about things and they were not hesitating to discuss their problems. Those women, who didn’t want to talk about their problems before, were interacting with each other’* (community leader, interview). A facilitator commented: *‘The women of this village didn’t talk about pregnancy because they felt embarrassed. They carried heavy loads and bleeding occurred… Now, they meet with us and ask us for help when this happens. They ask without feeling shy’* (facilitator, interview).  Group **discussion and implementation of strategies to address maternal and newborn health problems helped develop confidence,** (an example being the management of money through maternal and child health funds*): ‘Before (we went to the group), we couldn’t understand and we couldn’t speak properly. Now… if we go to meetings then we can manage things properly’* (women’s group member, FGD). |
|  | *In rural communities (C), sharing information/experiences with peers (R1) develops women’s confidence (R2), leading to a more open conversations without any embarrassment (O1) which allows for more openness in discussions on challenges/experiences (O2).* | | | |
| **Relevant IPT(s)** | ***Notes**** | | | |
|  | Confidence is an interesting reasoning/response 🡪 decrease in embarrassment could also be the reasoning? But this enables/opens up future conversation | | | |
| **CMOC61** | **Context** | **Mechanism** | **Outcome** | **Additional Information & Excerpts from Text** |
|  | Rural communities | ***Resource***: Knowledge and supportive group environment  ***Reasoning***: Confidence in knowledge | Acting on this confidence by challenging household norms, supporting each other, and disseminating information further | pg 6:  There was also some evidence that **group members challenged household norms,** for example when a local girl became pregnant to a boy of another caste, out of wedlock, the group were concerned about how she was being treated*: ‘They were intercaste, the (mother of the boy) didn’t want to let her into the family, and wanted her to abort the baby… all the group members decided to go into her house… now (the girl) says that the (mother) doesn’t scold her’* (facilitator, interview).  pg 6-7  3.2 Mechanisms: Spreading information about good practice—  **Increased knowledge, confidence and encouragement from facilitators stimulated group members to disseminate information** about maternal and neonatal health to other women: *‘If the baby doesn’t suck milk, or breathe, or cry it should be taken to the hospital immediately. We tell (women) everything. Now we are giving training to others’* (women’s group member, FGD).  **Community meetings organised by groups and facilitators also increased awareness about maternal and newborn health**: *‘They ask “what did you do in the meeting, what did you learn by going to the meeting?” We conducted a ward meeting to make other people understand’* (women’s group member, FGD).  Groups initiated several strategies to address health problems, including stretcher schemes, revolving funds for obstetric or newborn emergencies, the making and distribution of clean home delivery kits, and the use of picture card games to discuss the causes, prevention and treatment of maternal and newborn problems (Figure 2a–d). Community planning and implementation of strategies to address prioritised problems also enabled dissemination of information. For example, whilst selling clean home delivery kits made by the group, members told families how to maintain cleanliness at birth: *‘I give them suggestions, saying “if the delivery kit is used then you won’t get caught by diseases”*’ (women’s group member, FGD) |
|  | *In rural communities (C), increased knowledge from a supportive group environment (R1) builds women’s confidence in their knowledge (R2) and empowers them to disseminate their knowledge further (O).* | | | |
| **Relevant IPT(s)** | ***Notes**** | | | |
|  |  | | | |
| **CMOC62** | **Context** | **Mechanism** | **Outcome** | **Additional Information & Excerpts from Text** |
|  | Rural community | ***Resource***: Participatory approaches to learning [PLA cycle]  ***Reasoning***: Develop a critical consciousness | Community empowered to make changes | pg 9:  The **way that women learnt, especially through the picture card discussions, stimulated group members to disseminate information** about maternal and neonatal health to other women informally. Community planning and implementation of strategies to address high priority problems **also enabled dissemination of information through mobilisation of community resources and social networks.** For example, resources were collected for revolving funds and clean delivery kits, which focused the community on maternal and newborn health, and increased community capacity to address problems**. PLA approaches propose that a collective critical consciousness and owned knowledge develops through participation, which enables individuals and communities to become empowered to work towards social change.30,31** Our findings support Freire’s theories of learning and empowerment through building a critical consciousness.9 Other authors have suggested that **involvement with community based organisations and increased social networks empowers communities, and leads to better health outcomes**.32–36 The group process may produce broader understanding of health problems, long-lasting empowerment and development benefit for local communities**. The progression through learning, gathering information, gaining self-confidence, developing skills, organising to take action, and taking action, is more empowering than simply receiving messages.** Empowered community-based organisations are thought to be the essential link between empowered individuals and effective political action. **This dynamic continuum progresses through individual empowerment, small group participation, community organisation and partnership to political action**.37 The **intervention encouraged mobilisation of resources, and groups have linked with their communities to promote newborn and maternal health. Increased access to social networks may have built capacity of communities to work together in addressing problems.** |
|  | *In rural communities (C), a participatory approach to learning in a group environment (R1) develops ‘critical consciousness’ (R2) empowering the community to make changes (O).* | | | |
| **Relevant IPT(s)** | ***Notes**** | | | |
|  | Connect with other CMOCs on critical consciousness. | | | |
| **CMOC63** | **Context** | **Mechanism** | **Outcome** | **Additional Information & Excerpts from Text** |
|  | Political instability [insurgency/civil unrest] | ***Resource***: Not engaging with broader socio-political structures  ***Reasoning***: power dynamics/structures | Changes were small-scale or not sustainable | pg 10:  The intervention could also be criticised because **women’s groups did not appear to engage with broader political and social power structures to address social inequality at a higher level.** The primary health care movement was also often criticised for achieving small-scale successes, but failing to tackle fundamental national power differentials.38 Yet the **political context of the intervention, during an insurgency, was a severe constraint to broader political action**. This trial was conducted during a time of civil unrest when there had been dissolution of local and national democratic structures. In a more stable political context, groups might stimulate interaction with power structures.39–41  Women’s groups offer a sustainable and scalable approach to improve child survival. For scale-up, the management of interventions must be effective. The implementing organisation was clearly important in the success of the intervention: **the trial team were committed to taking a participatory approach, gained the confidence of communities, disseminated knowledge about maternal and neonatal health, and were perceived as knowledgeable, trustworthy, respected individuals by communities**. Communities in intervention areas were also positive and responsive to NGOs, which may have affected the receipt of and response to the intervention. The intervention was feasible and acceptable as maternal and neonatal health was perceived to be the domain of women, and community groups were an accepted way to gain knowledge, personally develop, and help the community progress. |
|  | *In settings of political instability (C), not engaging with these broader socio-political structures (R1) means power dynamics/structures are not incorporated in a sustainable way (R2) which inhibits the long-term nature of any potential changes (O).* | | | |
| **Relevant IPT(s)** | ***Notes**** | | | |
|  | Not positive if this resource/reasoning makes sense. | | | |

**Paper #11**

| **Author** | **Year** | **Publication Type** | | **Aims/Objectives** | **Setting** |
| --- | --- | --- | --- | --- | --- |
| [Prata et al.](https://www.sciencedirect.com/science/article/pii/S0277953612000512?via%3Dihub) | 2012 | Peer-Review Article | | To demonstrate the importance of community mobilisation in the uptake of a community-based misoprostol distribution intervention. | Nigeria |
| **Study Participants** | **Study design** | **Findings** | | **Description of CE Communications Activities & Who they target** | **Models/Theoretical Frameworks** |
| Women (postpartum) | Cross-Sectional (‘Interviews’ + observational quantitative data collected re: misoprostol distribution) | Mostly about increasing information and traditional birth assistants (TBAs) and (community oriented resource persons) CORPs as source of information as well as increased availability of misoprostol. | | Three-pronged community mobilisation effort:  1) Women and communities educated about birth preparedness and PPH in community dialogues, dramas, and print materials  2) CORPs & TBAs trained to counsel women in individual and group education sessions  3) Community members trained as drug keepers to store and dispense misoprostol | N/A |
| **What is interesting about this paper?** | | **Richness Rating** | **Rigour Rating** | **Reference List/Snowballing** | |
| Lots of detail on context and three-pronged community mobilisation approach. Useful theory references in the discussion. Specific discussions and reference to communication theory!! | | Moderate |  | ***THIS PAPER HAS A LOT OF REFERENCES TO MORE GENERAL THEORY/COMMS – INCLUDING:***  J. Finnegan, K. Viswanath. **Communication theory and health behavior change: the media studies framework.** K. Glanz, B. Rimer, F. Lewis (Eds.), Health behavior and health education: Theory, research, and practice (2008), pp. 363-388  L. Ackerson, K. Viswanath. **The social context of interpersonal communication and health.** Journal of Health Communication, 14 (2009), pp. 5-17.  ***ABOUT ADAPTING MESSAGING:***  A. Oenema, F. Tan, J. Brug. **Short-term efficacy of a web-based computer-tailored nutrition intervention: main effects and mediators.** Annals of Behavioral Medicine, 29 (1) (2005), pp. 54-63  R. Petty, J. Bardeb, S. Wheeler. **The elaboration likelihood model of persuasion: developing health promotions for sustained behavioral change.** R. DiClemente, R. Crosby, M. Kegler (Eds.), Emerging theories in health promotion practice and research, Jossey Bass, San Francisco (2009), pp. 185-214  N. Wallerstein. **What is the evidence on effectiveness of empowerment to improve health?** Health evidence network report, WHO Regional Office for Europe, Copenhagen (2006). | |

| **CMOC64** | **Context** | **Mechanism** | **Outcome** | **Additional Information & Excerpts from Text** |
| --- | --- | --- | --- | --- |
|  | Strong sense of cultural, religious, and sociopolitical identities | ***Resource***: Incorporating local knowledge to tailor messaging  ***Reasoning***: Culturally relevant messaging and visual representations are easy to relate & understand | Increased knowledge | pg 1290:  **Community members’ lay knowledge helped inform aspects of the intervention, including the dialogs that were designed to create awareness on the postpartum blood loss**.  pg 1293:  The effectiveness of even relatively low levels of community participation in improving the **tailoring of the intervention in terms of cultural and local relevance** – factors that may be of critical importance to uptake and effectiveness (Wallerstein, 2006) – was demonstrated in this study. For example, when community members learned that 500 ml of blood loss was the threshold for postpartum hemorrhage, they identified the moda, a local rubber cup used for fetching water from the water pot, which holds exactly 500 ml of water, as a useful volume reference. **This provided clear visual representations for the community to understand when a woman had lost too much blood after delivery and was facing a life-threatening emergency.**  Similarly, a community midwife who helped facilitate educational sessions used a local garden egg, yallon bello, which is about the size and shape of a normal uterus, along with balloons to demonstrate how a contracted uterus should appear, and the effects of repeated childbirth on the uterus and uterine atony.  Finally, based on community recommendations and brainstorming with community leaders, TBAs and CORPs during monitoring visits and project debriefings, culturally appropriate items were identified and used to advertise misoprostol use. These items included hijabs (head scarves) for Muslim women, head ties for Christian women and butas (water kettles for ablutions) for Muslim men, and each item was inscribed with the simple message, *‘Take three tablets of misoprostol immediately after birth to prevent postpartum hemorrhage.’*  During subsequent monitoring visits**, these items, developed based on community insights, were frequently cited as having helped disseminate information about postpartum hemorrhage prevention and misoprostol as part of the broader educational campaign.**  pg 1294:  In our study, **use of local similes recommended by community members (e.g., a garden egg to illustrate the size of the normal uterus and a common 500 ml cup to illustrate the blood loss threshold for PPH) proved effective in helping increase the relevance of the messages being communicated.** Incorporating other community recommendations into the intervention (e.g., inscribing educational messages on hijabs and other culturally appropriate and desirable items) **also helped with information tailoring and dissemination, which in turn may contribute to intervention uptake** (Finnegan & Viswanath, 2008; Wallerstein, 2006). **Outside researchers would have been unable to judge by themselves whether inscribing an advertisement on religious items would be acceptable and thus, benefited greatly from the community members’ insight. Finally, such incorporation of community suggestions is important in demonstrating that outside researchers value and take seriously the lay knowledge and opinions of community members** (Cargo & Mercer, 2008; Minkler & Wallerstein, 2008; Mosavel, Simon, van Stade, & Buchbinder, 2005)  Numerous studies have suggested that **attention to social context within which community participation takes place is critical in improving and adapting information, education and communication campaigns for maximum dissemination and uptake** (Finnegan & Viswanath, 2008; Minkler, Garcia, Williams, LoPresti, & Lilly, 2010; Wallerstein, 2006). Grounding interventions in the **lived experience of community members has further been shown to increase the acceptability and effectiveness of the interventions themselves** (Altman, 1995; Salvatore et al., 2009). Furthermore, **tailoring messages to participants in ways that take into account their social norms and other factors** have been shown to be more effective than generic messages (Oenema, Tan, & Brug, 2005; Petty, Bardeb, & Wheeler, 2009) in facilitating understanding and uptake. |
|  | *In communities with a strong social, political, and cultural identity (C), incorporating local knowledge to tailor messaging and using relatable visual representations (R1) makes the message culturally relevant and easy for community members to relate & understand (R2). This increases their knowledge regarding the presented information (O).* | | | |
| **Relevant IPT(s)** | ***Notes**** | | | |
|  | Context similar to other CMOCs from high set. Really good/concrete examples of what the intervention incorporated from ‘lay knowledge.’/relatability examples. | | | |
| **CMOC65** | **Context** | **Mechanism** | **Outcome** | **Additional Information & Excerpts from Text** |
|  | Disparate population with hierarchical social and political structure – information reaches people in different ways | ***Resource***: Multiple avenues of communication  ***Reasoning***: Improved flow of information increases exposure | Knowledge increased | pg 1291:  **Cassette tapes of dramas and printed, illustrated materials were also widely disseminated through various channels**. The key messages delivered through the multi-pronged community mobilization campaign included information about bleeding after delivery, the importance of delivery in a health facility, and information related to misoprostol including use, dose, route, timing and side effects.  pg 1293:  The **multi-faceted approach to diffusing information seemed particularly useful in that different actors played a role in informing pregnant women about bleeding after childbirth and about misoprostol**. This is consistent with a wealth of literature from the communications field **indicating that increasing the number and variety of channels through which health information on a given topic is received improves the flow of information** (Finnegan & Viswanath, 2008), which in turn may help facilitate behavior change. Communications theory also suggests the particular importance of social context and interpersonal communication in helping facilitate changes in health behavior (Ackerson & Viswanath, 2009). In the present study, postpartum interviews found that two of the major groups mobilized in the intervention, **TBAs and CORPs, proved to be the most important sources of information on misoprostol** |
|  | *In disparate communities (C1) with socio-political hierarchies (C2), using multiple avenues of communication (R1) improves the flow of information and increases community members’ exposure to the messaging (R2). This increases their knowledge regarding the presented information (O).* | | | |
| **Relevant IPT(s)** | ***Notes**** | | | |
|  | Not totally sure how to explain this context but it’s the idea that because there is such a strong sociopolitical hierarchical structure and also the examples of communities where women have to stay home…there is a need for excess exposure/multiple avenues of comms in order to reach populations. Maybe this should be the other Context re: social/political identity?  Also can exposure be a reasoning? May be able to connect this to one of the high paper CMOCs | | | |
| **CMOC66** | **Context** | **Mechanism** | **Outcome** | **Additional Information & Excerpts from Text** |
|  | Existing hierarchical social and political structure | ***Resource***: Leaders consulted and involved in leading community dialogues  ***Reasoning***: Community members feel their voices are heard | Community-wide agreement on project implementation | pg 1293:  Community **dialogs** were arranged by each individual community leader in collaboration with CORPs and TBAs of the respective communities and in coordination with the research team. These dialogs took **the form of town hall meetings** and were concentrated toward the beginning of the project. Communities determined the number of total meetings, but they ranged from 3 to 4 town hall events per community. **Community leaders** **ran these dialogs until they felt the concerns of the community had been voiced and agreement on project implementation reached**. Later**, town hall meetings transitioned into opportunities for other smaller group activities, such as interpersonal communications and exposure to other forms of information, including drama groups and printed materials.** |
|  | *In communities with existing hierarchical social and political structures (C), consulting leaders and involving them in reciprocal communication platforms (R1) allows community members to feel their voices are heard because they trust their leaders (R2). The resulting discussion enable community-wide agreement on project implementation (O).* | | | |
| **Relevant IPT(s)** | ***Notes**** | | | |
|  |  | | | |

# Supplementary File 4: Expert advisory committee guided feedback form for CMOCs

**
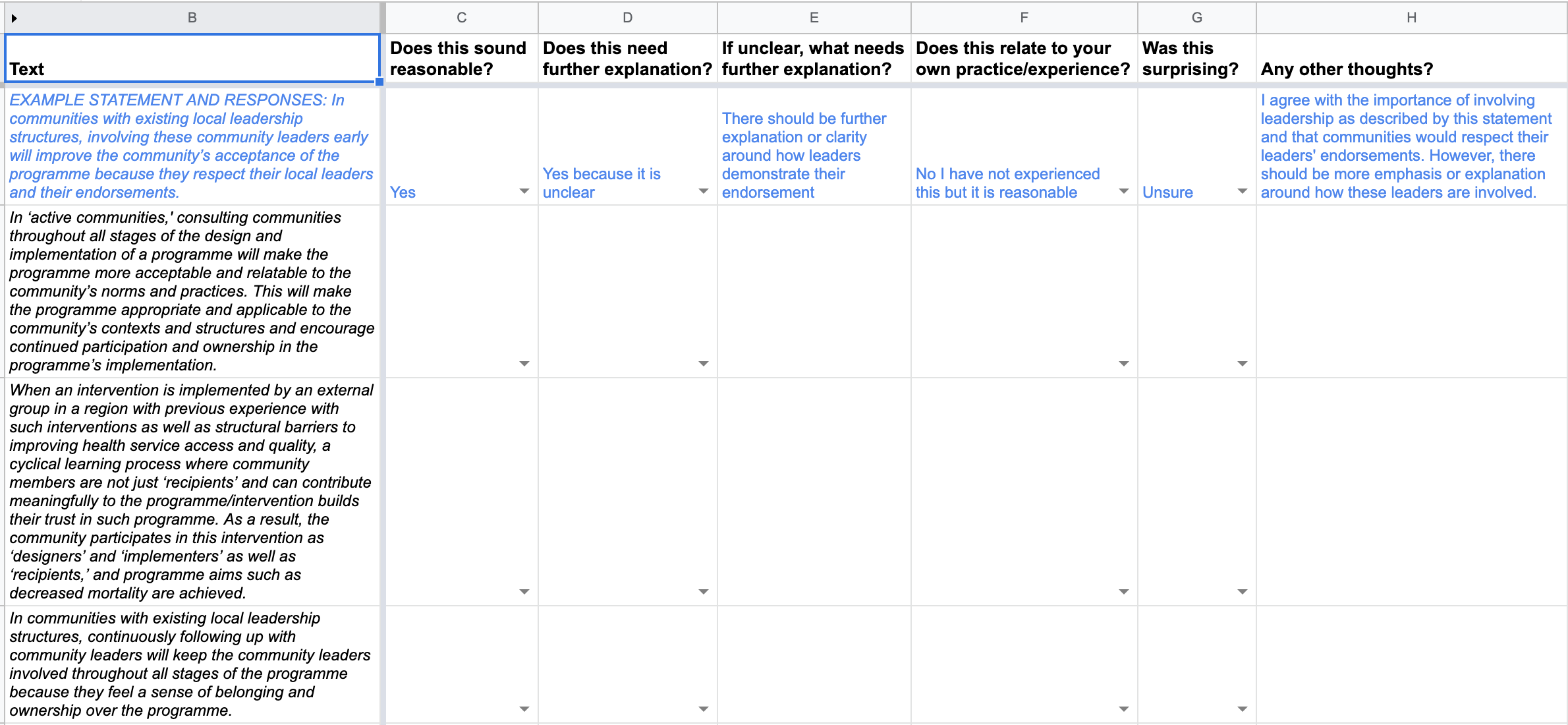
**

# Supplementary File 5: Low paper data extraction/confirmation

| **‘Theme’ 🡪**  ***Paper*** | **Relationships** | **Local Leaders** | **Communications Avenues** | **Tradition & Cultural Acceptability** | **Power & Fear** | **Peer Modelling & Linkage** | **Programme/ Service Provision** | **Sharing Experience Builds Knowledge & Confidence** | **Empower Community as Actors** |
| --- | --- | --- | --- | --- | --- | --- | --- | --- | --- |
| [*Arkedis et al. 2021*](https://www.ncbi.nlm.nih.gov/pmc/articles/PMC8085768/) | Not enough insight to pull out generative causation; describes community participation as the outcome | | | | | | | | |
| [*Azad et al. 2010*](https://www.sciencedirect.com/science/article/pii/S0140673610601420?via%3Dihub) | Describes the participatory women’s group intervention and quantitative/specific outcomes but not enough to interpret mechanisms | | | | | | | | |
| [*Bayley et al. 2015*](https://www.ncbi.nlm.nih.gov/pmc/articles/PMC4410129/) | **Power & Fear**  **support for CMOC 49 & 50**  **Relationships**  **support for CMOC 3** | | | | | | | | |
| [*Brasington et al. 2016*](https://journals.plos.org/plosone/article?id=10.1371/journal.pone.0151783) | **Communications Avenues**  **support for CMOC 15**  ***Edit CMOC15:***  **R1**: programme messaging and symbolism  **O1**: receptive to messaging  **O2**: influence attitude shifts  **Sharing Experience Builds Knowledge & Confidence**  **support for COMBINED CMOC 33 +59+60+61**  because also about how sharing experiences built support and knowledge for behavior change  **Empower Community as Actors**  **support for CMOC 10**  because about how an active/ motivated communities will take the intervention, adapt it and run with it for improved sustainability  ***Edit CMOC10:***  **C**: active and motivated communities | | | | | | | | |
| [*Dougherty et al. 2018*](https://www.ncbi.nlm.nih.gov/pmc/articles/PMC6157528/) | **Communications Avenues**  **support for edited CMOC 15**  because about how additional and repeated exposure essentially makes people talk about it more/change discussions/attitudes  **support for COMBINED CMOC 1+11+14+65**  because with increased and multiple exposures to messaging they are familiar, feel supported, and knowledge increases  ***Edit COMBINED CMOC 1+11+14+65:***  **O:** receiving these messages and having discussions in multiple forums  **Peer Modelling & Linkage**  **support for COMBINED CMOC 19+25**  ***Edit COMBINED CMOC 19+25:***  **R1**: having local champions or individuals influential in community networks share the messaging  **R2**: trust for these individuals | | | | | | | | |
| [*Ediau et al. 2013*](https://bmcpregnancychildbirth.biomedcentral.com/articles/10.1186/1471-2393-13-189) | Describes intervention and outcomes but not enough detail to explain mechanisms substantially…*potentially* support for CMOC 42 from mention of male change agents? | | | | | | | | |
| [*Ekirapa-Kiracho et al. 2020*](https://equityhealthj.biomedcentral.com/articles/10.1186/s12939-020-01184-6) | **Empower Community as Actors**  **support for COMBINED CMOC 34+35+37+62**  because collaborative problem solving and ownership  **Empower Community as Actors**  **support for CMOC 10**  because collaborative problem solving and ownership | | | | | | | | |
| [*George et al. 2018*](https://equityhealthj.biomedcentral.com/articles/10.1186/s12939-018-0838-5) | **Empower Community as Actors**  **support for COMBINED CMOC 34+35+37+62**  because about building knowledge and ownership which empowers community + developed tool participatory style (just doesn’t explicitly say ‘critical consciousness’)  **Tradition & Cultural Acceptability**  **support for COMBINED CMOC 32+40+48+64**  because relatable/understandable visuals  ***Edit COMBINED CMOC 32+40+48+64:***  **R1**: participatory activities and/or tailored messaging/visual representations | | | | | | | | |
| [*Gram et al. 2019*](https://www.ncbi.nlm.nih.gov/pmc/articles/PMC6540743/) | **Sharing Experience Builds Knowledge & Confidence**  **support for COMBINED CMOC 33 +59+60+61**  because about building confidence and how this leads to potential behaviour change/further dissemination  *🡪 look into societal norms around whether women can speak up in group settings/the men-women-household dynamic in community events or in households (similar to CMOCs 41 & 42).* | | | | | | | | |
| [*Manandhar et al. 2004*](https://www.sciencedirect.com/science/article/pii/S0140673604170219?casa_token=IF2ODx5b3KsAAAAA:kpGhnbMzSphwvcgAZJK5OVv03vSmJWFZ850wq89I5CMYxJ2p-KAsMq4lf8YhIsQ6M4SD7G_r) | **Tradition & Cultural Acceptability**  **support for COMBINED CMOC 32+40+48+64**  because about the adapted participatory nature of education increasing knowledge and learning | | | | | | | | |
| [*Mochache et al. 2018*](https://bmchealthservres.biomedcentral.com/articles/10.1186/s12913-018-3739-9) | **Empower Community as Actors**  **support for COMBINED CMOC 34+35+37+62**  because collaborative problem-solving to develop critical consciousness  **Empower Community as Actors**  **support for CMOC 10**  ***Edit CMOC 10:***  **R1**: when communities are consulted and own the programme from the design through the implementation  **R2**: the programme is acceptable  supports the health worker power dynamics context  *🡪 look more into intrinsic vs. extrinsic motivation* | | | | | | | | |
| [*Serbanescu et al. 2019*](https://www.ncbi.nlm.nih.gov/pmc/articles/PMC6519679/) | Supports a number of the previously identified resources such as using multiple communication avenues and exposure; Community members as active/actors; Engaging and involving local leaders early | | | | | | | | |
| [*Turan et al. 2003*](https://academic.oup.com/heapro/article/18/1/25/895087?login=false) | Supports the adaptation of communications materials as well as pre-testing these materials – made changes (feedback loop?) when messaging was not clear  power and control of community increasing over the project | | | | | | | | |
| [*Woelk et al. 2016*](https://www.ncbi.nlm.nih.gov/pmc/articles/PMC4754877/) | Describes intervention in fairly significant detail with a lot of similar resources to previously identified CMOCs, however not enough for new generative causation or to support the existing CMOCs: Community leaders are the ones really leading and conducting the work; Reference to that critical consciousness developed through social dialogues; Using range of communication avenues adapted to the context; Participatory/shared-experiences and learning in the peer groups  *🡪 look into final study published here: Bandopadhay et al 2021 -* [*https://link.springer.com/article/10.1007/s10461-021-03202-2*](https://link.springer.com/article/10.1007/s10461-021-03202-2) | | | | | | | | |
| ***Iterative Searching*** | | | | | | | | | |
| [*Howard-Grabman & Storti, 2014*](http://www.sbccimplementationkits.org/demandrmnch/wp-content/uploads/2014/02/Demystifying-Community-Mobilization-An-Effective-Strategy-to-Improve-Maternal-and-Newborn-Health.pdf) | **Empower Community as Actors**  Community is involved and engaged in the process of adapting the communication approach/messaging. Might not fit in the CMOCs in this demi-regularity specifically  **Tradition & Cultural Acceptability**  **support for CMOC 21**  **Programme/Service Provision**  **support for CMOC 44**  ***Edit CMOC 44:***  **R1:** when the community benefits from the programme and its messaging | | | | | | | | |
| [*Moran et al. 2006*](https://www.ncbi.nlm.nih.gov/pmc/articles/PMC3001153/) | **Empower Community as Actors**  **support for CMOC 1**  **Relationships**  **support for CMOC 27** | | | | | | | | |
| [*Okonofua et al. 2022*](https://bmjopen.bmj.com/content/12/2/e049499) | **Empower Community as Actors**  **support for CMOC 1**  **Local Leaders**  **support for CMOC 10 & CMOC 12** | | | | | | | | |
| [*Scott et al. 2017*](https://journals.plos.org/plosone/article?id=10.1371/journal.pone.0182982) | **Power & Fear**  **support for CMOC 16** | | | | | | | | |

# Supplementary File 6: CMOC organisation process


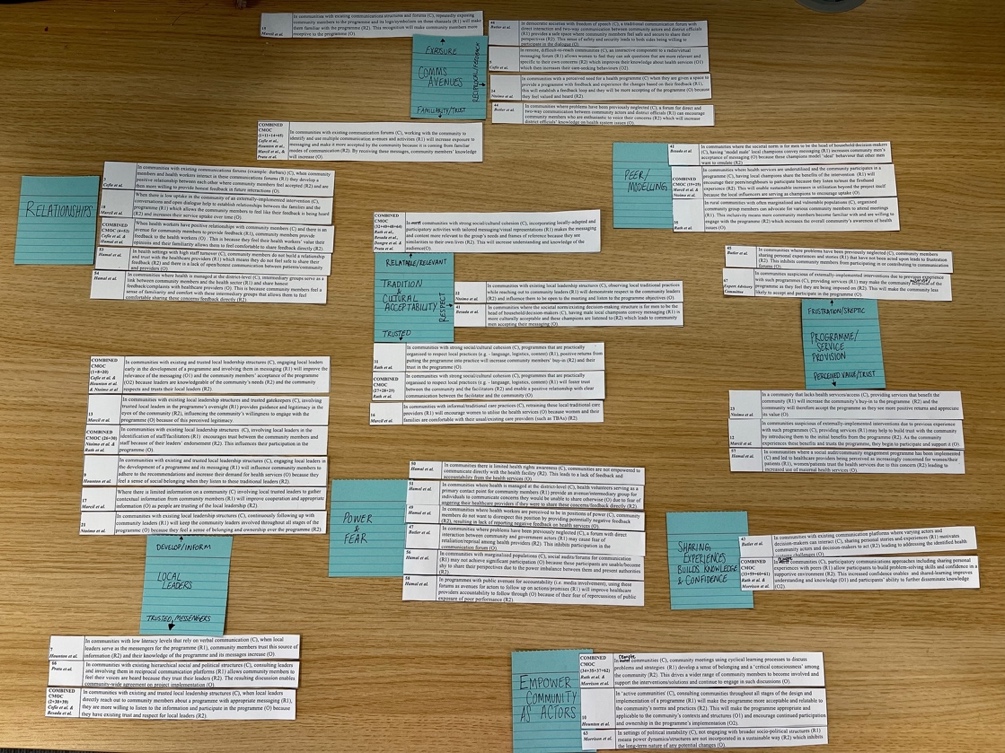


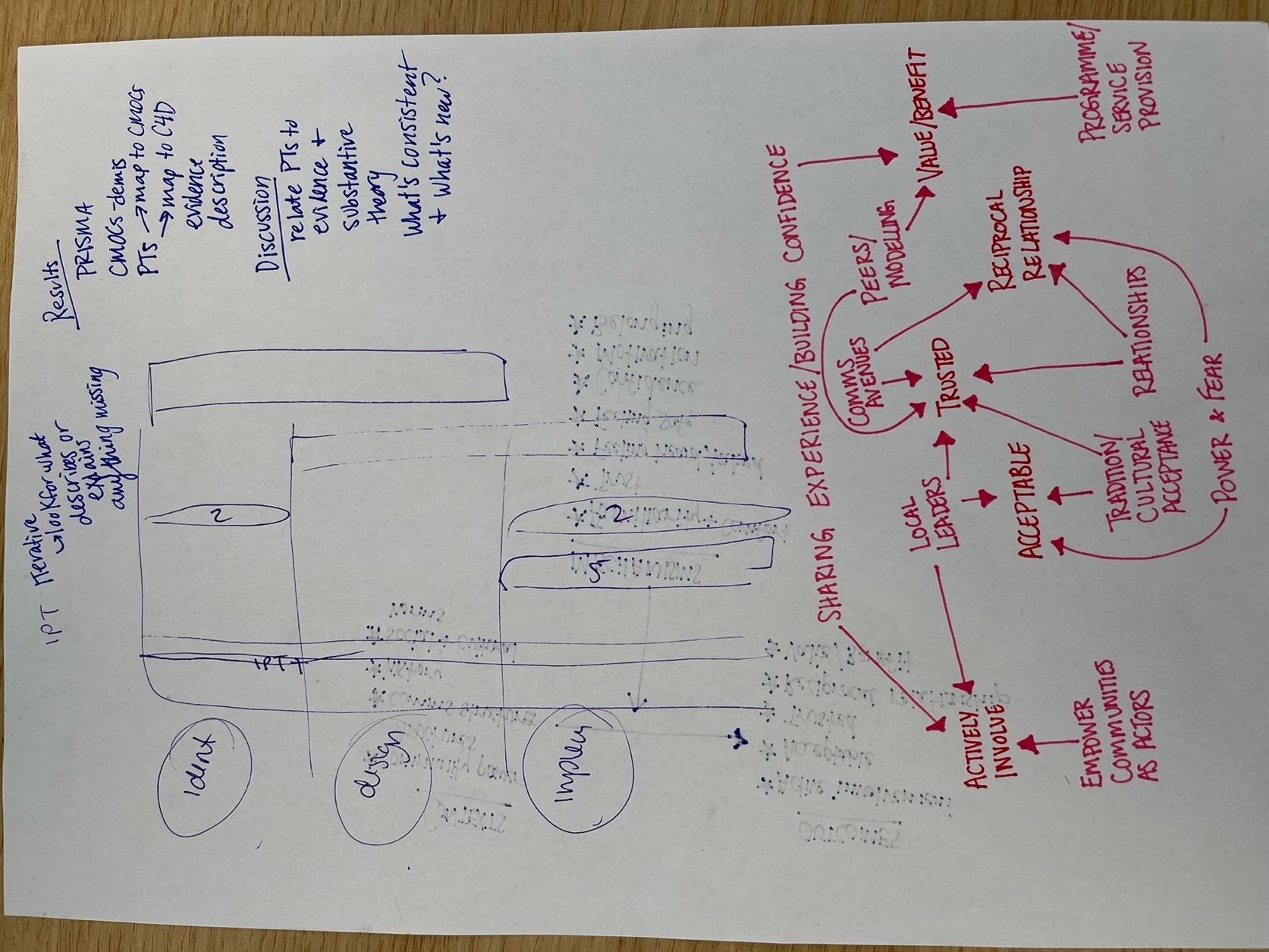
**
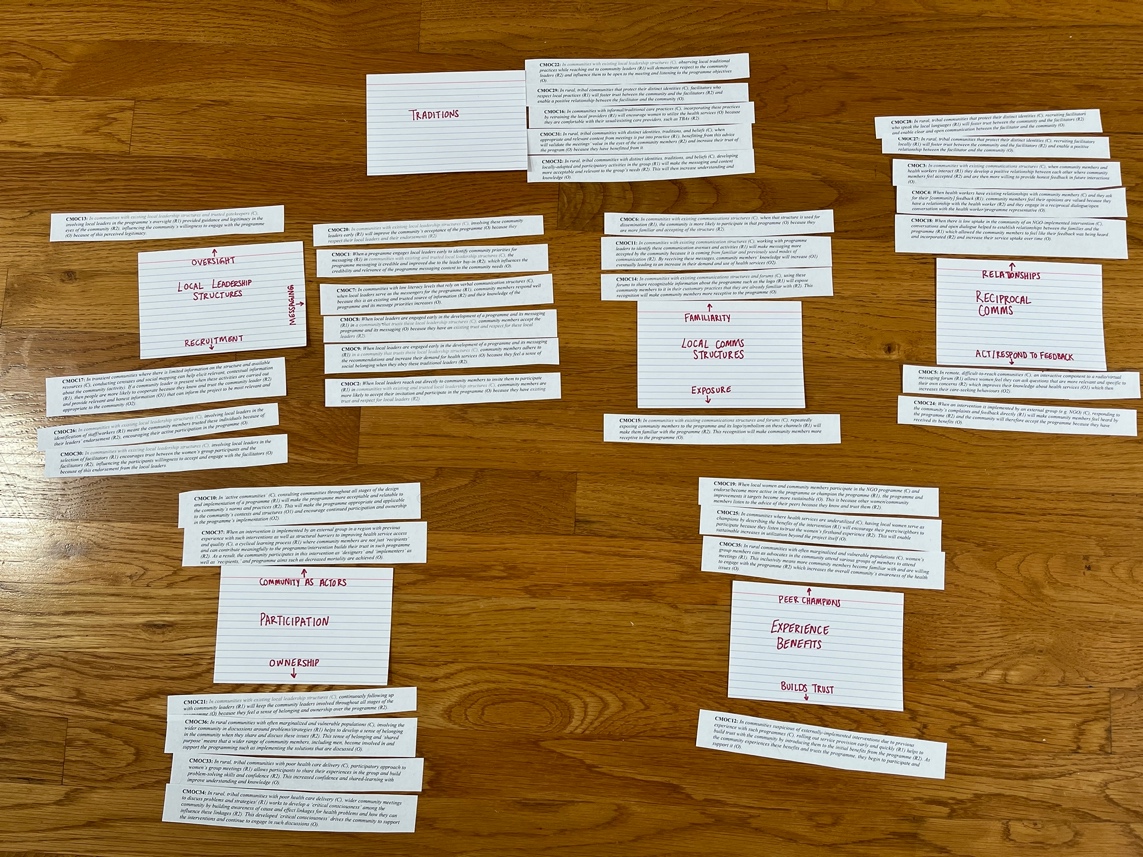
**

# Supplementary File 7: Initial programme theories identified in Phase 1 of the review (realist review protocol)

| **Initial Programme Theories** | |
| --- | --- |
| **IPT1:**  ***Identify*** | If the MNH intervention places the community at the centre of the programme by **involving community members and structures early and continuously** (for example through stakeholder/resource mapping, involving local leaders/influencers in the development of messaging priorities, or collaborating with local staff) and adjust messaging based on the community’s feedback, then the community (women, their families, and stakeholders) will be more **motivated to engage with the programme** because it will be **relevant** to their needs and concerns. This involvement, which takes place throughout both the development and implementation of the communication messaging, will encourage participation because it is contextually appropriate messaging that is delivered with/by community peers alongside existing infrastructure/resources which enhance the sustainability of the programme. |
| **IPT2:**  ***Design*** | If the MNH programme implementers use **multiple and accessible avenues** to communicate with the community about the MNH intervention on a regular basis with **messaging that is relatable and localised** then the programme’s messaging **will** **be easy to understand**. This will create multiple opportunities for community members to learn about and engage with the MNH programme. These **accessible avenues of communication** in addition to the **community’s awareness** of the intervention **enables community members to participate in a reciprocal dialogue** with the implementers. |
| **IPT3:**  ***Implement*** | If the MNH implementers **acknowledge local power structures, hierarchies, and governance** (for example, by approaching traditional/religious leaders and local influencers or using polite/appropriate language and titles in conversations) and **communicate through these existing community groups/structures**, **then this will foster a sense of trust and respect** between the community members and the implementers and encourage the community to engage further with the programme. Communicating through local structures and existing capacity produces a more sustainable impact and leads to greater influence and **better relationships with implementers** when these local systems are trusted by the communities. However, if these structures or local governments and systems are not trusted by the communities (e.g. – as a result of political/cultural conflict), then working with these groups may not be enough. |

# Supplementary File 8: Expert advisory committee feedback form for PTs


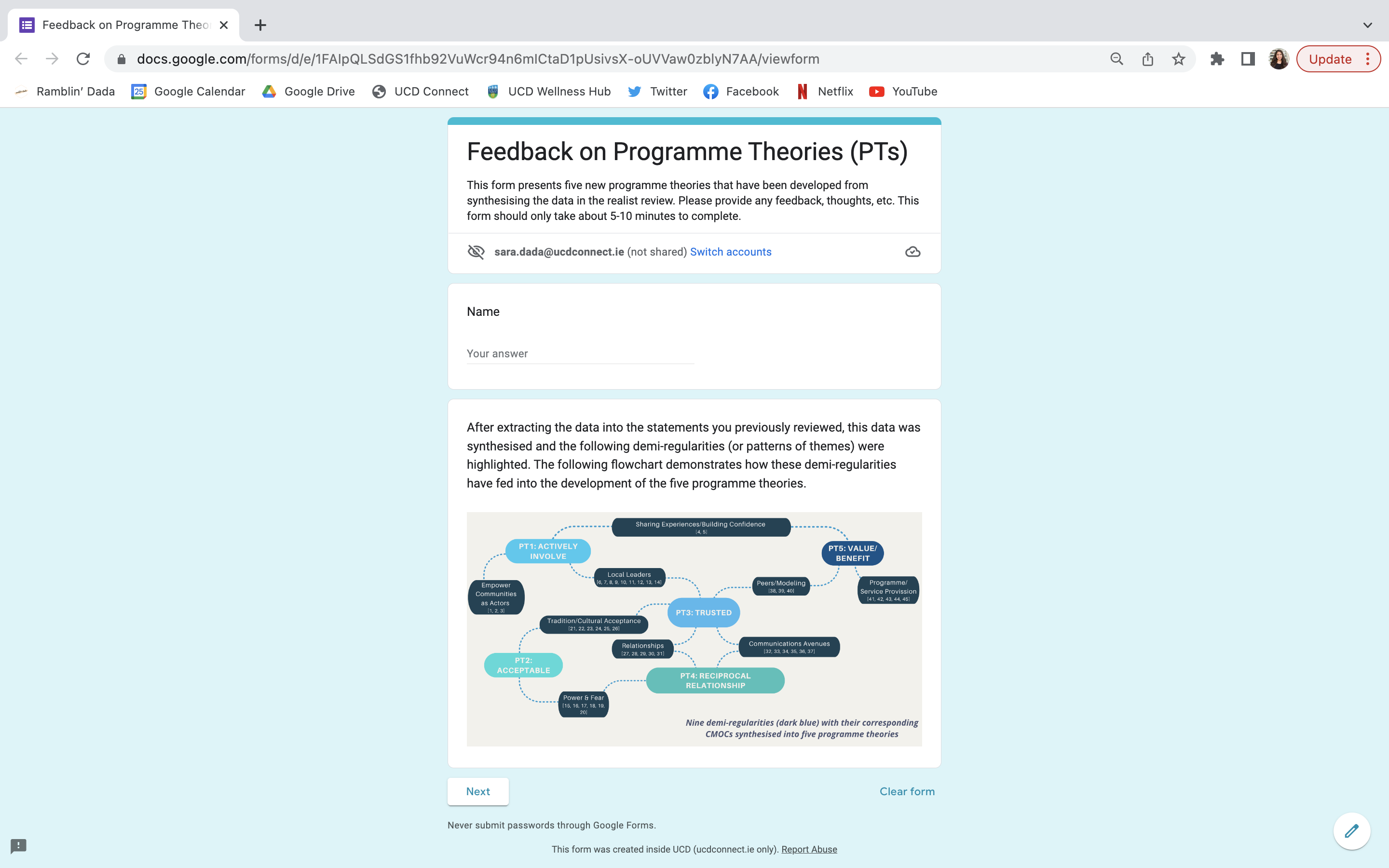


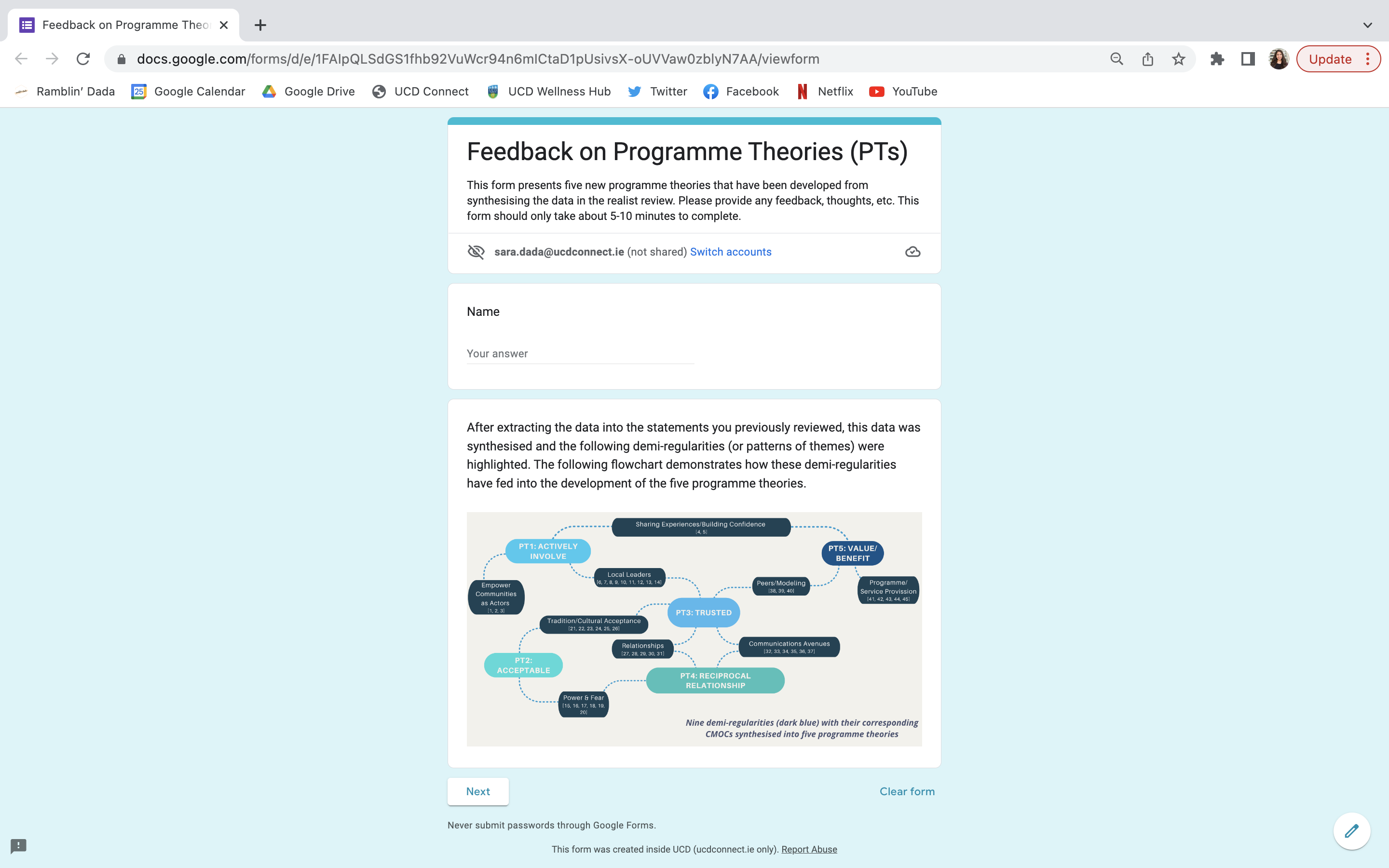


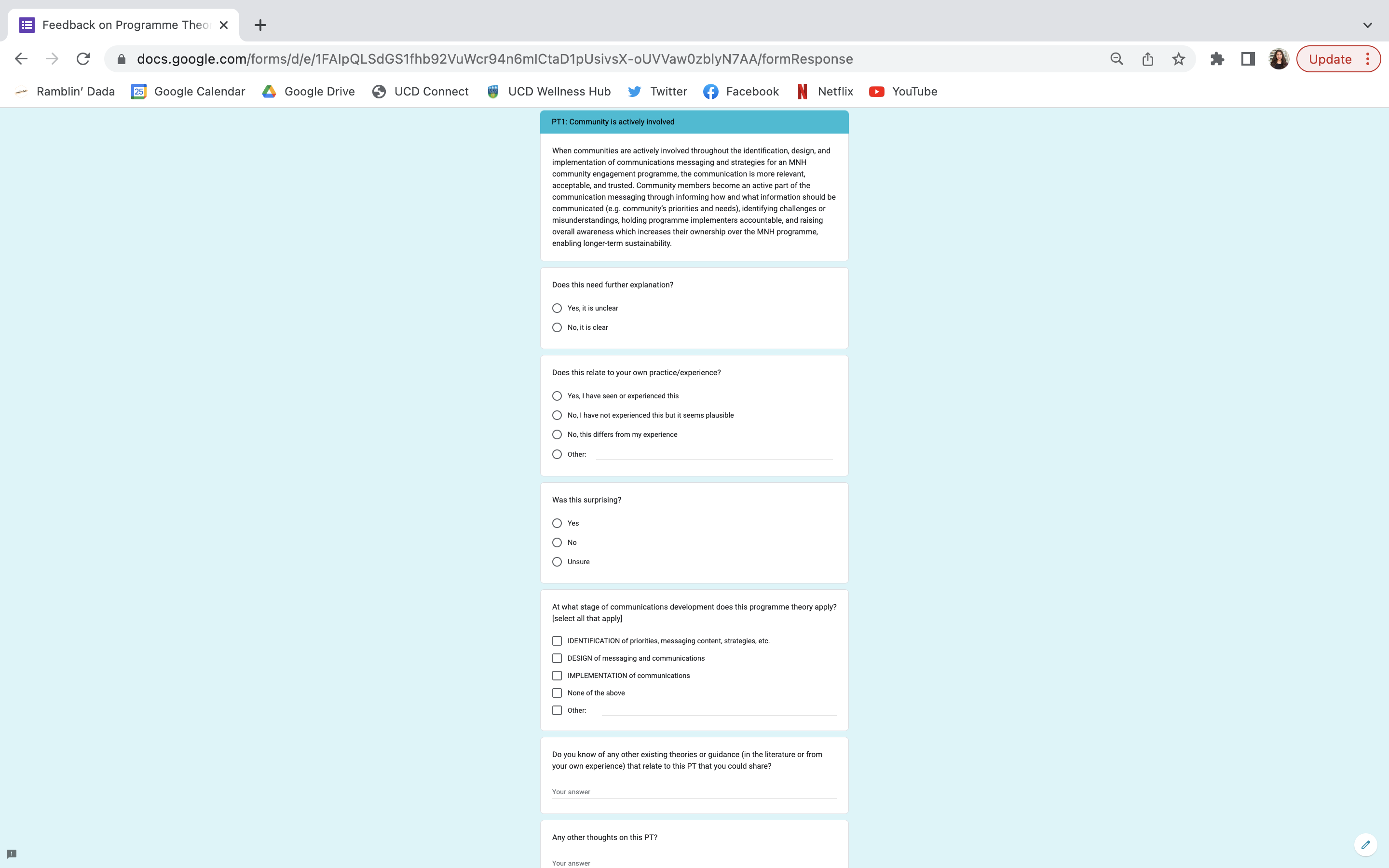


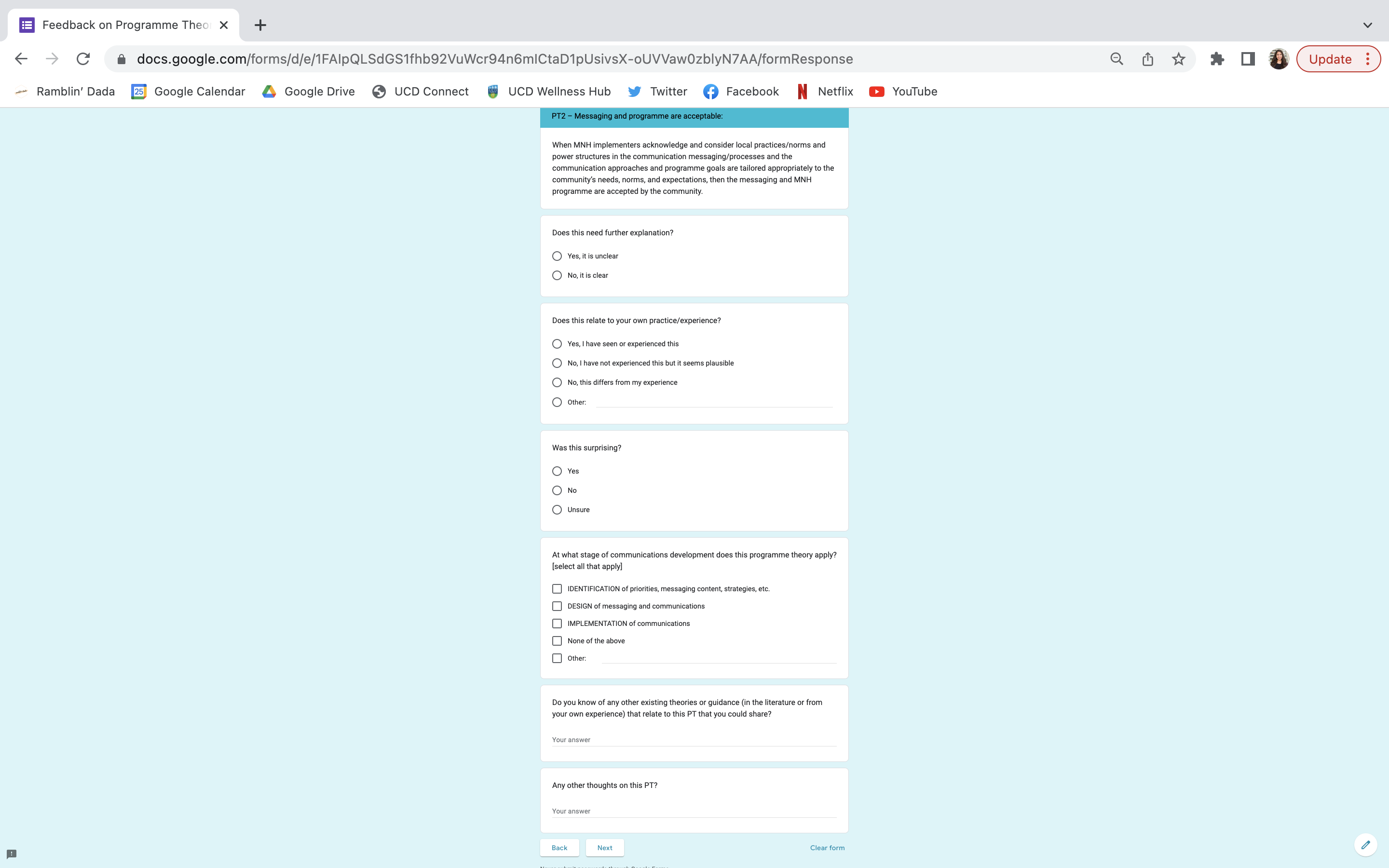


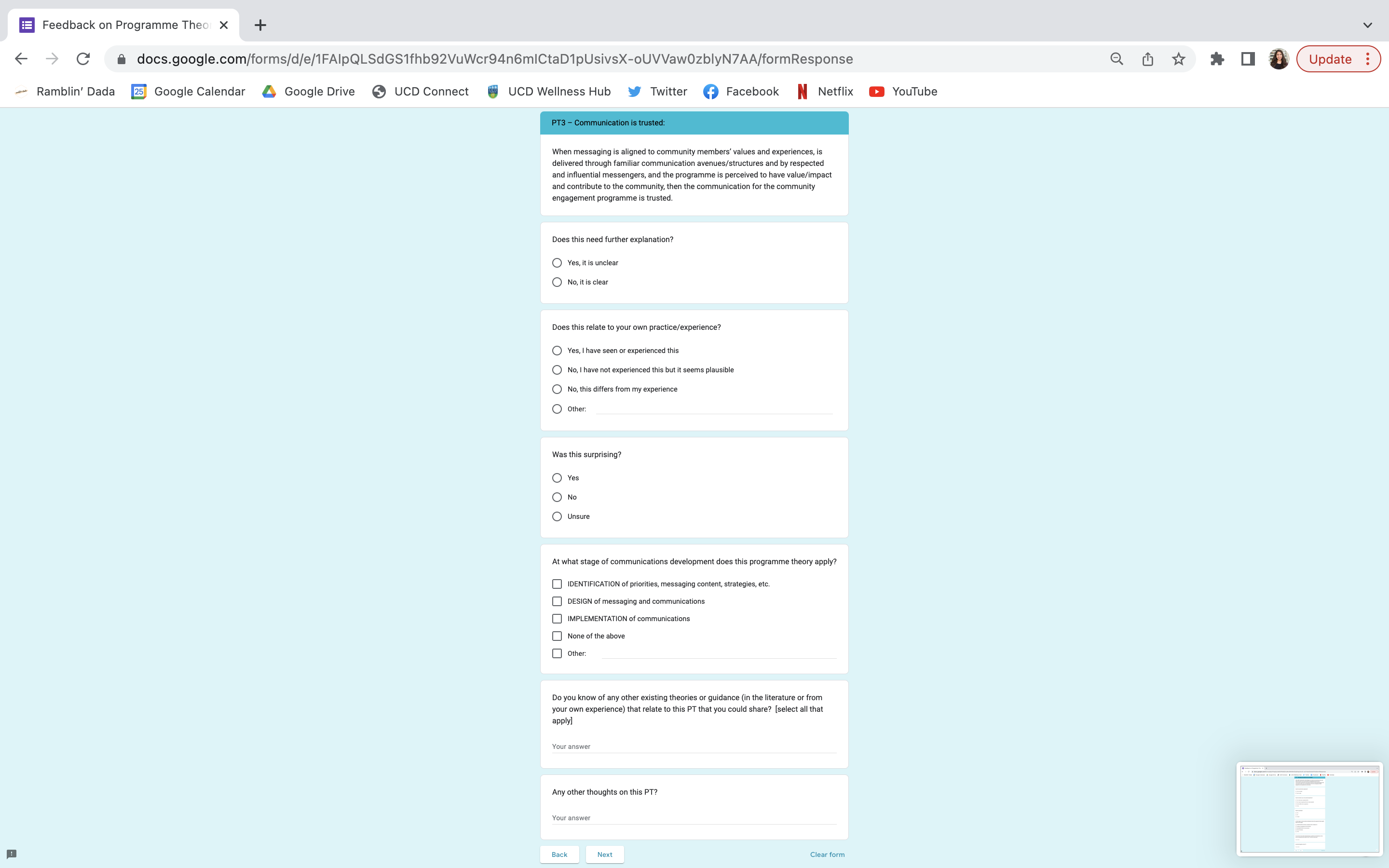

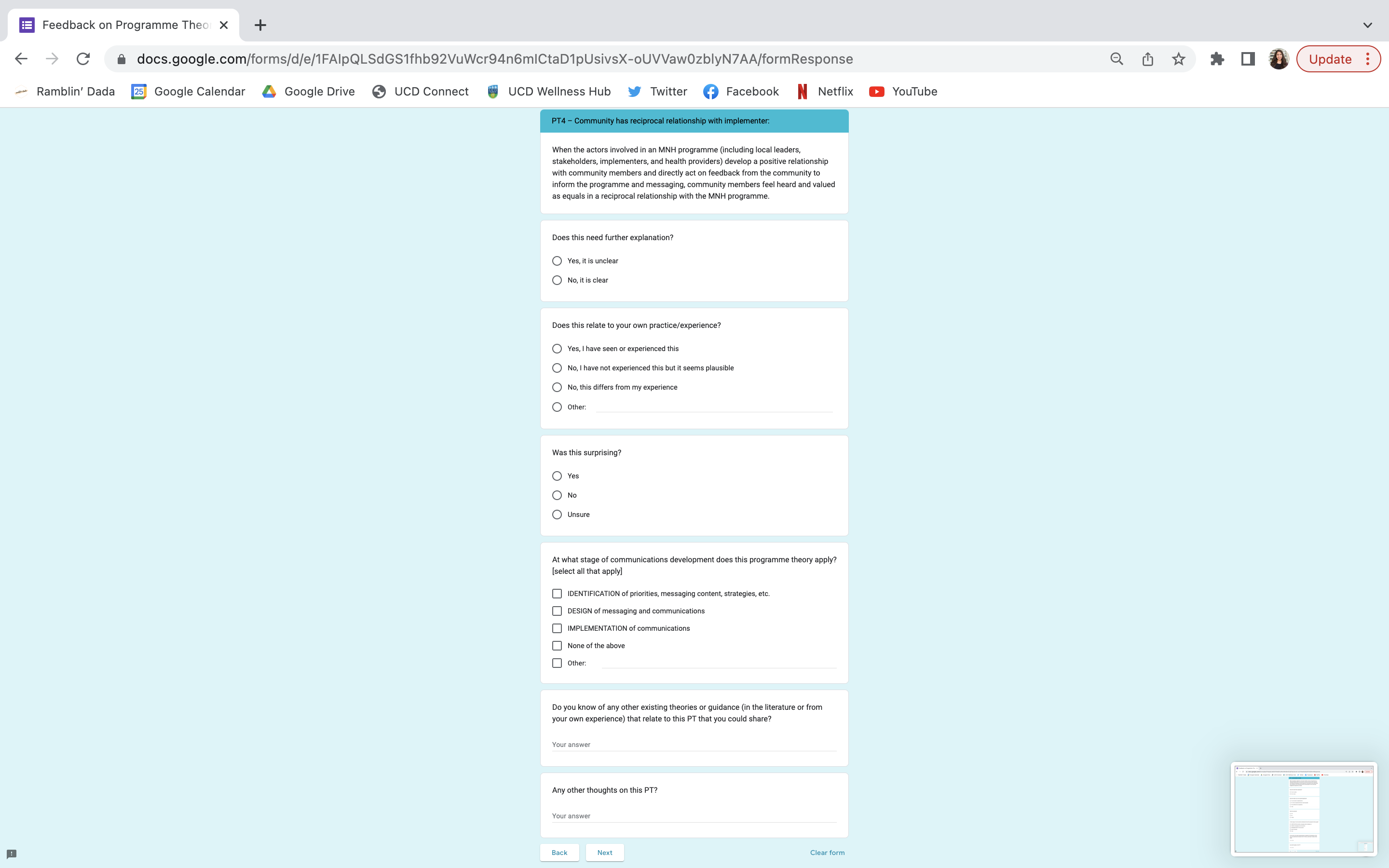

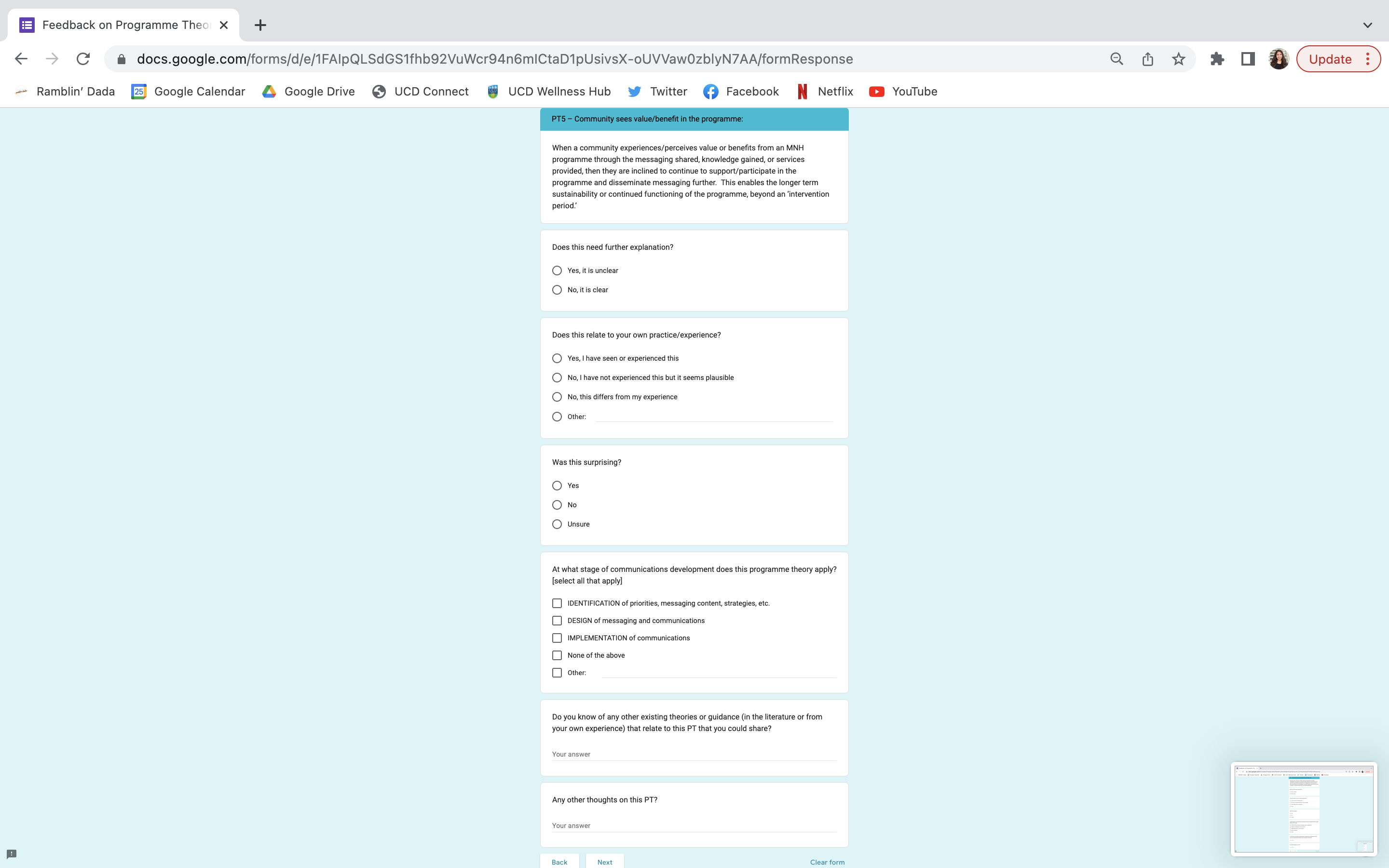


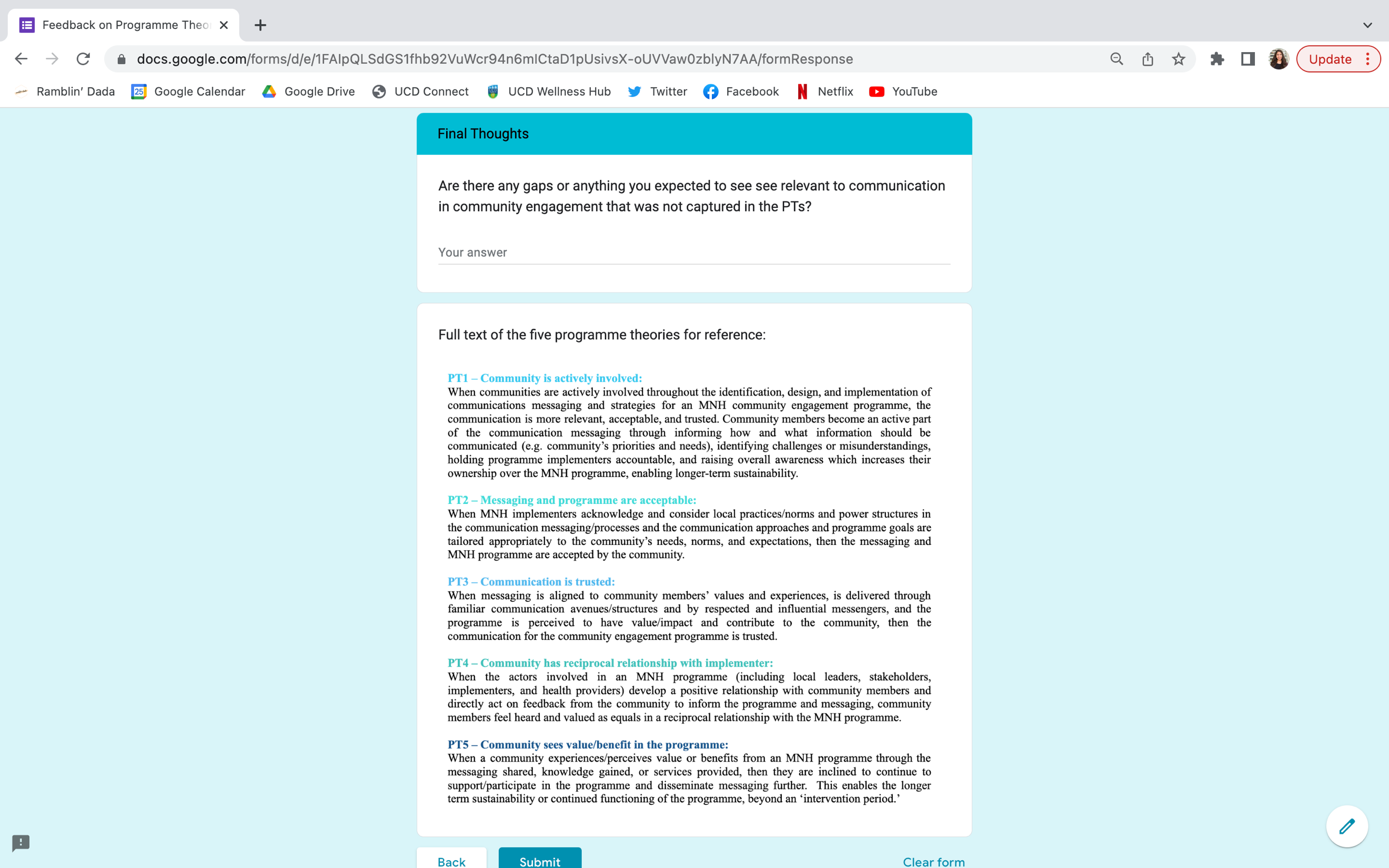


# Supplementary File 9: Description of CE communications in the 11 extracted studies

| **Author, Year** | **Country Setting** | **Description of CE Communications & Activities (as provided by the document)** |
| --- | --- | --- |
| *Cofie et al., 2015* | Ghana | 1) Community radio broadcasts  2) Community engagement and education at public gatherings (durbars)  3) home visits to pregnant women and mothers |
| *Hounton et al., 2009* | Burkina Faso | 1) Identifying key influential leaders  2) Engaging stakeholders  3) Implementing BCC activities such as workshops, concerts, theatre + monitor |
| *Marcil et al., 2016* | Bangladesh | 1) Meeting with local leaders and representatives,  2) Cyclical feedback  3) Mass marketing  4) Social mapping and census taking |
| *Ntoimo et al., 2021* | Nigeria | 1) Advocacy activities and engagement with key stakeholders  2) Community conversations  3) Ward development committees  4) Community sensitization workshops |
| *Rath et al., 2010* | India | 1) Women’s group (PLA cycle) with pregnant/recently pregnant women |
| *Besada et al., 2016* | Uganda, DRC, Malawi, Cote d’Ivoire | 1) Collaborating with leaders  2) Use existing CHW cadres  3) Peer support groups  4) Model clients/couples  5) Dedicated male/family support groups  6) Radio messages/Theatre  7) Male champions/action groups  **Activities focused on male involvement in PMTCT.* |
| *Butler et al., 2020* | Malawi | 1) Community and district bwalos (involving community members, political and administrative actors, and health actors/authorities)  **not very clearly described in the document* |
| *Dongre et al., 2009* | India | 1) Women’s self-help groups  2) Adolescent girl forums  3) Farmers’ clubs  4) Village coordination committees (included action experience learning cycle) |
| *Hamal et al., 2019* | Nepal | 1) Mothers groups (women of reproductive age, conduct monthly meetings facilitated by a local FCHV who shares health-related information which the mothers group members then share with community members)  2) Female community health volunteers (self-motivated women in the community who are trained on health education and promotion)  3) Health facility management committees (largely made up of a range of community representatives and leaders + representatives from health facility)  4) Other activities such as social audits and community score boards |
| *Morrison et al., 2010* | Nepal | 1) Women’s groups (PLA method) |
| *Prata et al., 2012* | Nigeria | 1) Women and communities educated about birth preparedness and PPH in community dialogues, dramas, and print materials  2) CORPs & TBAs trained to counsel women in individual & group education sessions  3) Community members trained as drug keepers to store + dispense misoprostol |
