# Supplementary material for: Understanding communication in community engagement for maternal and newborn health programmes in low- and middle-income countries: a realist review
Source: Health Policy Plan. 2023 Aug 31;38(9):1079–98. doi: 10.1093/heapol/czad078 (PMC10566325; doi:10.1093/heapol/czad078)
Supplement: czad078_Supp [file czad078_supp.zip › suppl_data/HPP reviewer comments round 2.docx]

Dear Editorial Team,

Thank you for sharing the below reviewer comments on our manuscript entitled **“Understanding What Works for Communications in Community Engagement for Maternal and Newborn Health Programmes in Low- and Middle-Income Countries: A Realist Review”** and considering it for publication in *Health Policy and Planning.*

We appreciate the opportunity to strengthen our manuscript based on the constructive feedback of the reviewers.

After incorporating these constructive comments from the reviewers, we believe this article will be a valuable contribution to *Health Policy and Planning* and its publication could contribute to meaningful conversations around a field of global health programming interventions that are being increasingly incorporated across the maternal and newborn health field. This realist review not only presents theories explaining communications for community engagement in maternal and newborn health programming, but also provides an unprecedented level of detail on the steps of conducting a realist review on a complex health intervention.

Finally, we would like to thank the reviewers for their incredibly helpful comments and feedback that we believe have strengthened the manuscript. Realist reviews can be challenging to conduct, and even more challenging to describe in a way that is clear and concise, and so we appreciate the reviewers’ time in contributing to the improvement of this manuscript. We hope that it will provide a useful example for future realist reviewers.

Please see our responses to the journal and reviewers’ comments below.

**Reviewer #1:** The authors have provided a detailed account of their responses to the reviewers and revised the manuscript effectively. The manuscript is now more internally consistent and the clarifications on concepts, methods, ranges of CE activities (incl. supp. file 9) and outcomes are appreciated. The discussion section has become more interesting with the section on substantive theory. In my opinion, the authors have strengthened the richness and contribution of the paper on both content and method levels. I have a few remaining editing comments that you may want to address in the final version:

**RESPONSE:** Thank you so much to the reviewer for the feedback and suggestions that have helped to strengthen the paper. We agree that the clarity provided has truly improved the manuscript and appreciate the time taken to provide such a thorough review.

1. Background: the positioning and problematization of communications in CE is now much clearer. The addition of definitions is helpful. The phrase “CE communication’ is operationalized as the communication between, or amongst, a community in regards to a CE approach” (lines 63-64) does not seem correct “communication between a community” – is it “members of a community” or “communities”?

**RESPONSE:** We have clarified this to read as members of a community (**page 2, line 62**).

2. Also, in the section (lines 63-86) I miss the communicating actors. I understand communication as (at least) a two-way or two party interaction/dialogue. I see that “the community” is a central actor, but are health actors/programmers/providers not concerned as the dialogue partner? In the objective you clearly speak about MNH programming so I suggest to clarify that CE communications in your review is about communications between communities and health programmers/implementers. Also to speak to the main audience of the paper.

**RESPONSE:** We have addressed this by clarifying the description that was also mentioned in comment #1 to specify that this communication happens between community members and the programme (**page 2, lines 62-63**).

3. Line 247: synthesis should be synthesizing

**RESPONSE:** Thank you to the reviewer for catching this typo, we have fixed it (**page 5, line 212**).

4. Line 255: …existing IPTs developed in Phase 1…I suggest to specify “IPTs developed in the protocol in Phase 1”. For the authors it may feel as over-explanation but for an external reader this is helpful to understand the sequence.

**RESPONSE:** We have incorporated this clarification (**page 6, line 219**).

5. Line 461: unclear formulation: “were developed from programming in this health topic”

**RESPONSE:** We have clarified this language by specifying the PTs were developed based on literature in MNH (**page 10, line 422**).

6. Figure 1: not sure why there is a c before IPTs

**RESPONSE:** This has been removed for clarity/consistency.

7. Figure 4: you may want to adapt the third: trusted communication sources

**RESPONSE:** This has been adapted.
